# Postpartum changes in maternal physiology and milk composition: a comprehensive database for developing lactation physiologically-based pharmacokinetic models

**DOI:** 10.3389/fphar.2025.1517069

**Published:** 2025-02-03

**Authors:** Neel Deferm, Jean Dinh, Amita Pansari, Masoud Jamei, Khaled Abduljalil

**Affiliations:** ^1^ Predictive Technologies Division, Certara UK Limited, Sheffield, United Kingdom; ^2^ Pharmides BV, Pelt, Belgium; ^3^ School of Pharmacy, Department of Pharmacy, University of Washington, Seattle, WA, United States

**Keywords:** lactation, PBPK, postpartum, meta-analysis, milk, breastfeeding

## Abstract

**Introduction:**

Pharmacotherapy during lactation often lacks reliable drug safety data, resulting in delayed treatment or early cessation of breastfeeding. *In silico* tools, such as physiologically-based pharmacokinetic (PBPK) models, can help to bridge this knowledge gap. To increase the accuracy of these models, it is essential to account for the physiological changes that occur throughout the postpartum period.

**Methods:**

This study aimed to collect and analyze data on the longitudinal changes in various physiological parameters that can affect drug distribution into breast milk during lactation. Following meta-analysis of the collated data, mathematical functions were fitted to the available data for each parameter. The best-performing functions were selected through numerical and visual diagnostics.

**Results and Discussion:**

The literature search identified 230 studies, yielding a dataset of 36,689 data points from 20,801 postpartum women, covering data from immediately after childbirth to 12 months postpartum. Sufficient data were obtained to describe postpartum changes in maternal plasma volume, breast volume, cardiac output, glomerular filtration rate, haematocrit, human serum albumin, alpha-1-acid glycoprotein, milk pH, milk volume, milk fat, milk protein, milk water content, and daily infant milk intake. Although data beyond 7 months postpartum were limited for some parameters, mathematical functions were generated for all parameters. These functions can be integrated into lactation PBPK models to increase their predictive power and better inform medication efficacy and safety for breastfeeding women.

## 1 Introduction

Many postpartum women need to take medications for either chronic or postpartum related conditions (Saha et al., 2015). However, the limited information on drug safety during lactation presents challenges for both healthcare providers and patients when making informed benefit-risk decisions about medication use while breastfeeding (Scime et al., 2023). Various approaches, including animal models, have been investigated to better understand how drug properties and the dynamic maternal physiology influence drug distribution into breast milk (PRGLAC, 2018). Yet, due to differences in milk composition across species, developing animal models that accurately predict drug exposure in human breast milk remains difficult (Stebler and Guentert, 1990).

Alternative approaches, like physiologically-based pharmacokinetic models, can help bridge this knowledge gap. These models consider the complex interactions between drug properties and physiological parameters in the target population, and can be used to predict drug concentrations in various tissues (Jones and Rowland-Yeo, 2013). In addition, PBPK models can be adapted to predict drug transfer into breast milk by incorporating data on breast and milk properties (Abduljalil et al., 2021; Pansari et al., 2022). While these models have provided accurate predictions for several compounds, they often assume that physiological parameters remain constant throughout the postpartum period. However, this assumption does not reflect reality as the mother’s physiology undergoes significant changes during pregnancy and gradually returns to pre-pregnancy levels after childbirth (Abduljalil et al., 2012). Indeed, the postpartum period is highly dynamic, with various maternal physiological parameters returning to their baseline at different rates.

For example, glomerular filtration rate (GFR), which increases during pregnancy, decreases from an immediate postpartum value of 152 ± 34 mL/min to 92 ± 15 mL/min within 2 months, to then gradually rise and return to pre-pregnancy levels by 5 months postpartum (Dunlop, 1981; Hladunewich et al., 2004; Ahmed et al., 2009). Haematocrit levels, on the other hand, decrease during pregnancy and reach pre-pregnancy levels within just 2 weeks after childbirth and remain constant throughout the rest of the postpartum period (Taylor et al., 1981).

Moreover, during lactation, the physicochemical attributes of breast milk also undergo significant changes, especially in the early days after delivery. For instance, in the first few days postpartum, the mother produces colostrum, a protein-rich, low-fat milk with a pH close to that of extracellular fluid. As lactation progresses, mature milk is produced, which contains lower protein levels but has a higher fat content and a more acidic pH (Morriss et al., 1986; Bulut et al., 2019). These changes can substantially influence drug concentrations in breast milk and the amount of drug an infant receives. For example, clinical studies have shown that a two-fold increase in milk fat content (from 3.1 to 6.2 g/dL) was associated with a 28% and 18% increase in the transfer of escitalopram and its dimethyl metabolite into breast milk (Weisskopf et al., 2020). Modeling studies also suggest that weakly basic drugs, such as fluoxetine, accumulate more in breast milk during the later postpartum period due to the lower pH compared to earlier stages (Pansari et al., 2022). These findings indicate that assuming constant values for time-varying parameters in PK models can lead to inaccurate predictions of drug exposure in breast milk. Even assigning random parameter values within physiological ranges can affect the quality of model predictions, as demonstrated for milk fat and pH (Abduljalil and Faisal, 2024).

Consequently, to improve the accuracy of lactation PBPK models, it is essential to account for longitudinal changes in these physiological parameters throughout the postpartum period. Therefore, this study aims to review, collate, and analyse publicly available literature on these parameters to expand the database necessary for developing a realistic lactation PBPK model. Furthermore, this study provides continuous mathematical functions that can be integrated into these models to improve and enhance their predictive capabilities.

## 2 Materials and methods

### 2.1 Software

Data analysis was performed using Microsoft Excel 2016 (Microsoft Corporation, Microsoft Office Professional Plus 2016, https://products.office.com), and model fitting was performed using Excel Solver, available as a Microsoft Office Excel add-in program (Microsoft Corporation, Microsoft Office Professional Plus 2013, https://products.office.com). Plots were created using the free software R (version 4.3.2, R Foundation for Statistical Computing, Vienna, Austria, www.r-project.org). The GetData Graph Digitizer (version 2.26.0.20) was used to extract data from plots and convert these to numerical values.

### 2.2 Data sources

A separate search on PubMed (https://www.ncbi.nlm.nih.gov/pubmed/) and Google Scholar (https://scholar.google.com/) was conducted for each parameter using at least two keywords (* denotes wildcard character): the first keyword was linked to the postpartum period (e.g., “postpart*,” “postnat*,” “lactating” or “breastfeeding”), while the second keyword specified the parameter of interest (e.g., “breast milk composition,” “milk pH,” “milk fat,” “creamatocrit,” “plasma volume,” “albumin” “milk volume,” “breast volume” or “cardiac output”). The reference list of each selected article was manually searched for possible additional references. No language or date restrictions were applied.

### 2.3 Inclusion and exclusion criteria

Studies identified in the literature search were included for further analysis if they met the following inclusion criteria: 1) healthy breastfeeding women, 2) adult individuals between 18 and 45 years of age, 3) no medication use during or after pregnancy, 4) pregnancies were uncomplicated, 5) data recorded up to 12 months postpartum and 6) data for full-term infants. Studies were excluded if they specifically focused on preterm infants or included data that could be confounded by preterm infant data, if the study methods were unclear or inadequate for estimating the parameter of interest, or if data were presented in unclear format (e.g., a 10-day average of milk volume over the first 10 days postpartum). In case of studies with mixed scenarios, for example, data that was reported from mixed preterm and full-term pregnancies, data were used only if inclusion criteria (in this case full-term pregnancy cases) represent at least 90% of the cases in that study. In general, the inclusion of studies was prioritized whenever possible to capture the variability likely observed during the postpartum period, assuming a healthy pregnancy.

### 2.4 Data analysis

Data were compiled in a Microsoft Excel 2016 spreadsheet and reported as mean (x) and standard deviation (SD), standard error (SE), or coefficient of variation (CV) from n samples. Mean values were stratified for postpartum age per month. If a range was specified for the postpartum period, the midpoint of the range was used. Additionally, reported units were converted to a unified unit. Data from multiple studies were combined as described previously (Abduljalil et al., 2012). Specifically, a weighted mean value ( $\bar{X}$ ) was calculated using the following formula (Equation 1):

$$\bar{X}=\frac{\sum_{j=1}^{J}n_{j}x_{j}}{\sum_{j=1}^{J}n_{j}}$$ (1)

where n_j_, represents the number of subjects of study j and x_j_, the mean value of the given study. The overall sum of squares (SS), overall SD and overall CV for the weighted mean were calculated as follows (Equation 2):

$$\text{Overall SS}=\sum_{j=1}^{J}\left[\;\left[{\left({\text{SD}}_{j}\right)}^{2}+{\left(x_{j}\right)}^{2}\right]n_{j}\right]-N\;{\bar{X}}^{2}$$ (2)

Here, SD_*j*_ is the standard deviation from the *j*th study, and N is the total number of subjects in all studies. In addition, overall SD and CV were calculated as follows (Equations 3, 4):

$$\text{Overall SD}=\sqrt{\frac{\text{Overall}\;\text{SS}}{N}}$$ (3)

$$\text{Overall CV}=\frac{\text{Overall}\;\text{SD}}{\bar{X}}$$ (4)

Data were combined without accounting for differences in analytical techniques, feeding frequencies, and maternal demographics (i.e., age, weight and height). A set of one-dimensional functions was fitted to the observed data using postpartum age as a dependent variable. Weighted least squares regression was generally used, with each data point weighted by the number of study subjects. Linear, exponential, and polynomial functions up to the 4th degree along with various sigmoidal functions were considered to describe the data. The best-performing function was selected based on visual inspection and numerical diagnostics (i.e., weighted sum of squares). For example, if the data indicated an exponential reduction from a baseline, a linear model was not considered. Similarly, if the data for a specific parameter suggested a sigmoidal pattern, linear and polynomial equations were not considered. When comparing polynomial equations, if a higher-order polynomial (n) did not outperform a lower-order polynomial (n-1) based on r^2^ and the weighted sum of squares, the lower-order polynomial (n-1) was selected. If data were unavailable for the later stages of the postpartum period, simulated data points representing non-pregnant and non-lactating women were introduced to ensure that the function returned to baseline at 12 months postpartum. Additionally, variability was accounted for by applying a constant CV to the parameter of interest based on the variability observed in the data.

## 3 Results

### 3.1 Database characteristics

**FIGURE 1:**
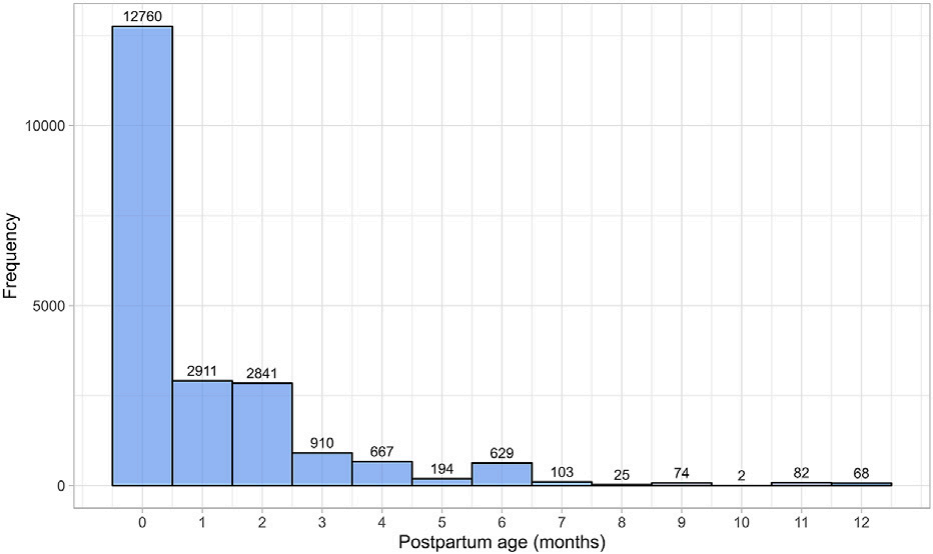
Frequency distribution of the collected data, stratified by postpartum age in months. The width of the bins is equal to 1 month.

The literature search identified 230 studies, with a total of 36,689 data points from 20,801 women in the postpartum period (Supplementary Table 1). The women included in these studies had a weighted mean age of 28.59 years (range: 20.8–40 years), a weighted mean weight of 63.93 kg (range: 45–100.40 kg), and a weighted mean height of 163.78 cm (range: 149–173.50 cm). Data were collected from immediately after childbirth up to 12 months postpartum. Figure 1 provides an overview of the data frequency across this period showing that most data points (60%) were reported within the first month postpartum. Beyond this, the amount of collected data decreased significantly, with particularly sparse data (n = 251 data points) available after 7 months postpartum. Moreover, the quantity and quality of the collected data differed substantially between the searched parameters. In addition, as many subject-specific characteristics, such as parity, ethnicity, or delivery type, were not consistently reported, these covariates were not considered in the analysis.

### 3.2 Milk properties

#### 3.2.1 Milk volume

Milk volume data were gathered from 11 publications, comprising 312 data points from 763 mothers (Supplementary Table 1). For this analysis, only data from mothers who were exclusively breastfeeding and whose children were born at term were used to estimate daily milk volume as a function of postpartum age. Since milk production is correlated with continued nursing, focusing on exclusively breastfed children helps estimate the worst-case scenario for daily infant drug intake through breastfeeding. Preterm births were excluded, as evidence suggests that milk production is lower in such cases (Hill et al., 2005).

**FIGURE 2:**
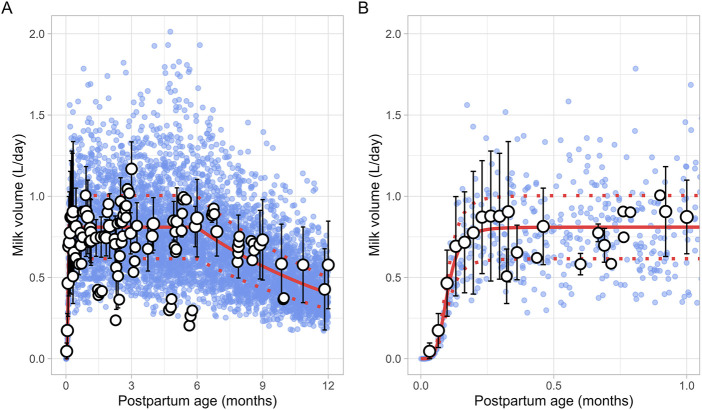
Simulated versus observed daily milk volume production for **(A)** the entire age range and **(B)** for 0 through 1 month postpartum. The blue dots depict simulated values (n = 5,000) generated using the Simcyp Simulator V23R2, and the white dots represent the observed data. Error bars indicate standard deviations, and the dot sizes correspond to the number of samples in the relevant studies. The solid red line is the mean value of the simulated data, whereas the dotted red lines are the standard deviations.

Following birth, the daily volume of milk the mother produces rapidly increases during the first few weeks postpartum. The daily milk production volume remains steady at this maximum until approximately 6 months postpartum, after which there is an exponential decline (Figure 2). The increase in milk production from birth to 6 months postpartum was best modeled by a sigmoidal equation (Equation 5):

$$\text{Milk volume }\left(L/\text{day}\right)=\frac{0.81\times {\text{PpT}}^{4.37}}{0.1^{4.37}+{\text{PpT}}^{4.37}}$$ (5)

Subsequently, for 6 through 12 postpartum months, the milk volume production was best described using a mono-exponential function (Equation 6):

$$\text{Milk volume }\left(L/\text{day}\right)=1.619\;e^{\left(‐0.116\;\times \;\text{PpT}\right)}$$ (6)

where PpT represents postpartum age in months. A constant CV of 33% was required to recover the variability in observed data.

#### 3.2.2 Milk pH

**FIGURE 3:**
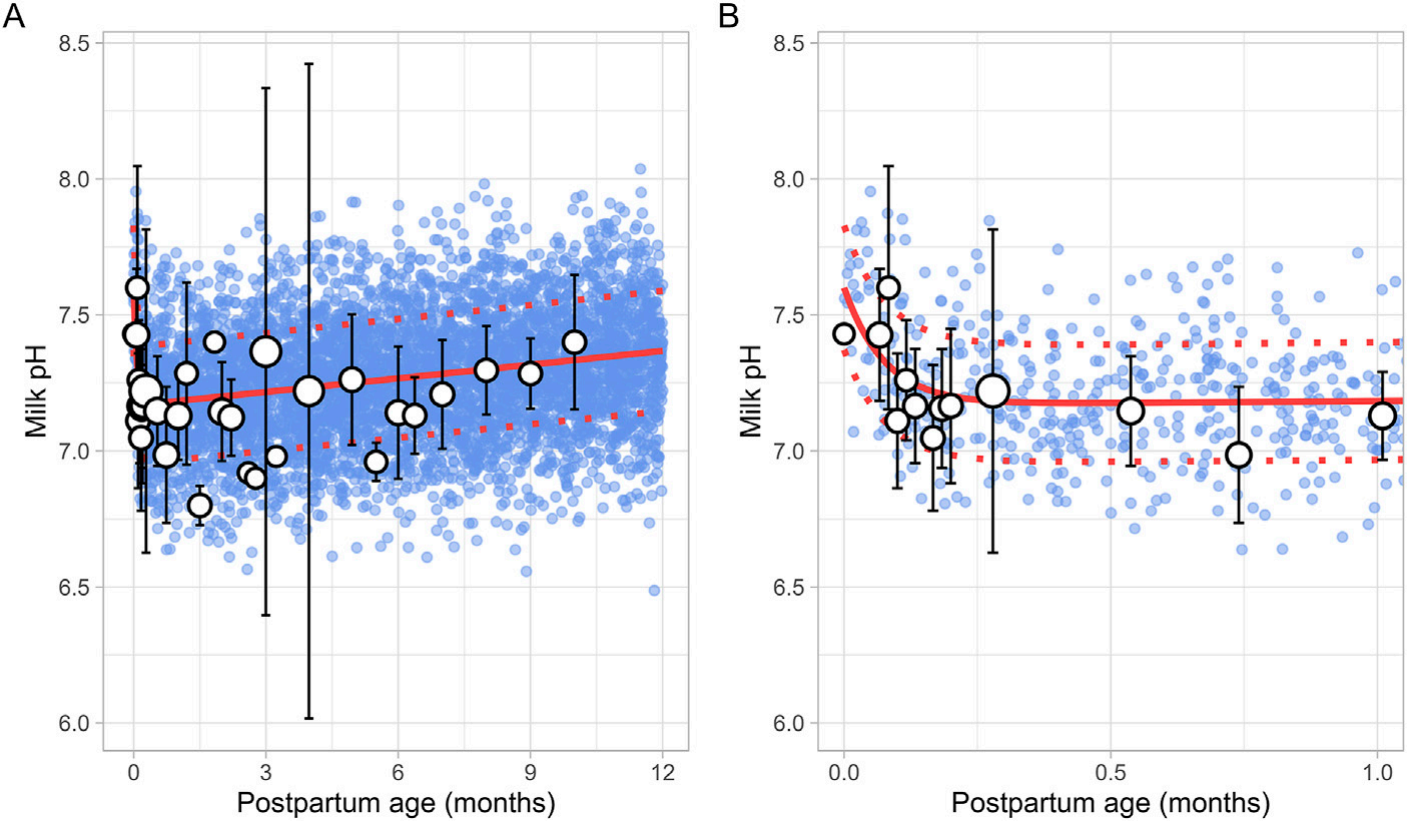
Simulated versus observed milk pH values for **(A)** the entire age range and **(B)** for 0 through 1 month postpartum. The blue dots depict individual simulated values (n = 5,000) generated using the Simcyp Simulator V23R2, and the white dots represent the observed data. Error bars indicate standard deviations, and the dot sizes correspond to the number of samples. The solid red line is the mean value of the simulated data, while the dotted red lines are the standard deviations.

A total of 15 studies on milk pH were identified that provided 790 data points from 328 postpartum women, with data collected from 2 days to 10 months postpartum (Supplementary Table 1). Only milk pH measurements were included from fresh samples that were analysed within 48 h and stored at temperatures between −80°C and 4°C. In addition, as previous studies have shown that extended storage may impact milk pH, data from milk bank samples were excluded (Ogundele, 2002; Slutzah et al., 2010). The data collected showed a shift in milk pH, starting at 7.43 ± 0.24 (mean ± SD) in colostrum, gradually declining to a low of 7.05 ± 0.27 around 2 weeks postpartum. It then slowly increased again as mature milk was produced (Figure 3). The data followed a double exponential decay function (Equation 7), indicating that after initial fluctuations in the early postpartum period, milk pH stabilizes with only minor variations during the later stages of lactation.

$$\text{Milk pH}=0.443\;e^{\left(‐13.07\;\times \;\text{PpT}\right)}+{\;7.167\;e}^{\left(0.0023\;\times \;\text{PpT}\right)}$$ (7)

Where PpT represents postpartum age in months. A constant CV of 3% was required to recover the observed variability.

#### 3.2.3 Milk fat

**FIGURE 4:**
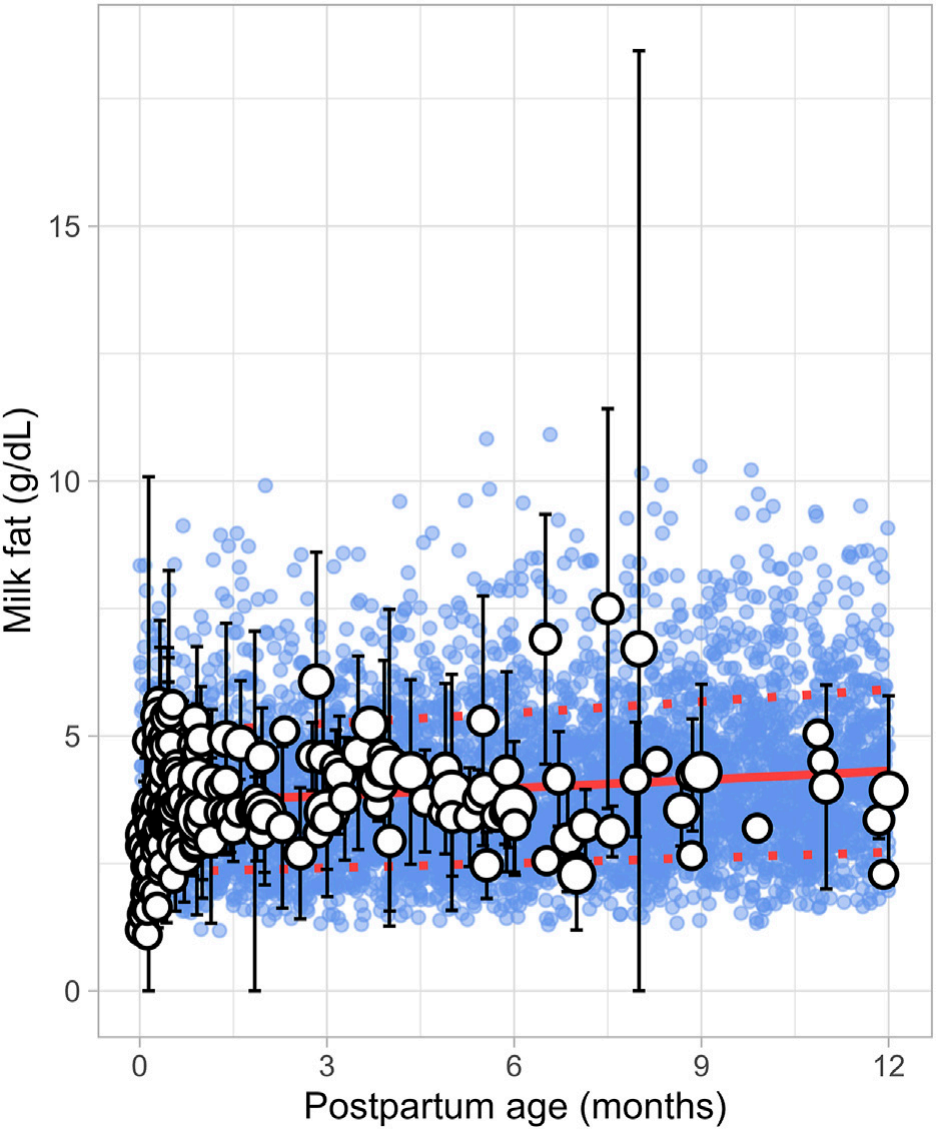
Simulated versus observed milk fat as a function of postpartum age. The blue dots depict simulated data points (n = 5,000) generated using the Simcyp Simulator V23R2, and the white dots represent the observed data. Error bars indicate standard deviations, and the dot sizes correspond to the number of samples. The solid red line is the simulated mean value, while the dotted red lines are the standard deviations.

A total of 43 publications related to milk fat content, also referred to as creamatocrit, were retrieved from the public domain. These studies included 5,012 data points from 2,661 mothers, with the postpartum period ranging from 1 day to 12 months (Supplementary Table 1). If the data were reported as creamatocrit, the Lucas equation was applied to convert these values to fat concentration in grams per deciliter (Lucas et al., 1978). To ensure the Lucas equation was suitable for analysis, the equation was first validated using an independent study from Meier et al. (2002). The collected data showed that milk fat concentration gradually increased from the early postpartum period and continued to rise over the first year of life. This trend was best described by a second-order polynomial equation (Equation 8) (Figure 4).

$$\text{Milk fat }\left(g/\text{dL}\right)=3.69\times \left(1+0.012083\times \text{PpT}+0.000171\times {\text{PpT}}^{2}\right)$$ (8)

Where PpT represents the postpartum age in months. To recover the observed variability, a constant CV of 37% was applied.

#### 3.2.4 Milk total protein

**FIGURE 5:**
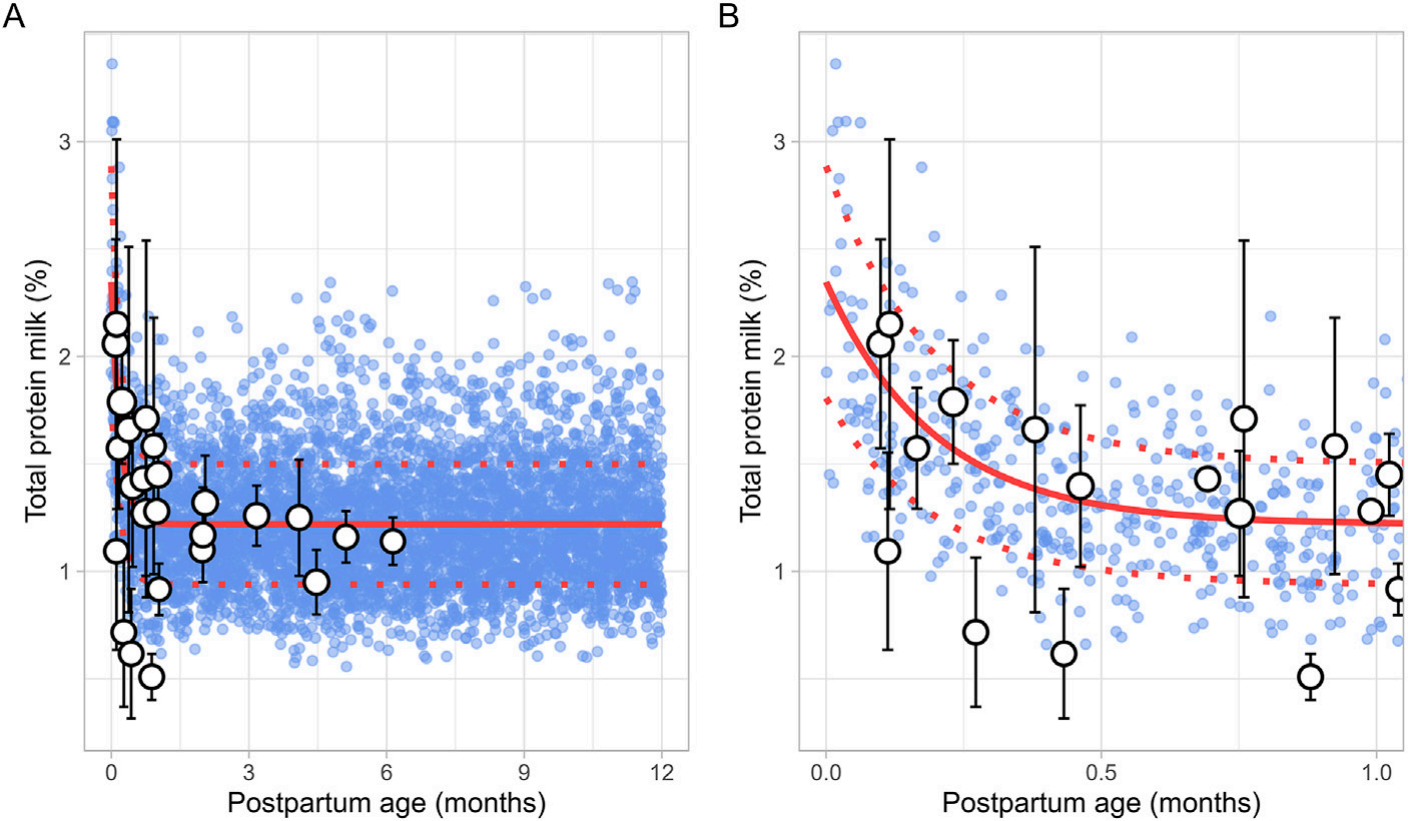
Simulated versus observed total milk protein as a function of postpartum age for **(A)** the entire age range and **(B)** for months 0 through 1. The blue dots represent simulated values (n = 5,000) generated using the Simcyp Simulator V23R2, and the white dots represent the observed data. Error bars indicate standard deviations, and the dot sizes correspond to the number of samples. The solid and dotted red lines are the mean and standard deviations of the simulated data.

The literature search identified 8 studies, including 961 data points from 481 postpartum women. The postpartum period for these women ranged from 3 days to approximately 6 months (Supplementary Table 1). Only fresh milk samples or those stored at 4°C for up to 24 h or at −20°C for up to 1 month were considered. Moreover, as previous studies have indicated that the protein content in preterm milk differs significantly from term milk, only data from full-term infants were included (Narang et al., 2006). In addition, only studies measuring total protein levels were selected for further evaluation. The analysis showed that total protein levels reached maximum values shortly after birth, followed by an exponential decline that stabilized around 1.5 months postpartum (Figure 5). This trend was best described using the following equation (Equation 9):

$$\text{Milk total protein }\left(\%\right)=1.219+{\;1.127\;e}^{\left(‐5.058\;\times \;\text{PpT}\right)}$$ (9)

Where PpT represents the postpartum age in months. To recover the observed variability, a constant CV of 23% was applied.

#### 3.2.5 Milk water content

**FIGURE 6:**
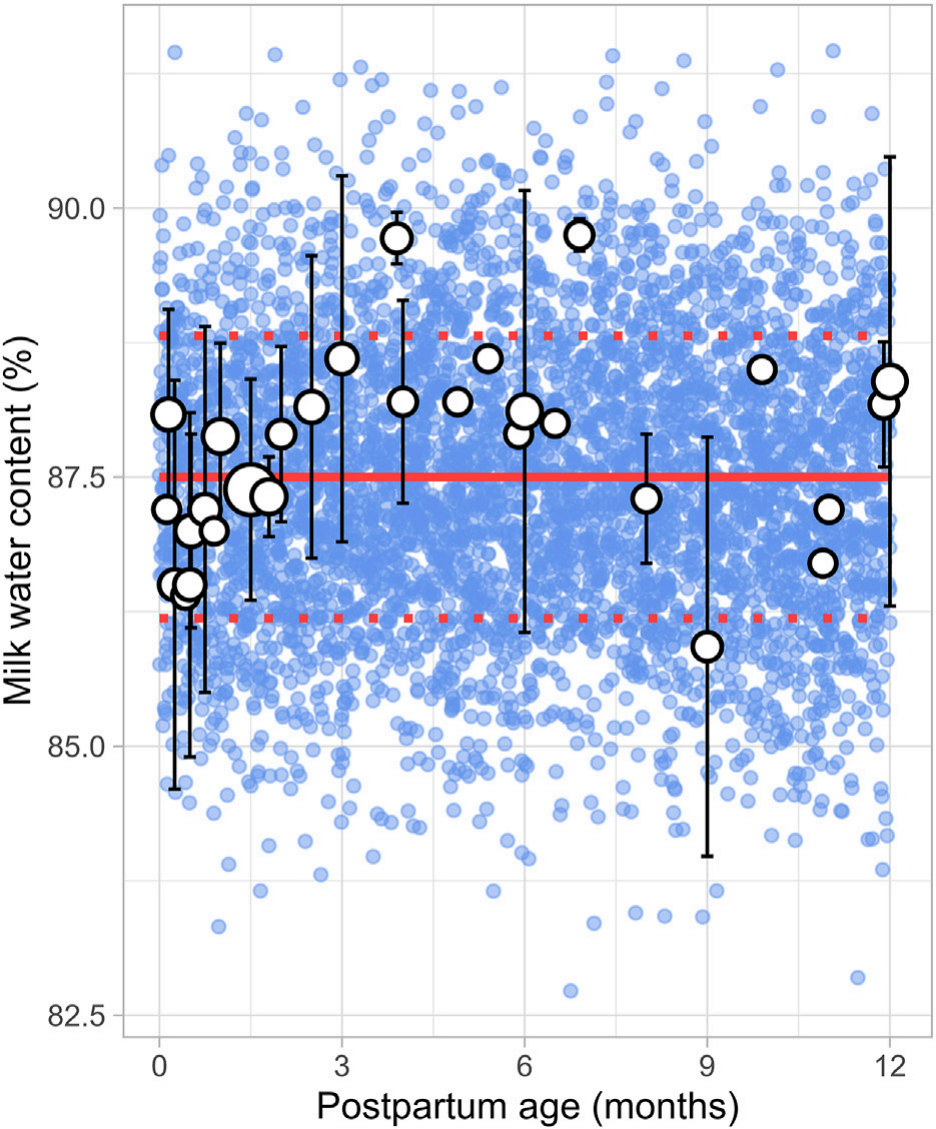
Simulated versus observed milk water content as a function of postpartum age. The blue dots depict simulated values (n = 5,000) generated using the Simcyp Simulator V23R2, and the white dots represent the observed data. Error bars indicate standard deviations, and the dot sizes correspond to the number of samples. The solid red line is the mean value of the simulated data, while the dotted red lines are the standard deviations.

A total of 8 studies were identified, providing 1,152 data points from 1,005 postpartum women, covering the period from 8 days to 12 months postpartum (Supplementary Table 1). Studies included in the analysis either directly measured milk water content (Christie et al., 1977; Butts et al., 2018; Bzikowska-Jura et al., 2020) or total solids/dry matter (Macy, 1949; Khan et al., 2013; Bzikowska-Jura et al., 2018; Czosnykowska-Lukacka et al., 2018; Huang and Hu, 2020). In cases where total solids or dry matter were measured, the water content was calculated as 100% minus the percentage of dry matter or total solids. The analysis showed that the water content in milk remained constant throughout the postpartum period, with a weighted mean value of 87.5% (Figure 6). In addition, a CV of 1.5% was required to account for the observed variability.

#### 3.2.6 Daily milk intake

**FIGURE 7:**
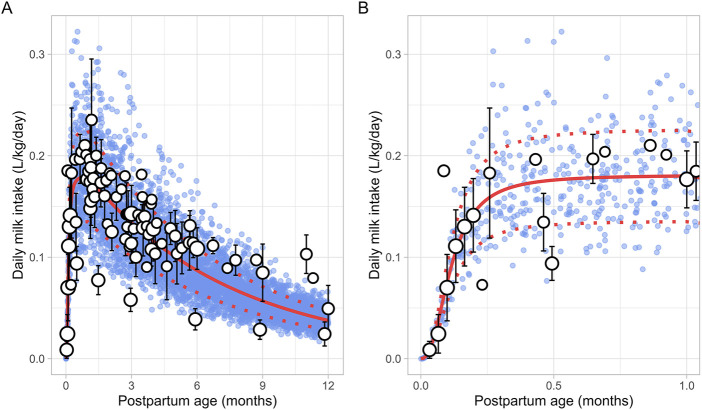
Simulated versus observed daily milk intake for **(A)** the entire age range and **(B)** for months 0 through 1. The blue dots depict simulated data points (n = 5,000) generated using the Simcyp Simulator V23R2, and the white dots represent the observed data. Error bars indicate standard deviations, and the dot sizes correspond to the number of samples. The solid and dotted red lines are the mean and standard deviations of the simulated data.

A total of 30 publications relevant to postpartum daily breast milk intake were retrieved, which included 164 data points from 2,417 infants (Supplementary Table 1). Only data from full-term infants who were exclusively breastfed were included in the analysis. For publications where infant weights were not reported, weights were estimated based on the child’s age and sex using the UK-WHO growth charts (RCPCH, 2013). If information on sex was unavailable, an average weight for both males and females at the reported postpartum age was used to adjust the daily milk intake volume. The data indicated that, following birth, the daily breast milk intake per kilogram of bodyweight (L/kg/day) rapidly increased during the first week of life, reaching maximum values between 0.5 and 1 month, after which it declined exponentially. Consequently, breast milk intake was best described using two functions, with the increase in milk consumption during the first month described using a sigmoidal equation (Equation 10) (Figure 7):

$$\text{Milk intake }\left(L/\text{kg}/\text{day}\right)=\frac{0.181\times {\text{PpT}}^{2.411}}{0.114^{2.411}+{\text{PpT}}^{2.411}}$$ (10)

The change in milk intake for 1 through 12 postpartum months was described using a mono-exponential function (Equation 11):

$$\text{Milk intake }\left(L/\text{kg}/\text{day}\right)=0.004+\left(0.208\;‐\;0.004\right)\;e^{\;\left(‐\;0.15\;\times \;\text{PpT}\right)}$$ (11)

PpT represents the months postpartum. A constant CV of 25% was needed to recover the variability in observed data.

### 3.3 Maternal haematocrit

**FIGURE 8:**
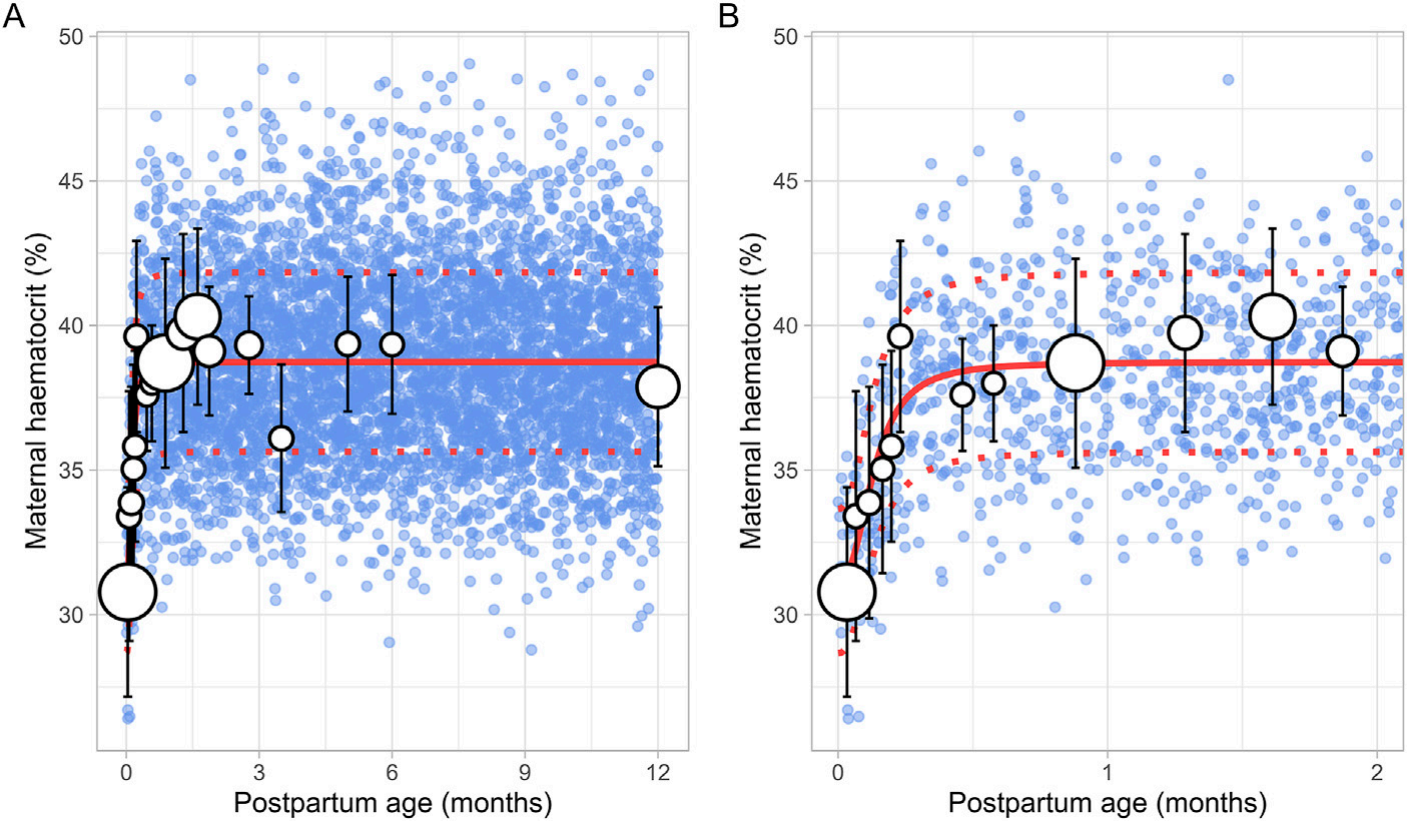
Simulated versus observed maternal haematocrit values for **(A)** the entire age range and **(B)** for 0 through 2 months postpartum. The blue dots represent simulated values (n = 5,000) that were generated using the Simcyp Simulator V23R2, and the white dots represent the observed data. Error bars indicate standard deviations, and the dot sizes correspond to the number of samples. The solid and dotted red lines are the mean and standard deviations of the simulated data. The data point at 12 months is a simulated data point obtained by simulating 1,000 healthy female volunteers using the Simcyp Simulator V23R2.

The literature search identified a total of 16 studies providing 20,186 data points from 11,004 women, with data collected from immediately after delivery up to 6 months postpartum (Supplementary Table 1). Only data from women with uncomplicated pregnancies were included. In addition, data beyond 6 months were not available. To address this, a simulated data point was generated by simulating 1,000 healthy female volunteers aged 18–45 years using the Simcyp Simulator (V23R2), representing haematocrit levels at the 12th postpartum month. The collected data indicated that the postpartum maternal haematocrit levels increased rapidly from approximately 31% at birth to pre-pregnancy levels of 40% within just 2 weeks after childbirth (Figure 8). After this rapid rise, haematocrit levels remained stable for the remainder of the postpartum period. The change in postpartum maternal haematocrit was best modelled using the following equation (Equation 12):

$$\text{Haematocrit }\left(\%\right)=31.17+\frac{\left(38.74\;‐\;31.17\right)\times {\text{PpT}}^{2.49}}{0.133^{2.49}+{\text{PpT}}^{2.49}}$$ (12)

Where PpT represents postpartum age in months. A CV of 8% was incorporated to account for the observed variability in haematocrit.

### 3.4 Maternal alpha-1-acid glycoprotein (m-AGP)

**FIGURE 9:**
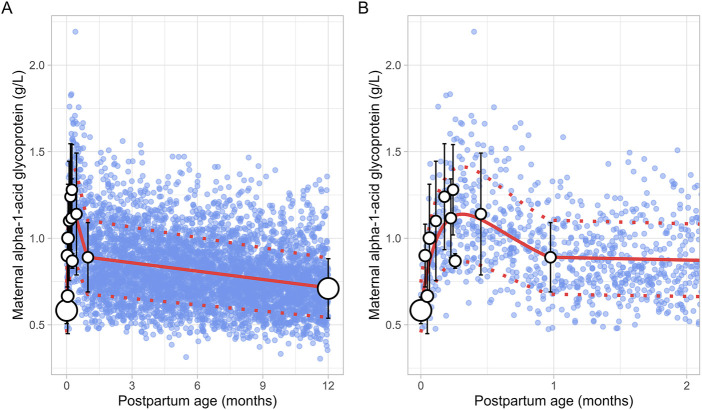
Comparison of simulated and observed maternal alpha-1-acid glycoprotein values for **(A)** the entire age range and **(B)** for 0 through 2 months postpartum. The blue dots represent simulated values (n = 5,000) generated using the Simcyp Simulator V23R2, and the white dots depict the observed data. Error bars indicate standard deviations, and the dot sizes correspond to the number of samples. The solid red line is the mean value of the simulated, while the dotted red lines are the standard deviations. The data point at 12 months is a simulated data point obtained by simulating 1,000 healthy female volunteers using the Simcyp Simulator V23R2.

The literature search retrieved a total of 6 studies that provided 145 data points on postpartum m-AGP levels from 88 women, with data collected immediately after delivery up to 1 month postpartum (Supplementary Table 1). Due to the absence of data after the first month postpartum, an additional simulated data point was generated by simulating 1,000 healthy female volunteers aged 18–45 years using the Simcyp Simulator (V23R2). This simulated data point was assumed to represent 12 months postpartum. The collected data showed that plasma m-AGP levels rose sharply after birth, peaking during the first week postpartum, and then decreased by 30% by the end of the fourth week. After the first month postpartum, m-AGP levels were assumed to decline linearly due to lack of data, returning to pre-pregnancy levels by 12 months postpartum (Figure 9). The tested models were not able to capture the data trend, hence these data were modelled using two different functions. For the period up to 1 month postpartum, a double Weibull equation best described the data (Equation 13):

$${m‐\text{AGP}\;\left(g/L\right)=e}^{\left(‐1.277\;\times \;\text{PpT}\right)\;}‐\;e^{\left(‐\;6.749\;\times \;\text{PpT}\right)}+0.6$$ (13)

For the period from 1 to 12 months postpartum, m-AGP levels were assumed to decrease linearly using the following linear regression equation (Equation 14):

$$m‐\text{AGP }\left(g/L\right)=0.016\times \text{PpT}+0.90$$ (14)

Where PpT represents postpartum age in months in both equations. A constant CV of 24% was incorporated to account for the observed variability in m-AGP.

### 3.5 Maternal human serum albumin (m-HSA)

A total of 13 studies were retrieved that included 1,757 observations on postpartum m-HSA levels from 1,680 women, with data obtained from delivery up to about 6 months postpartum (Supplementary Table 1). Due to the absence of data beyond 6 months, an additional data point was generated by simulating 1,000 healthy female volunteers aged 18–45 years using the Simcyp Simulator (V23R2), representing m-HSA levels at 12 months postpartum.

**FIGURE 10:**
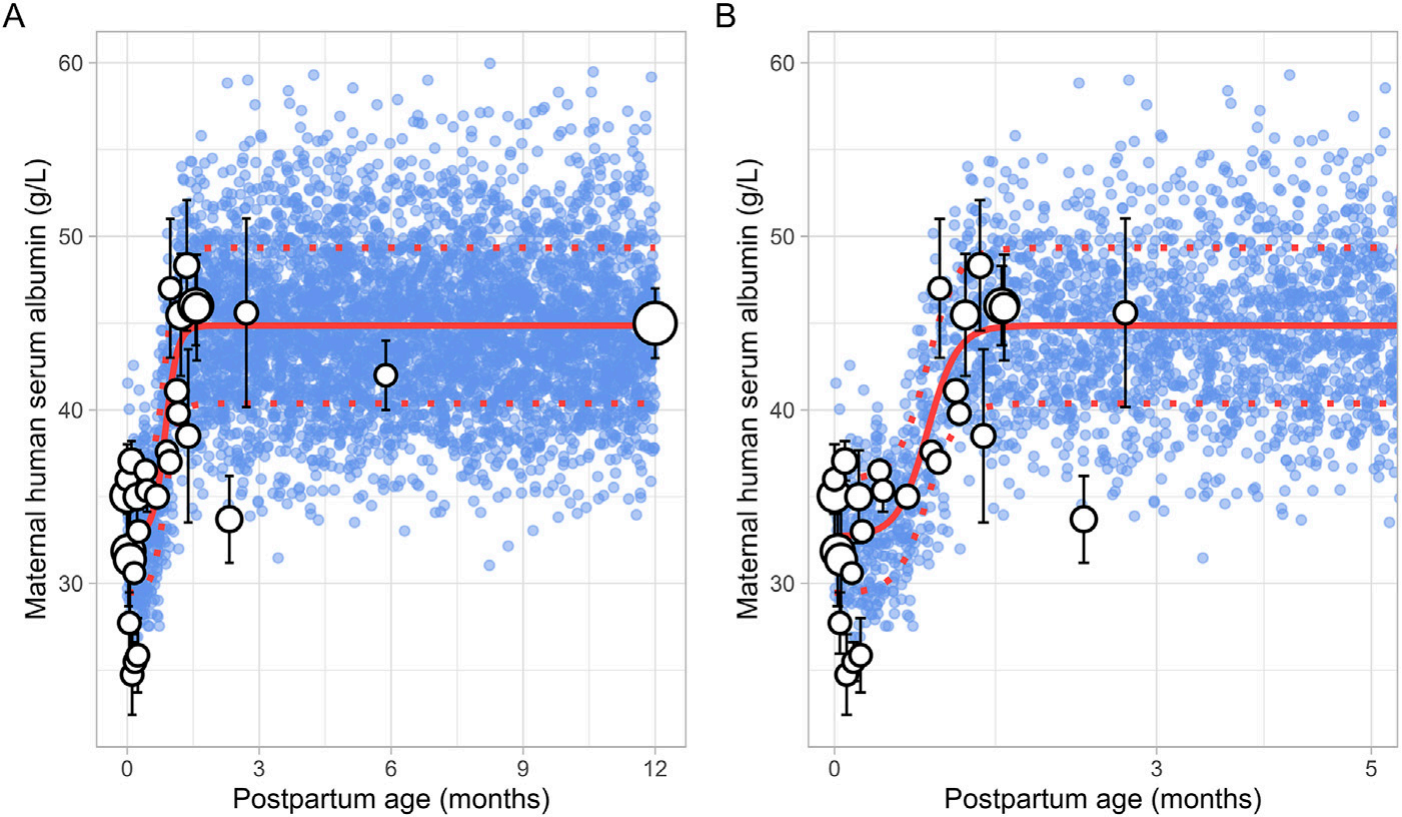
Simulated versus observed maternal human serum albumin levels for **(A)** the entire age range and **(B)** for 0 through 5 months postpartum. The blue dots represent the simulated values (n = 5,000) generated using the Simcyp Simulator V23R2, and the white dots represent the observed data. Error bars indicate standard deviations, and the dot sizes correspond to the number of samples. The solid and dotted red lines are the mean and standard deviations of the simulated data. The data point at 12 months is a simulated data point obtained by simulating 1,000 healthy female volunteers using the Simcyp Simulator V23R2.

The meta-analysis showed that m-HSA levels remained stable during the first 2 weeks postpartum, although considerable variability was observed in the collected data. After this period, m-HSA levels gradually increased, reaching 47 ± 4.00 g/L (mean ± SD) by the first month postpartum, comparable to pre-pregnancy levels (Figure 10). Following this recovery phase, substantial variability was observed in the collected data. For instance, m-HSA levels were recorded at 45.89 ± 3.05 g/L at 1.58 months postpartum, followed by a significant decline to 33.70 ± 2.50 g/L at 2.32 months postpartum, and then an increase to 45.60 ± 5.43 g/L at 2.71 months postpartum. Despite this variability, the overall trend indicated that after approximately 1 month postpartum, when pre-pregnancy levels were restored, m-HSA levels remained stable up to 12 months postpartum. The equation that best describes the initial increase and subsequent stabilization of m-HSA levels is as follows (Equation 15):

$$m‐\text{HSA }\left(g/L\right)=32.7+\left(\frac{12.15}{1+e^{\left(‐7.16\;\times \;\left(\text{PpT}\;‐\;0.866\right)\right)}}\right)$$ (15)

Where PpT represents postpartum age in months. A constant CV of 10% was incorporated to account for the observed variability in m-HSA. Figure 10 provides a comparison between the predicted and observed m-HSA values.

### 3.6 Breast volume

**FIGURE 11:**
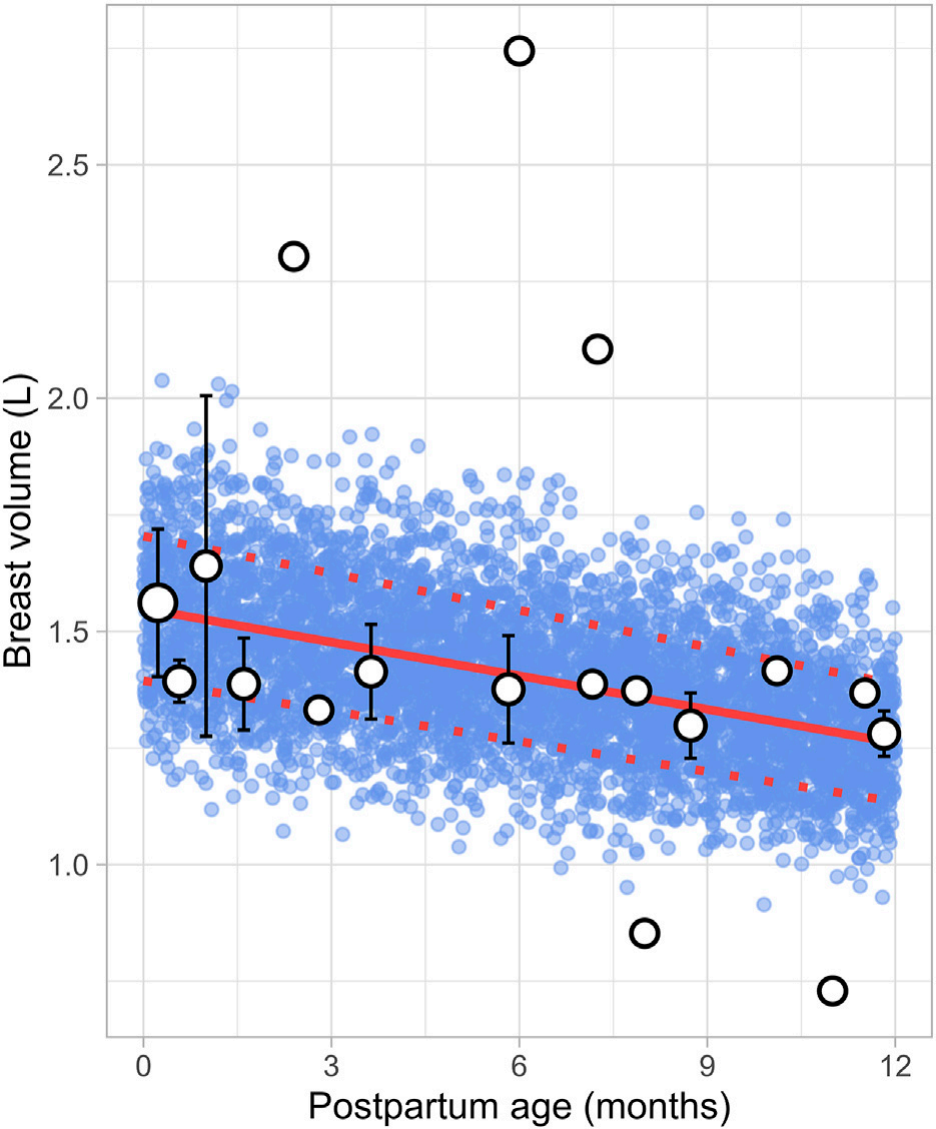
Simulated versus observed breast volume values. The blue dots represent simulated data points (n = 5,000) generated using the Simcyp Simulator V23R2, and the white dots depict the observed data. Error bars indicate standard deviations, and the dot sizes correspond to the number of samples. The solid red line is the mean value of the simulated data, whereas the dotted red lines are the standard deviations.

Six studies that measured the breast volume in postpartum women were included for further analysis. These studies comprised a total of 226 data points from 191 women, covering the period from 1 week to 12 months postpartum (Supplementary Table 1). Since total breast volume includes the combined volumes of glandular tissue, adipose tissue, and milk, to estimate actual breast tissue volume (also called empty breast volume), estimated milk production was subtracted from the total volume. The data showed that empty breast volume gradually decreased over time, with values of 1.56 ± 0.16 L (mean ± SD) at 0.23 months, 1.41 ± 0.10 L at 3.63 months, 1.38 ± 0.12 L at 5.83 months, and 1.28 ± 0.05 L at 11.82 months postpartum. Figure 11 shows that the overall trend is best characterized by a gradual decline over time, with the following linear regression equation providing the best fit to the data (Equation 16):

$$\text{Breast volume }\left(L\right)=1.549\;‐\;0.024\times \text{PpT}$$ (16)

Where PpT represents the postpartum age in months, a constant CV of 10% was added to account for the observed variability in breast volume.

### 3.7 Maternal plasma volume

**FIGURE 12:**
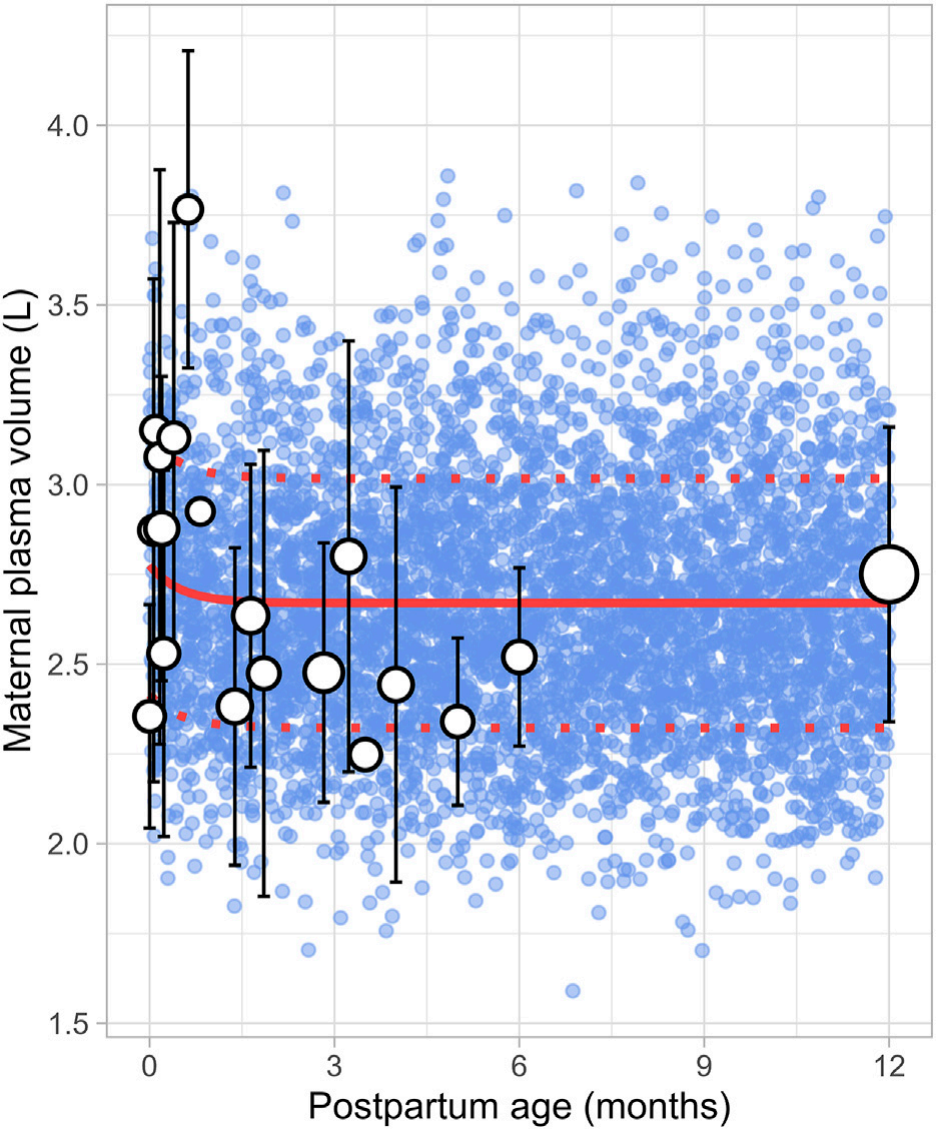
Simulated versus observed maternal plasma volume values. The blue dots represent simulated individual values (n = 5,000) generated using the Simcyp Simulator V23R2, and the white dots represent the observed data. Error bars indicate standard deviations, and the dot sizes correspond to the number of samples from each study. The solid and dotted red lines are the mean and standard deviations of the simulated data. The data point at 12 months is a simulated data point obtained by simulating 1,000 healthy female volunteers using the Simcyp Simulator V23R2.

The literature review identified 22 studies, contributing 651 observations on postpartum plasma volume from 560 women, with data ranging from immediately after delivery to 6 months postpartum (Supplementary Table 1). Since the type of delivery was not consistently reported across studies, no distinction was made between vaginal and caesarean deliveries. In addition, data on plasma volume from lactating women after 6 months were not available. A simulated data point was therefore generated by simulating 1,000 healthy female volunteers aged 18–45 years using the Simcyp Simulator (V23R2). This simulated data point was assumed to represent the mean plasma volume at 12 months postpartum (Figure 12). The data collected from the different studies showed significant variability, with plasma volume values ranging from 2.35 ± 0.31 L (mean ± SD) at birth to 3.08 ± 0.80 L at 0.71 weeks postpartum, followed by a decrease to 2.53 ± 0.51 L at 1 week postpartum. The meta-analysis indicated that after the plasma volume expansion during pregnancy (Abduljalil et al., 2012), maternal plasma volume dropped immediately after birth likely due to blood loss, then continued to gradually decrease, returning to pre-pregnancy levels by the second month postpartum. The following equation (Equation 17) best described the observed changes in plasma volume:

$$\text{Plasma volume }\left(L\right)=2.67+0.106\times \left(0.133^{\text{PpT}}\right)$$ (17)

Where PpT represents postpartum age in months. A constant CV of 13% was included to account for the observed variability in plasma volume.

### 3.8 Maternal cardiac output

A total of 47 clinical studies that reported changes in postpartum maternal cardiac output were included in this analysis, encompassing 2,616 observations from 1,542 women. Data were available from 1 day to 12 months postpartum, although no data were reported for the period between 7 and 12 months postpartum (Supplementary Table 1). The cardiac output decreased from 402.25 ± 36.78 L/h (mean ± SD) immediately after delivery to pre-pregnancy levels of 302.42 ± 65.80 L/h by the end of the fourth postpartum week. The steepest decline occurred at 2 weeks postpartum, where cardiac output dropped to 308.15 ± 20.09 L/h, representing a 23% decrease. After this point, cardiac output remained relatively stable, with a value of 311.40 ± 69.01 L/h observed at 12 months postpartum. The longitudinal change in cardiac output during the postpartum period was modelled using the following equation (Equation 18):

$$\text{Cardiac output }\left(L/h\right)=98.8\;e^{\left(‐3.33\;\times \;\text{PpT}\right)}+304.4\;e^{\left(‐0.00096\;\times \;\text{PpT}\right)}$$ (18)

**FIGURE 13:**
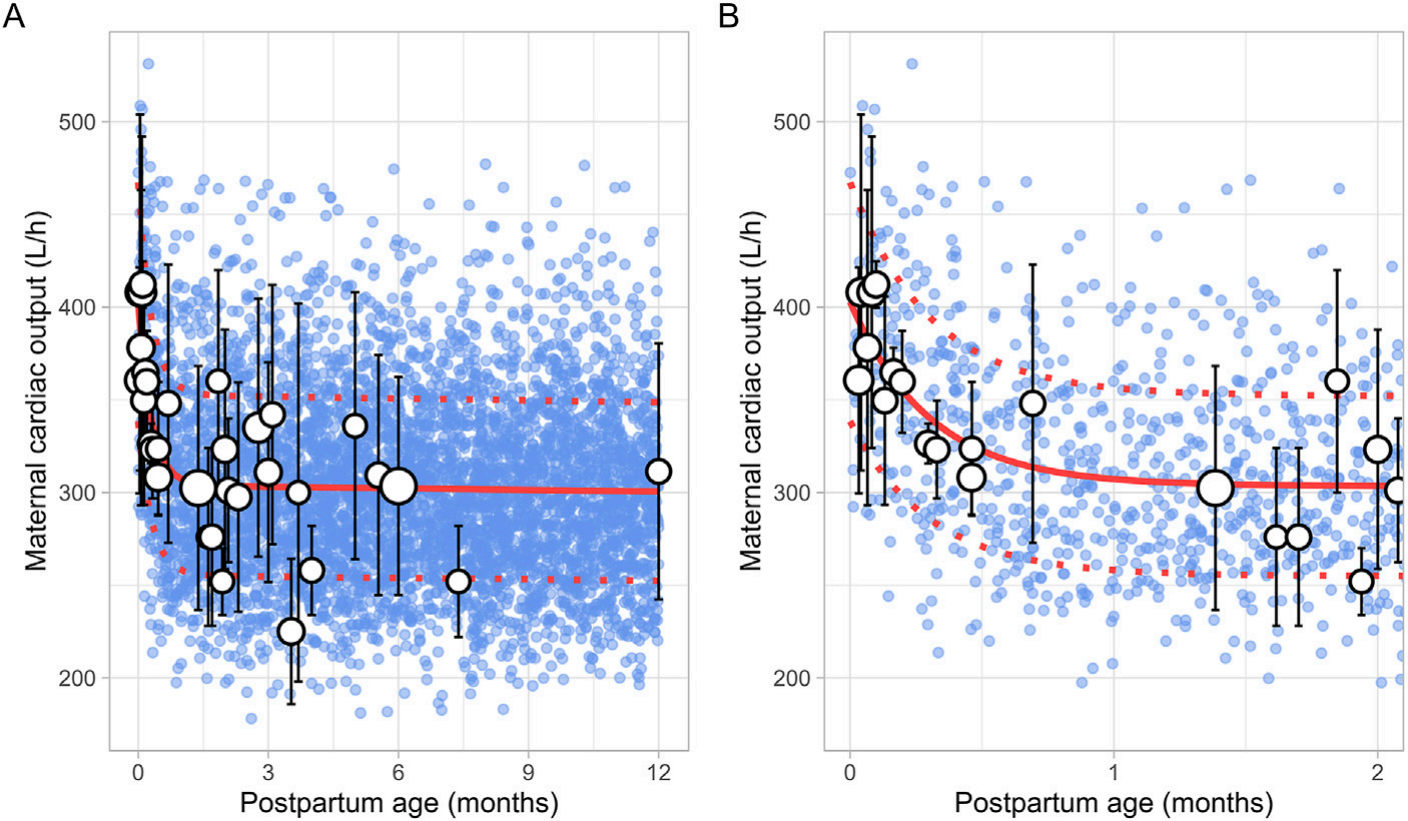
Comparison of simulated and observed maternal cardiac output values **(A)** over the entire age range and **(B)** from 0 to 2 months postpartum. Blue dots represent simulated values (n = 5,000) generated using the Simcyp Simulator V23R2, while white dots show the observed mean data. The size of the dots depicts the number of samples, and error bars represent the standard deviations. The solid red line indicates the average of the simulated data, whereas dotted red lines represent the standard deviations.

Where PpT represents the postpartum age in months, a constant CV of 16% was added to account for the observed variability in cardiac output. Figure 13 provides a comparison between the predicted and observed cardiac output values.

### 3.9 Maternal renal function

**FIGURE 14:**
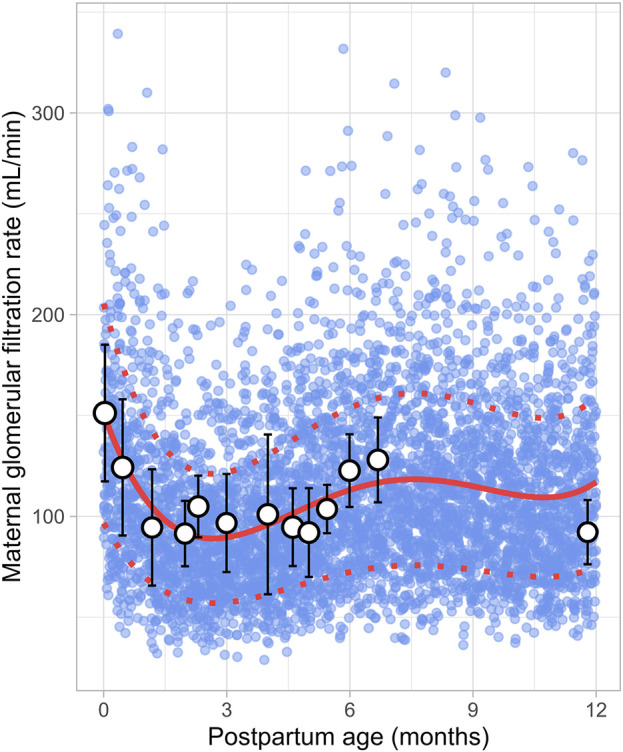
Simulated versus observed maternal glomerular filtration rate values. The blue dots represent simulate data points (n = 5,000) generated using the Simcyp Simulator V23R2, and the white dots represent the observed data. Error bars indicate standard deviations, and the dot sizes correspond to the number of samples. The solid red line is the mean value of the simulated data, while the dotted red lines are the standard deviations.

Data on postpartum changes in maternal GFR (m-GFR) were retrieved from 10 clinical studies comprising 165 observations from 165 women. The postpartum period studied ranged from 1 day to 12 months, though no data were available for the period between 7 and 11 months postpartum (Figure 14). The studies included in this analysis are listed in Supplementary Table 1. The data showed that m-GFR decreased from an immediate postpartum value of 152 ± 34 mL/min (mean ± SD) to the lowest value observed at 2 months postpartum, where m-GFR dropped to 92 ± 15 mL/min. After 2 months postpartum, m-GFR showed a substantial increase, gradually rising to 128 ± 23 mL/min, eventually returning to non-pregnant levels by approximately 5 months postpartum. To model the longitudinal changes in m-GFR during the postpartum period, the following equation (Equation 19) was used:

$$\text{GFR }\left(\text{mL}/\min\right)=151.0285\;‐\;57.1898\times \text{PpT}+17.1856\times {\text{PpT}}^{2}\;‐\;1.8479\times {\text{PpT}}^{3}+0.0661\times {\text{PpT}}^{4}$$ (19)

Where PpT represents the postpartum age in months. A constant CV of 36% was applied to account for the observed variability in the data.

## 4 Discussion

In recent years, several lactation PBPK models have been developed to predict drug transfer into human breast milk (Abduljalil et al., 2021; Pansari et al., 2022; Nauwelaerts et al., 2023). While these models have been useful, many assume that maternal physiology and milk composition remain constant throughout the postpartum period. However, our analysis revealed that physiological changes after pregnancy return to pre-pregnancy levels at different times and at varying rates. For instance, breast volume, maternal plasma volume, maternal cardiac output and m-GFR decrease over the course of lactation (Figures 11–14). In contrast, maternal haematocrit, m-AGP and m-HSA levels increase in function of postpartum age (Figures 8–10). Additionally, the body undergoes further adaptations to support breastfeeding, leading to postpartum age-dependent changes in milk production, pH, fat and protein content (Figures 2–5). As these dynamic processes individually and collectively affect maternal and milk drug exposure, incorporating them into lactation PK models could improve model predictions. Mathematical functions that describe changes in maternal physiology and milk composition during the postpartum period were, therefore, developed in this study. These functions can be integrated into lactation PK models, allowing for a more realistic description of the postpartum population.

Dallmann et al. previously compiled a database incorporating changes in maternal physiology throughout the postpartum period (Dallmann et al., 2020). However, they did not investigate variations in breast milk composition, which can significantly affect drug transfer into breast milk. The current study expands on earlier works by including additional data and investigating changes in both maternal physiological and breast milk composition parameters to generate a realistic lactation population.

Human breast milk is composed of approximately 88% water, 7% carbohydrates, 1% protein, and 4% fat (Kunz et al., 1999). However, its composition is not fixed; it changes throughout the postpartum period to meet the nutritional requirements of the baby (Ballard and Morrow, 2013). For instance, in the first few days after birth, the mother produces low quantities of colostrum, a thick yellow fluid that is low in fat but high in protein. Between 7 and 14 days postpartum, transitional milk is produced, which is a mixture of colostrum and mature milk. After approximately 14 days, mature milk is produced, which has a higher fat and lower protein content than colostrum. This change in fat and protein content over time is evident in our meta-analysis, which showed an increase in milk fat and a decrease in protein levels in function of postpartum age (Figures 4, 5). Another important characteristic of milk is its pH, which is more acidic than plasma, with an average value of around 7.2 for mature milk (Abduljalil et al., 2021). Like other milk components, the pH of milk varies significantly with postpartum age. Our analysis indicated that colostrum has a substantially higher pH than mature milk (Figure 3). This difference in pH can significantly impact the behaviour of predominantly basic drugs, which become more ionised in the acidic environment of mature milk compared to colostrum, leading to more significant accumulation in mature milk through a process known as ion trapping. Indeed, it has been demonstrated previously that a lactation PBPK model for the antidepressant fluoxetine could only match the observed milk-to-plasma ratios in both colostrum and mature milk if the milk pH was adjusted to the measured pH values (Pansari et al., 2022). Furthermore, our analysis showed that maternal milk production increased rapidly in the first few weeks postpartum, stabilised for about 6 months, and then declined exponentially (Figure 2). Likewise, the daily milk intake rose sharply in early postpartum, reaching its peak at around 1 month, in line with previously conducted analyses (Figure 7; Supplementary Figure 1) (Yeung et al., 2020; Rios-Leyvraz and Yao, 2023). Moreover, previous studies have shown a positive relationship between nursing frequency and milk production in the early postpartum period, explaining why milk volume peaks within the first 14 days (De Carvalho et al., 1983; Hopkinson et al., 1988). However, the data collected in this study showed no clear relationship between daily milk intake and milk volume during the later stages of lactation. While daily milk intake gradually decreased after peaking around the first month postpartum, milk volume remained constant for up to 6 months postpartum. This observation is supported by Kent et al., who found no correlation between the number of daily breastfeeding sessions and 24-h milk production from 1 to 6 months postpartum (Kent et al., 2006). After 6 months postpartum, a significant decline in milk volume was observed, most likely due to the introduction of complementary foods. However, the timing and quantity of complementary food introduction, which can influence milk production, were not consistently provided in the available studies and could therefore not be included as a covariate in the analysis. Moreover, the current meta-analysis showed that breast volume decreased linearly in function of postpartum age (Figure 11). This decline likely results from redistribution of breast tissue and/or loss of mammary cells. Indeed, Hartmann et al. observed significant mobilization of fat during lactation, while Quarrie et al. showed that mammary cell apoptosis is a normal part of the lactating and involuting mammary gland (Hartmann et al., 1995; Quarrie et al., 1996). Despite the observed reduction in breast volume, milk volume remained stable for up to 6 months postpartum. This suggests that while initial proliferation of breast tissue is essential for milk production, the breast does not need to remain enlarged to maintain its milk production capacity.

The current meta-analysis indicated that the elevated plasma volume observed during pregnancy gradually decreased throughout the postpartum period, eventually returning to pre-pregnancy levels within 2 months (Figure 12). At the same time, as plasma volume declines, red blood cells become more concentrated, leading to a rise in haematocrit levels, as observed in our analysis (Figure 8). Moreover, the reduction in plasma volume also raises the oncotic pressure, resulting in a decrease in absolute m-GFR values in the early postpartum. Following this initial decline in m-GFR, a gradual increase was observed, and pre-pregnancy values were restored after approximately 5 months (Figure 14). This is in line with observations of Harel et al. who showed that postpartum serum creatinine concentrations return to pre-pregnancy levels by 18 weeks postpartum (Harel et al., 2019).

The collected data for early postpartum plasma volume showed significant variability, which may be due to the fact that these observations were not stratified by the type of delivery. Indeed, studies have shown that blood loss is significantly higher in caesarean sections compared to vaginal deliveries (Larsson et al., 2006; Stafford et al., 2008). This likely affects plasma volume measurements during the early postpartum period thus contributing to the observed variability. Substantial variability was also observed in cardiac output measurements during early postpartum, with values on the first postpartum day ranging from 300 ± 56.05 L/h (Ambrožič et al., 2020) to 429 ± 29.58 L/h (Robson et al., 1989). This variability may originate from parity-specific differences in maternal cardiac adaptation during pregnancy, as studies have shown that parous women tend to have higher cardiac output than nulliparous women (Ling et al., 2019). These differences could persist throughout the postpartum period, possibly explaining the wide range of values observed. Moreover, in the first 48 h after birth, some studies reported an increase in cardiac output of up to 20%, while others observed a 10% decrease (Robson et al., 1987a; Ram et al., 2017; Lavie et al., 2018; Timokhina et al., 2019). This variation likely results from differences in blood loss during delivery, as well as individual factors such as body weight. At 2 weeks after delivery, the cardiac output data became more consistent, showing a substantial decrease in cardiac output by 13%–27% relative to the end of pregnancy (Robson et al., 1987a; Robson et al., 1987b; Robson et al., 1989; Hunter and Robson, 1992). This sharp change in cardiac output during the early postpartum period is well described by the double exponential function we derived (Figure 13).

Plasma protein levels decrease by about 20% by the end of pregnancy compared to pre-pregnancy levels (Abduljalil et al., 2012). After birth, plasma levels of m-AGP and m-HSA rise quickly, returning to pre-pregnancy levels by around 1 month postpartum (Figures 9, 10). In addition, our analysis showed that m-AGP levels exceed pre-pregnancy values in the early postpartum period, reaching maximum values (1.28 ± 0.26 g/L) approximately 1 week after delivery. Since AGP is an acute-phase reactant protein that increases in response to physiological stress, this rise likely reflects the body’s reaction to the stress of childbirth. Indeed, the increase in m-AGP levels following childbirth has been previously demonstrated by Larijani et al., who showed that m-AGP continues to rise for at least 3 days following vaginal or caesarean delivery (Larijani et al., 1990).

Although the derived functions accurately described the observed data, several challenges were encountered during data collection and analysis. One major challenge was the fact that some studies pooled data from a wide range of postpartum ages and reported only mean values, which could lead to distorted results. Moreover, many studies presented their data graphically, thereby making data extraction subject to technical errors. Another issue was that no data was available at 12 months postpartum for some parameters. To address this matter, simulated mean values from 1,000 virtual healthy female volunteers were generated using the Simcyp Simulator. Although this virtual population was developed and validated using substantial observed clinical data, this method could introduce bias as it assumes that these specific parameters return to their pre-pregnancy levels by 12 months postpartum. Another challenge that we encountered during the analysis was the inconsistent reporting of specific covariates, such as delivery type and parity. As noted earlier, these factors are known to influence certain parameters and accounting for them could further reduce variability and improve the derived functions. Additionally, we could not distinguish between hindmilk and foremilk in our analysis, despite the known difference in fat content, with hindmilk being fattier (Takumi et al., 2022). Attempts to stratify the data based on collection timing (foremilk versus hindmilk) resulted in insufficient data. Similarly, we could not differentiate between samples collected after complete or partial milk emptying, as this information was often missing from the publications. Furthermore, although this article provides a substantial collection of data, it does not encompass all relevant parameters. For example, the activity of various metabolizing enzymes and transporters changes considerably throughout pregnancy (Abduljalil et al., 2020; Pinheiro and Stika, 2020; Chen et al., 2024). These activities are expected to return to pre-pregnancy levels during the postpartum period, which could have a significant impact on drug metabolism and distribution. However, reliable data on the time course of these enzyme and transporter activities returning to their pre-pregnancy levels are still missing and should, therefore, be the focus of future studies to achieve a more complete description of the postpartum population.

In summary, the current study provides a comprehensive and up-to-date database of maternal physiological and milk composition parameters throughout the postpartum period. In addition, this dataset presents a robust basis for developing lactation PBPK models that account for these dynamic changes over time. Such models are valuable tools for investigating and optimizing drug dosing regimens in the postpartum population.

## Data Availability

The original contributions presented in the study are included in the article/Supplementary Material, further inquiries can be directed to the corresponding author.
