# Supplementary material for: Postpartum changes in maternal physiology and milk composition: a comprehensive database for developing lactation physiologically-based pharmacokinetic models
Source: Front Pharmacol. 2025 Feb 3;16:1517069. doi: 10.3389/fphar.2025.1517069 (PMC11830814; doi:10.3389/fphar.2025.1517069)
Supplement: Supplementary file 1 [file DataSheet1.docx]

Supplementary Material

# Supplementary Tables

**Supplementary Table 1.** Values and equations for maternal physiology and milk composition parameters, derived from a meta-analysis of published data.

| **Parameter (unit)** | **Value^a^** | **Equation** | **References** |
| --- | --- | --- | --- |
| Milk fat (g/dL) | 0.03 months: 1.20  [n = 9] | $\text{Milk fat} \left( \text{g/dL} \right)\text{ = 3.69 × (1 + 0.012083 × PpT + 0.000171 ×} \text{PpT}^{\text{2}}\text{)}$ | (Luisa, 1995) |
|  | 0.06 months: 1.50  [n = 9] |  | (Luisa, 1995) |
|  | 0.08 months: 2.84 ± 1.30 (45 %)  [n = 69] |  | (Corvaglia et al., 2008) |
|  | 0.093 months: 1.64 ± 0.15 (9 %)  [n = 3] |  | (Anderson et al., 1981) |
|  | 0.096 months: 1.90  [n = 1] |  | (Anderson et al., 1981) |
|  | 0.097 months: 3.06 ± 1.5 (49 %)  [n = 123] |  | Anderson et al., 1983; Najman et al., 2012; Sever et al., 2015 ; (Luisa, 1995) |
|  | 0.098 months: 2.45  [n = 1] |  | (Anderson et al., 1981) |
|  | 0.099 months: 2.80 ± 0.11 (40 %)  [n = 69] |  | Najman et al., 2012; Gross et al., 1980 |
|  | 0.120 months: 1.10  [n = 1] |  | (Anderson et al., 1981) |
|  | 0.122 months: 1.58  [n = 1] |  | (Anderson et al., 1981) |
|  | 0.123 months: 2.72  [n = 1] |  | (Anderson et al., 1981) |
|  | 0.125 months: 2.07 ± 0.44 (21 %)  [n = 3] |  | (Anderson et al., 1981) |
|  | 0.126 months: 3.56 ± 0.67 (19 %)  [n = 4] |  | (Anderson et al., 1981) |
|  | 0.128 months: 3.38  [n = 1] |  | (Anderson et al., 1981) |
|  | 0.129 months: 2.66 ± 0.28 (10 %)  [n = 12] |  | (Anderson et al., 1981; Luisa, 1995) |
|  | 0.14 months: 4.90 ± 5.18 (106 %)  [n = 6] |  | (Hytten, 1994) |
|  | 0.159 months: 3.72  [n = 1] |  | (Anderson et al., 1981) |
|  | 0.161 months: 2.40  [n = 9] |  | (Luisa, 1995) |
|  | 0.162 months: 2.42  [n = 1] |  | (Anderson et al., 1981) |
|  | 0.17 months: 1.98  [n = 1] |  | Anderson et al., 1981 |
|  | 0.226 months: 3.10 ± 1.00 (32 %)  [n = 9] |  | (Anderson et al., 1981) |
|  | 0.230 months: 3.11 ± 0.94 (30 %)  [n = 48] |  | (Saarela et al., 2005) |
|  | 0.231 months: 3.24 ± 1.03 (32 %)  [n = 79] |  | (Gross et al., 1980; Lemons et al., 1982; Saarela et al., 2005) |
|  | 0.233 months: 2.98 ± 0.30 (10 %)  [n = 7] |  | (Lemons et al., 1982) |
|  | 0.252 months: 1.91  [n = 1] |  | (Anderson et al., 1981) |
|  | 0.255 months: 2.75  [n = 1] |  | (Anderson et al., 1981) |
|  | 0.261 months: 3.69 ± 0.22 (6 %)  [n = 3] |  | (Anderson et al., 1981) |
|  | 0.269 months: 3.02  [n = 1] |  | (Anderson et al., 1981) |
|  | 0.280 months: 4.09  [n = 1] |  | (Maas et al., 1998) |
|  | 0.282 months: 1.64  [n = 1] |  | (Anderson et al., 1981) |
|  | 0.286 months: 3.03 ± 1.80 (59 %)  [n = 6] |  | (Hytten, 1994) |
|  | 0.291 months: 4.84 ± 0.93 (19 %)  [n = 3] |  | (Anderson et al., 1981) |
|  | 0.292 months: 3.13 ± 0.62 (20 %)  [n = 2] |  | (Anderson et al., 1981) |
|  | 0.294 months: 4.71 ± 0.20 (4 %)  [n = 3] |  | (Anderson et al., 1981) |
|  | 0.295 months: 3.19 ± 0.05 (2 %)  [n = 2] |  | (Anderson et al., 1981) |
|  | 0.296 months: 5.68  [n = 1] |  | (Anderson et al., 1981) |
|  | 0.322 months: 5.39 ± 1.88 (35 %)  [n = 78] |  | (Kociszewska-Najman et al., 2012) |
|  | 0.327 months: 2.85 ± 0.21 (7 %)  [n = 2] |  | (Anderson et al., 1981) |
|  | 0.329 months: 2.36 ± 1.65 (32 %)  [n = 45] |  | (Anderson et al., 1981; Kociszewska-Najman et al., 2012) |
|  | 0.332 months: 2.48  [n = 1] |  | (Anderson et al., 1981) |
|  | 0.354 months: 4.01  [n = 1] |  | (Anderson et al., 1981) |
|  | 0.357 months: 3.63  [n = 1] |  | (Anderson et al., 1981) |
|  | 0.359 months: 3.54  [n = 1] |  | (Anderson et al., 1981) |
|  | 0.412 months: 3.29 ± 0.80 (22 %)  [n = 31] |  | (Maas et al., 1998) |
|  | 0.43 months: 4.93 ± 1.95 (59 %)  [n = 72] |  | (Gross et al., 1980; Hytten, 1994) |
|  | 0.45 months: 4.35 ± 1.81 (37 %)  [n = 140] |  | (Anderson et al., 1981; Luisa, 1995; Kociszewska-Najman et al., 2012; Sever et al., 2015) |
|  | 0.461 months: 4.79 ± 1.71 (40 %)  [n = 173] |  | (Clark et al., 1982; Goldfarb and Savadove, 1991; Kociszewska-Najman et al., 2012) |
|  | 0.462 months: 5.37 ± 1.75 (37 %)  [n = 83] |  | (Gross et al., 1980; Lemons et al., 1982; Lubetzky et al., 2007) |
|  | 0.47 months: 5.44 ± 2.88 (53 %)  [n = 68] |  | (Lemons et al., 1982; Vieira et al., 2004) |
|  | 0.488 months: 3.63  [n = 1] |  | (Anderson et al., 1981) |
|  | 0.491 months: 4.85 ± 0.09 (3 %)  [n = 2] |  | (Anderson et al., 1981) |
|  | 0.494 months: 3.32  [n = 1] |  | (Anderson et al., 1981) |
|  | 0.50 months: 3.32 ± 0.60 (18 %)  [n = 100] |  | (Erickson et al., 2013) |
|  | 0.51 months: 3.32 ± 0.44 (13 %)  [n = 2] |  | (Anderson et al., 1981) |
|  | 0.521 months: 3.85 ± 0.11 (3 %)  [n = 2] |  | (Anderson et al., 1981) |
|  | 0.524 months: 4.32 ± 0.81 (19 %)  [n = 2] |  | (Anderson et al., 1981) |
|  | 0.529 months: 5.62  [n = 1] |  | (Anderson et al., 1981) |
|  | 0.534 months: 2.21  [n = 1] |  | (Anderson et al., 1981) |
|  | 0.542 months: 3.07  [n = 1] |  | (Anderson et al., 1981) |
|  | 0.545 months: 3.24 ± 0.40 (12 %)  [n = 3] |  | (Anderson et al., 1981) |
|  | 0.55 months: 3.51  [n = 1] |  | (Anderson et al., 1981) |
|  | 0.56 months: 3.59 ± 0.74 (21 %)  [n = 34] |  | (Anderson et al., 1981; Maas et al., 1998) |
|  | 0.57 months: 2.85 ± 1.28 (45 %)  [n = 48] |  | (Hytten, 1994) |
|  | 0.587 months: 4.44  [n = 1] |  | (Anderson et al., 1981) |
|  | 0.590 months: 4.29 ± 0.05 (1 %)  [n = 2] |  | (Anderson et al., 1981) |
|  | 0.595 months: 3.65  [n = 1] |  | (Anderson et al., 1981) |
|  | 0.69 months: 4.12 ± 1.57 (38 %)  [n = 76] |  | (Lemons et al., 1982; Lubetzky et al., 2007) |
|  | 0.70 months: 2.87 ± 0.57 (20 %)  [n = 20] |  | (Lemons et al., 1982; Allen et al., 1991) |
|  | 0.71 months: 3.74 ± 0.96 (26 %)  [n = 35] |  | (Maas et al., 1998) |
|  | 0.72 months: 2.64 ± 0.89 (34 %)  [n = 58] |  | (Hytten, 1994) |
|  | 0.845 months: 4.83  [n = 1] |  | (Anderson et al., 1981) |
|  | 0.853 months: 4.13  [n = 1] |  | (Anderson et al., 1981) |
|  | 0.856 months: 3.84  [n = 1] |  | (Anderson et al., 1981) |
|  | 0.857 months: 3.53 ± 1.27 (36 %)  [n = 81] |  | (Hytten, 1994) |
|  | 0.871 months: 3.22  [n = 1] |  | (Anderson et al., 1981) |
|  | 0.878 months: 3.39  [n = 1] |  | (Anderson et al., 1981) |
|  | 0.879 months: 3.08  [n = 1] |  | (Anderson et al., 1981) |
|  | 0.882 months: 2.83 ± 0.11 (4%)  [n = 2] |  | (Anderson et al., 1981) |
|  | 0.883 months: 5.33 ± 0.22 (4 %)  [n = 2] |  | (Anderson et al., 1981) |
|  | 0.885 months: 3.32  [n = 1] |  | (Anderson et al., 1981) |
|  | 0.889 months: 4.54  [n = 1] |  | (Anderson et al., 1981) |
|  | 0.893 months: 3.08  [n = 1] |  | (Anderson et al., 1981) |
|  | 0.900 months: 3.66  [n = 1] |  | (Czosnykowska-Łukacka et al., 2018) |
|  | 0.903 months: 2.92 ± 0.06 (2 %)  [n = 10] |  | (Anderson et al., 1981; Luisa, 1995) |
|  | 0.913 months: 3.34  [n = 1] |  | (Anderson et al., 1981) |
|  | 0.916 months: 4.12 ± 2.63 (64 %)  [n = 2] |  | (Anderson et al., 1981) |
|  | 0.919 months: 3.29 ± 0.29 (9 %)  [n = 2] |  | (Anderson et al., 1981) |
|  | 0.922 months: 4.69  [n = 1] |  | (Anderson et al., 1981) |
|  | 0.923 months: 4.22 ± 1.42 (34 %)  [n = 73] |  | (Gross et al., 1980; Lemons et al., 1982; Lubetzky et al., 2007) |
|  | 0.93 months: 3.07 ± 0.20 (7 %)  [n = 7] |  | (Lemons et al., 1982) |
|  | 0.98 months: 4.92 ± 1.10 (21 %)  [n = 13] |  | (Luisa, 1995) |
|  | 1. months: 3.42 ± 1.60 (47 %) 2. [n = 720] |  | (Gross et al., 1980; Dewey and Lönnerdal, 1983; Butte et al., 1990a; Hytten, 1994; Mitoulas et al., 2002; Saarela et al., 2005; Vázquez-Román et al., 2014; Grote et al., 2016; Bzikowska-Jura et al., 2018; Kiboi et al., 2020) |
|  | 1.002 months: 3.34 ± 1.16 (35 %)  [n = 64] |  | (Wang et al., 1999; Saarela et al., 2005) |
|  | 1.01 months: 3.53 ± 0.63 (18 %)  [n = 24] |  | (Maas et al., 1998) |
|  | 1.14 months: 2.95 ± 1.62 (55 %)  [n = 24] |  | (Hytten, 1994) |
|  | 1.15 months: 4.08 ± 1.44 (35 %)  [n = 75] |  | (Maas et al., 1998; Lubetzky et al., 2007) |
|  | 1.29 months: 4.00  [n = 1] |  | (Hytten, 1994) |
|  | 1.30 months: 3.50 ± 0.70 (19 %)  [n = 16] |  | (Maas et al., 1998) |
|  | 1.38 months: 4.10 ± 0.60 (15 %)  [n = 10] |  | (Clark et al., 1982) |
|  | 1.39 months: 4.94 ± 2.27 (46 %)  [n = 55] |  | (Lemons et al., 1982; Lubetzky et al., 2007) |
|  | 1.40 months: 3.46 ± 0.30 (9 %)  [n = 9] |  | (Lemons et al., 1982) |
|  | 1.45 months: 3.47 ± 0.80 (22 %)  [n = 18] |  | (Maas et al., 1998) |
|  | 1.50 months: 3.19 ± 0.70 (20 %)  [n = 13] |  | (Allen et al., 1991) |
|  | 1.60 months: 3.53 ± 0.60 (18 %)  [n = 12] |  | (Maas et al., 1998) |
|  | 1.62 months: 4.86 ± 1.23 (25 %)  [n = 44] |  | (Lubetzky et al., 2007) |
|  | 1.84 months: 3.53 ± 3.50 (100 %)  [n = 73] |  | (Goldfarb and Savadove, 1991) |
|  | 1.86 months: 3.55 ± 0.65 (18 %)  [n = 2] |  | (Hytten, 1994) |
|  | 1.87 months: 3.76 ± 1.21 (32 %)  [n = 2] |  | (Czosnykowska-Łukacka et al., 2018) |
|  | 1.95 months: 3.09 ± 0.20 (7 %)  [n = 2] |  | (Czosnykowska-Łukacka et al., 2018) |
|  | 1.96 months: 4.58 ± 1.00 (21 %)  [n = 16] |  | (Luisa, 1995) |
|  | 2.00 months: 3.50 ± 1.17 (34 %)  [n = 222] |  | (Gross et al., 1980; Dewey and Lönnerdal, 1983; Mitoulas et al., 2002; Saarela et al., 2005; Grote et al., 2016; Żelaźniewicz and Pawłowski, 2019) |
|  | 2.01 months: 3.42 ± 1.34 (39 %)  [n = 24] |  | (Saarela et al., 2005) |
|  | 2.29 months: 3.21 ± 1.58 (49 %)  [n = 9] |  | (Hytten, 1994) |
|  | 2.32 months: 5.10  [n = 1] |  | (Czosnykowska-Łukacka et al., 2018) |
|  | 2.57 months: 2.70 ± 1.29 (47 %)  [n = 11] |  | (Hytten, 1994) |
|  | 2.77 months: 4.60 ± 0.70 (15 %)  [n = 10] |  | (Clark et al., 1982) |
|  | 2.83 months: 6.07 ± 2.50 (42 %)  [n = 36] |  | (Meier et al., 2006) |
|  | 2.86 months: 3.10  [n = 1] |  | (Hytten, 1994) |
|  | 2.94 months: 4.58 ± 1.70 (36 %)  [n = 18] |  | (Luisa, 1995) |
|  | 3.00 months: 3.51 ± 1.12 (32 %)  [n = 289] |  | (Gross et al., 1980; Dewey et al., 1984; Allen et al., 1991; Nommsen et al., 1991; Hytten, 1994; Arcus-Arth et al., 2005; Saarela et al., 2005; Grote et al., 2016; Bzikowska-Jura et al., 2018) |
|  | 3.01 months: 3.37 ± 1.52 (45 %)  [n = 14] |  | (Saarela et al., 2005) |
|  | 3.14 months: 4.39 ± 0.72 (16 %)  [n = 7] |  | (Hytten, 1994) |
|  | 3.20 months: 4.23 ± 1.20 (27 %)  [n = 15] |  | (Khan et al., 2013a) |
|  | 3.29 months: 3.77 ± 1.20 (32 %)  [n = 8] |  | (Hytten, 1994) |
|  | 3.50 months: 4.67 ± 1.90 (41 %)  [n = 19] |  | (Glew et al., 2011) |
|  | 3.69 months: 5.23 ± 0.31 (6 %)  [n = 58] |  | (Clark et al., 1982; Goldfarb and Savadove, 1991) |
|  | 3.82 months: 3.66  [n = 1] |  | (Czosnykowska-Łukacka et al., 2018) |
|  | 3.83 months: 4.11 ± 0.80 (19 %)  [n = 142] |  | (Kent et al., 2006) |
|  | 3.86 months: 4.30  [n = 1] |  | (Hytten, 1994) |
|  | 3.90 months: 4.56  [n = 1] |  | (Czosnykowska-Łukacka et al., 2018) |
|  | 3.92 months: 4.62 ± 1.90 (40 %)  [n = 16] |  | (Luisa, 1995) |
|  | 4.00 months: 4.38 ± 3.10 (71 %)  [n = 206] |  | (Gross et al., 1980; Dewey and Lönnerdal, 1983; Butte et al., 1990a; Daly et al., 1993; Hytten, 1994; Mitoulas et al., 2002; Mandel et al., 2005; Saarela et al., 2005) |
|  | 4.01 months: 2.94 ± 1.37 (47 %)  [n = 10] |  | (Saarela et al., 2005) |
|  | 4.34 months: 4.29 ± 1.81 (42 %)  [n = 139] |  | (Butte et al., 1984; O’Neill et al., 2013) |
|  | 4.57 months: 3.73 ± 0.99 (27 %)  [n = 6] |  | (Hytten, 1994) |
|  | 4.86 months: 3.50  [n = 1] |  | (Hytten, 1994) |
|  | 4.90 months: 4.36 ± 1.70 (38 %)  [n = 14] |  | (Luisa, 1995) |
|  | 4.95 months: 3.89  [n = 1] |  | (Czosnykowska-Łukacka et al., 2018) |
|  | 5.00 months: 3.90 ± 2.31 (59 %)  [n = 231] |  | (Dewey and Lönnerdal, 1983; Garza et al., 1983; Dewey et al., 1984; Daly et al., 1993; Saarela et al., 2005; Kiboi et al., 2020) |
|  | 5.01 months: 3.41 ± 1.16 (34 %)  [n = 9] |  | (Saarela et al., 2005) |
|  | 5.29 months: 3.41 ± 0.97 (28 %)  [n = 6] |  | (Hytten, 1994) |
|  | 5.43 months: 3.77 ± 0.57 (15 %)  [n = 6] |  | (Hytten, 1994) |
|  | 5.50 months: 5.30 ± 2.40 (46 %)  [n = 6] |  | (Garza et al., 1983) |
|  | 5.53 months: 3.94  [n = 41] |  | (Goldfarb and Savadove, 1991) |
|  | 5.56 months: 2.47 ± 0.70 (26 %)  [n = 15] |  | (Agne-Djigo et al., 2013) |
|  | 5.71 months: 3.43 ± 0.28 (8 %)  [n = 6] |  | (Hytten, 1994) |
|  | 5.86 months: 3.50 ± 0.62 (18 %)  [n = 5] |  | (Hytten, 1994) |
|  | 5.88 months: 4.30 ± 2.00 (46 %)  [n = 18] |  | (Luisa, 1995) |
|  | 5.92 months: 3.56 ± 0.74 (21 %)  [n = 3] |  | (Czosnykowska-Łukacka et al., 2018) |
|  | 6.00 months: 3.61 ± 1.28 (35 %)  [n = 235] |  | (Dewey and Lönnerdal, 1983; Garza et al., 1983; Allen et al., 1991; Nommsen et al., 1991; Hytten, 1994; Mitoulas et al., 2002; Arcus-Arth et al., 2005; Saarela et al., 2005; Grote et al., 2016; Bzikowska-Jura et al., 2018) |
|  | 6.01 months: 3.26 ± 0.99 (30 %)  [n = 5] |  | (Saarela et al., 2005) |
|  | 6.50 months: 6.90 ± 2.45 (35 %)  [n = 6] |  | (Garza et al., 1983) |
|  | 6.52 months: 2.55  [n = 1] |  | (Czosnykowska-Łukacka et al., 2018) |
|  | 6.71 months: 4.16 ± 0.93 (22 %)  [n = 7] |  | (Hytten, 1994) |
|  | 6.86 months: 2.97 ± 1.02 (35 %)  [n = 18] |  | (Hytten, 1994) |
|  | 6.97 months: 2.44 ± 0.45 (19 %)  [n = 2] |  | (Czosnykowska-Łukacka et al., 2018) |
|  | 7.00 months: 2.28 ± 1.08 (48 %)  [n = 81] |  | (Garza et al., 1983; Hytten, 1994; Butts et al., 2018) |
|  | 7.14 months: 3.24 ± 0.71 (22 %)  [n = 18] |  | (Hytten, 1994) |
|  | 7.50 months: 7.50 ± 3.90 (52 %)  [n = 6] |  | (Garza et al., 1983) |
|  | 7.57 months: 3.13 ± 0.50 (16 %)  [n = 21] |  | (Hytten, 1994; Maas et al., 1998) |
|  | 7.95 months: 4.15 ± 1.12 (27 %)  [n = 2] |  | (Czosnykowska-Łukacka et al., 2018) |
|  | 8.00 months: 6.71 ± 11.73 (175 %)  [n = 46] |  | (Czosnykowska-Łukacka et al., 2018) |
|  | 8.29 months: 4.50  [n = 1] |  | (Hytten, 1994) |
|  | 8.68 months: 3.54 ± 0.70 (20 %)  [n = 40] |  | (Butte et al., 1984) |
|  | 8.85 months: 2.66  [n = 1] |  | (Czosnykowska-Łukacka et al., 2018) |
|  | 8.86 months: 4.24 ± 1.10 (26 %)  [n = 12] |  | (Hytten, 1994) |
|  | 9.00 months: 4.29 ± 1.72 (40 %)  [n = 145] |  | (Dewey et al., 1984; Nommsen et al., 1991; Daly et al., 1993; Mitoulas et al., 2002; Arcus-Arth et al., 2005) |
|  | 9.90 months: 3.19  [n = 1] |  | (Czosnykowska-Łukacka et al., 2018) |
|  | 10.87 months: 5.04  [n = 1] |  | (Czosnykowska-Łukacka et al., 2018) |
|  | 10.95 months: 4.50  [n = 1] |  | (Czosnykowska-Łukacka et al., 2018) |
|  | 11.00 months: 4.00 ± 2.00 (50 %)  [n = 19] |  | (Perrin et al., 2017) |
|  | 11.84 months: 3.35 ± 0.36 (11 %)  [n = 4] |  | (Czosnykowska-Łukacka et al., 2018) |
|  | 11.92 months: 2.29  [n = 1] |  | (Czosnykowska-Łukacka et al., 2018) |
|  | 12.00 months: 3.93 ± 1.86 (47 %)  [n = 89] |  | (Nommsen et al., 1991; Arcus-Arth et al., 2005; Perrin et al., 2017) |
| Milk total protein (%) | 0.10 months: 2.06 ± 0.49 (24 %)  [n = 56] | $\text{Milk total protein (\%)}\text{ }\text{=} \text{1.219+}\text{1.127 e}^{\text{(-5.058 × PpT)}}$ | (Fleishaker et al., 1989; Paduraru et al., 2019; Sahin et al., 2020) |
|  | 0.11 months: 1.09 ± 0.46 (42 %)  [n = 26] |  | (Pamblanco et al., 1986) |
|  | 0.12 months: 2.15 ± 0.86 (40 %)  [n = 31] |  | (Huang and Hu, 2020) |
|  | 0.17 months: 1.57 ± 0.28 (18 %)  [n = 20] |  | (Fleishaker et al., 1989; Hsu et al., 2014) |
|  | 0.23 months: 1.79 ± 0.29 (16 %)  [n = 104] |  | (Fleishaker et al., 1989; Saarela et al., 2005; Paduraru et al., 2019; Sahin et al., 2020) |
|  | 0.27 months: 0.72 ± 0.35 (49 %)  [n = 26] |  | (Pamblanco et al., 1986) |
|  | 0.38 months: 1.66 ± 0.85 (51 %)  [n = 34] |  | (Huang and Hu, 2020) |
|  | 0.43 months: 0.62 ± 0.30 (49 %)  [n = 26] |  | (Pamblanco et al., 1986) |
|  | 0.46 months: 1.40 ± 0.38 (27 %)  [n = 56] |  | (Fleishaker et al., 1989; Paduraru et al., 2019; Sahin et al., 2020) |
|  | 0.69 months: 1.43 ± 0.03 (2 %)  [n = 30] |  | (Paduraru et al., 2019) |
|  | 0.75 months: 1.27 ± 0.29 (23 %)  [n = 119] |  | (Kim et al., 2019) |
|  | 0.76 months: 1.71 ± 0.83 (49 %)  [n = 34] |  | (Huang and Hu, 2020) |
|  | 0.88 months: 0.51 ± 0.11 (21 %)  [n = 26] |  | (Pamblanco et al., 1986) |
|  | 0.92 months: 1.58 ± 0.60 (38 %)  [n = 21] |  | (Sahin et al., 2020) |
|  | 0.99 months: 1.28 ± 0.02 (2 %)  [n = 30] |  | (Paduraru et al., 2019) |
|  | 1.02 months: 1.45 ± 0.19 (13 %)  [n = 46] |  | (Saarela et al., 2005) |
|  | 1.04 months: 0.92 ± 0.12 (13 %)  [n = 15] |  | (Hsu et al., 2014) |
|  | 1.98 months: 1.10 ± 0.02 (1 %)  [n = 30] |  | (Paduraru et al., 2019) |
|  | 2.00 months: 1.17 ± 0.22 (19 %)  [n = 32] |  | (Huang and Hu, 2020) |
|  | 2.05 months: 1.32 ± 0.22 (17 %)  [n = 43] |  | (Saarela et al., 2005) |
|  | 3.17 months: 1.26 ± 0.14 (11 %)  [n = 37] |  | (Saarela et al., 2005) |
|  | 4.09 months: 1.25 ± 0.27 (22 %)  [n = 34] |  | (Saarela et al., 2005) |
|  | 4.47 months: 0.95 ± 0.15 (16 %)  [n = 40] |  | (Huang and Hu, 2020) |
|  | 5.12 months: 1.16 ± 0.12 (10 %)  [n = 25] |  | (Saarela et al., 2005) |
|  | 6.14 months: 1.14 ± 0.11 (10 %)  [n = 20] |  | (Saarela et al., 2005) |
| Milk water content (%) | 0.12 months: 87.20  [n = 1] | Milk water content (%) = 87.5 | (Macy, 1949) |
|  | 0.15 months: 88.08 ± 0.98 (1 %)  [n = 34] |  | (Emmett and Rogers, 1997; Pecka-Kiełb et al., 2018; Huang and Hu, 2020) |
|  | 0.25 months: 86.50 ± 1.90 (2 %)  [n = 23] |  | (Khan et al., 2013b) |
|  | 0.43 months: 86.40  [n = 1] |  | (Macy, 1949) |
|  | 0.50 months: 86.50 ± 1.60 (2 %)  [n = 23] |  | (Khan et al., 2013b) |
|  | 0.52 months: 87.00 ± 0.90 (1 %)  [n = 34] |  | (Huang and Hu, 2020) |
|  | 0.75 months: 87.20 ± 1.70 (2 %)  [n = 23] |  | (Khan et al., 2013b) |
|  | 0.90 months: 87.00  [n = 1] |  | (Czosnykowska-Łukacka et al., 2018) |
|  | 1. months: 87.88 ± 0.87 (1 %) 2. [n = 75] |  | (Bzikowska-Jura et al., 2018; Czosnykowska-Łukacka et al., 2018; Huang and Hu, 2020) |
|  | 1.50 months: 87.38 ± 1.03 (1 %)  [n = 682] |  | (Christie et al., 1977; Bzikowska-Jura et al., 2020) |
|  | 1.80 months: 87.32 ± 0.37 (1 %)  [n = 78] |  | (Butts et al., 2018) |
|  | 2.00 months: 87.90 ± 0.82 (1 %)  [n = 4] |  | (Czosnykowska-Łukacka et al., 2018) |
|  | 2.50 months: 88.15 ± 1.41 (2 %)  [n = 33] |  | (Czosnykowska-Łukacka et al., 2018; Huang and Hu, 2020) |
|  | 3.00 months: 88.60 ± 1.70 (2 %)  [n = 22] |  | (Bzikowska-Jura et al., 2018) |
|  | 3.90 months: 87.90 ± 0.24 (1 %)  [n = 10] |  | (Christie et al., 1977; Czosnykowska-Łukacka et al., 2018) |
|  | 4.00 months: 88.11 ± 0.94 (1 %)  [n = 3] |  | (Czosnykowska-Łukacka et al., 2018) |
|  | 4.90 months: 88.00  [n = 1] |  | (Czosnykowska-Łukacka et al., 2018) |
|  | 5.40 months: 88.60  [n = 1] |  | (Czosnykowska-Łukacka et al., 2018) |
|  | 5.90 months: 87.90  [n = 1] |  | (Czosnykowska-Łukacka et al., 2018) |
|  | 6.00 months: 88.11 ± 2.05 (2 %)  [n = 64] |  | (Bzikowska-Jura et al., 2018; Huang and Hu, 2020) (Khan et al., 2013b; Czosnykowska-Łukacka et al., 2018) |
|  | 6.50 months: 88.00  [n = 1] |  | (Czosnykowska-Łukacka et al., 2018) |
|  | 6.90 months: 89.75 ± 0.15 (1 %)  [n = 2] |  | (Czosnykowska-Łukacka et al., 2018) |
|  | 8.00 months: 87.30 ± 0.60 (1 %)  [n = 2] |  | (Czosnykowska-Łukacka et al., 2018) |
|  | 9.00 months: 85.93 ± 1.95 (2 %)  [n = 8] |  | (Khan et al., 2013b; Czosnykowska-Łukacka et al., 2018) |
|  | 9.90 months: 88.50  [n = 1] |  | (Czosnykowska-Łukacka et al., 2018) |
|  | 10.90 months: 86.70  [n = 1] |  | (Czosnykowska-Łukacka et al., 2018) |
|  | 11.00 months: 87.20  [n = 1] |  | (Czosnykowska-Łukacka et al., 2018) |
|  | 11.90 months: 88.18 ± 0.58 (1 %)  [n = 4] |  | (Czosnykowska-Łukacka et al., 2018) |
|  | 12.00 months: 88.39 ± 2.09 (2 %)  [n = 53] |  | (Khan et al., 2013b; Huang and Hu, 2020) |
| Milk pH | 0.07 months: 7.43 ± 0.24 (3 %)  [n = 38] | $\text{Milk pH}\text{ }\text{=} \text{0.443 }\text{e}^{\text{(-13.07 × PpT)}}\text{ + }\text{7.167 e}^{\text{(0.0023 × PpT)}}$ | (Ansell et al., 1977; Morriss Jr et al., 1986) |
|  | 0.08 months: 7.60 ± 0.45 (6 %)  [n = 5] |  | (Ogundele, 2002) |
|  | 0.10 months: 7.11 ± 0.25 (3 %)  [n = 16] |  | (Nagasawa et al., 1974; Ansell et al., 1977) |
|  | 0.12 months: 7.26 ± 0.22 (3 %)  [n = 5] |  | (Fleishaker et al., 1989) |
|  | 0.13 months: 7.17 ± 0.21 (3 %)  [n = 22] |  | (Nagasawa et al., 1974; Ansell et al., 1977; Sunarić et al., 2016) |
|  | 0.17 months: 7.05 ± 0.27 (4 %)  [n = 15] |  | (Nagasawa et al., 1974; Ansell et al., 1977) |
|  | 0.18 months: 7.16 ± 0.22 (3 %)  [n = 33] |  | (Morriss Jr et al., 1986; Fleishaker et al., 1989) |
|  | 0.20 months: 7.17 ± 0.28 (4 %)  [n = 17] |  | (Nagasawa et al., 1974; Ansell et al., 1977) |
|  | 0.28 months: 7.22 ± 0.59 (8 %)  [n = 299] |  | (Harrison and Peat, 1972; Nagasawa et al., 1974; Ansell et al., 1977; Malhotra, 1982; Morriss Jr et al., 1986; Fleishaker et al., 1989; Cho et al., 2012; Sunarić et al., 2016; Caldeo et al., 2021) |
|  | 0.54 months: 7.15 ± 0.20 (3 %)  [n = 60] |  | (Morriss Jr et al., 1986; Fleishaker et al., 1989; Ogundele, 2002; Caldeo et al., 2021) |
|  | 0.74 months: 6.99 ± 0.25 (4 %)  [n = 39] |  | (Nagasawa et al., 1974; Morriss Jr et al., 1986; Allen et al., 1991; Sunarić et al., 2016) |
|  | 1.01 months: 7.13 ± 0.16 (2 %)  [n = 63] |  | (Nagasawa et al., 1974; Hamosh et al., 1996; Slutzah et al., 2010; Caldeo et al., 2021) |
|  | 1.20 months: 7.29 ± 0.33 (5 %)  [n = 6] |  | (Nagasawa et al., 1974; Ogundele, 2002) |
|  | 1.50 months: 6.80 ± 0.07 (1 %)  [n = 13] |  | (Allen et al., 1991) |
|  | 1.83 months: 7.40  [n = 1] |  | (Nagasawa et al., 1974) |
|  | 2.00 months: 7.14 ± 0.18 (3 %)  [n = 60] |  | (Morriss Jr et al., 1986; Caldeo et al., 2021) |
|  | 2.21 months: 7.12 ± 0.14 (2 %)  [n = 42] |  | (Nagasawa et al., 1974; Handa et al., 2014) |
|  | 2.60 months: 6.92  [n = 1] |  | (Nagasawa et al., 1974) |
|  | 2.77 months: 6.90  [n = 1] |  | (Nagasawa et al., 1974) |
|  | 3.00 months: 7.37 ± 0.97 (13 %)  [n = 192] |  | (Nagasawa et al., 1974; Malhotra, 1982; Morriss Jr et al., 1986; Allen et al., 1991; Oo et al., 1995; Caldeo et al., 2021) |
|  | 3.23 months: 6.98  [n = 1] |  | (Nagasawa et al., 1974) |
|  | 3.98 months: 7.22 ± 1.20 (17 %)  [n = 166] |  | (Malhotra, 1982; Schwab et al., 2019) (Morriss Jr et al., 1986) |
|  | 4.95 months: 7.26 ± 0.24 (3 %)  [n = 29] |  | (Morriss Jr et al., 1986; Carey et al., 1997) |
|  | 5.50 months: 6.96 ± 0.07 (1 %)  [n = 5] |  | (Hamosh et al., 1996) |
|  | 6.00 months: 7.14 ± 0.24 (3 %)  [n = 33] |  | (Morriss Jr et al., 1986; Allen et al., 1991) |
|  | 6.38 months: 7.13 ± 0.14 (2 %)  [n = 12] |  | (Oo et al., 1995) |
|  | 7.00 months: 7.21 ± 0.20 (3 %)  [n = 19] |  | (Morriss Jr et al., 1986; Ilett et al., 2002) |
|  | 8.00 months: 7.30 ± 0.16 (2 %)  [n = 10] |  | (Morriss Jr et al., 1986) |
|  | 9.00 months: 7.28 ± 0.13 (2 %)  [n = 11] |  | (Morriss Jr et al., 1986) |
|  | 10.00 months: 7.40 ± 0.25 (3 %)  [n = 12] |  | (Morriss Jr et al., 1986) |
| Milk volume (L/day) | 0.03 months: 0.05 ± 0.05 (111 %)  [n = 14] | If PpT <= 6 then  $\text{Milk volume }\left( \text{L}/\text{day} \right)\text{ }\text{= }\frac{\text{0.81 × }\text{PpT}^{\text{4.37}}}{\text{0.1}^{\text{4.37}}\text{+ }\text{PpT}^{\text{4.37}}}$  Else  $\text{Milk volume }\left( \text{L}/\text{day} \right)\text{ = 1.619 }\text{e}^{(\text{-0.116 }\times\text{PpT)}}$ | (Roderuck et al., 1946; Neville et al., 1988) |
|  | 0.07 months: 0.17 ± 0.10 (60 %)  [n = 17] |  | (Roderuck et al., 1946; Neville et al., 1988) |
|  | 0.10 months: 0.46 ± 0.20 (44 %)  [n = 18] |  | (Roderuck et al., 1946; Neville et al., 1988) |
|  | 0.13 months: 0.69 ± 0.31 (44 %)  [n =18] |  | (Roderuck et al., 1946; Neville et al., 1988) |
|  | 0.16 months: 0.72 ± 0.31 (43 %)  [n = 19] |  | (Roderuck et al., 1946; Neville et al., 1988) |
|  | 0.20 months: 0.78 ± 0.38 (49 %)  [n = 17] |  | (Roderuck et al., 1946; Neville et al., 1988) |
|  | 0.23 months: 0.87 ± 0.35 (40 %)  [n = 18] |  | (Roderuck et al., 1946; Neville et al., 1988) |
|  | 0.26 months: 0.88 ± 0.40 (46 %)  [n = 17] |  | (Roderuck et al., 1946; Neville et al., 1988) |
|  | 0.30 months: 0.88 ± 0.39 (44 %)  [n = 18] |  | (Roderuck et al., 1946; Neville et al., 1988) |
|  | 0.32 months: 0.51 ± 0.17 (33 %)  [n = 2] |  | (Kent et al., 2016) |
|  | 0.33 months: 0.90 ± 0.43 (48 %)  [n = 17] |  | (Roderuck et al., 1946; Neville et al., 1988) |
|  | 0.36 months: 0.65 ± 0.17 (26 %)  [n = 8] |  | (Neville et al., 1988) |
|  | 0.44 months: 0.62  [n = 1] |  | (Kent et al., 2016) |
|  | 0.46 months: 0.81 ± 0.24 (29 %)  [n = 16] |  | (Saint et al., 1984; Neville et al., 1988) |
|  | 0.60 months: 0.58 ± 0.07 (11 %)  [n = 2] |  | (Kent et al., 2016) |
|  | 0.67 months: 0.77 ± 0.05 (7 %)  [n = 2] |  | (Kent et al., 2016) |
|  | 0.69 months: 0.70 ± 0.10 (15 %)  [n = 11] |  | (Neville et al., 1988; Kent et al., 2016) |
|  | 0.72 months: 0.58  [n = 1] |  | (Kent et al., 2016) |
|  | 0.761 months: 0.91  [n = 1] |  | (Kent et al., 2016) |
|  | 0.763 months: 0.75  [n = 1] |  | (Kent et al., 2016) |
|  | 0.79 months: 0.90  [n = 1] |  | (Kent et al., 2016) |
|  | 0.90 months: 1.01  [n = 1] |  | (Kent et al., 2016) |
|  | 0.92 months: 0.91 ± 0.28 (31 %)  [n = 19] |  | (Saint et al., 1984; Neville et al., 1988) |
|  | 1.00 months: 0.87 ± 0.23 (26 %)  [n = 40] |  | (Rattigan et al., 1981; Cox et al., 1996; Kent et al., 1999; Cregan et al., 2002; Mitoulas et al., 2002) |
|  | 1.10 months: 0.72 ± 0.12 (16 %)  [n = 10] |  | (Kent et al., 2013) |
|  | 1.11 months: 0.80 ± 0.18 (23 %)  [n = 18] |  | (Kent et al., 2013) |
|  | 1.14 months: 0.78 ± 0.20 (26 %)  [n = 18] |  | (Kent et al., 2013) |
|  | 1.15 months: 0.73 ± 0.14 (19 %)  [n = 12] |  | (Neville et al., 1988) |
|  | 1.38 months: 0.74 ± 0.11 (15 %)  [n = 12] |  | (Neville et al., 1988) |
|  | 1.46 months: 0.43  [n = 1] |  | (Roderuck et al., 1946) |
|  | 1.49 months: 0.39  [n = 1] |  | (Roderuck et al., 1946) |
|  | 1.57 months: 0.41  [n = 1] |  | (Roderuck et al., 1946) |
|  | 1.61 months: 0.75 ± 0.12 (17 %)  [n = 10] |  | (Neville et al., 1988) |
|  | 1.65 months: 0.42  [n = 1] |  | (Roderuck et al., 1946) |
|  | 1.75 months: 0.75 ± 0.01 (2 %)  [n = 2] |  | Cregan et al., 2002 |
|  | 1.84 months: 0.74 ± 0.11 (15 %)  [n = 13] |  | (Neville et al., 1988) |
|  | 1.89 months: 0.90  [n = 1] |  | (Roderuck et al., 1946) |
|  | 1.95 months: 0.95 ± 0.02 (2 %)  [n = 2] |  | (Roderuck et al., 1946) |
|  | 2.00 months: 0.82 ± 0.20 (24 %)  [n = 35] |  | (Roderuck et al., 1946; Cox et al., 1996; Kent et al., 1999; Cregan et al., 2002; Mitoulas et al., 2002) |
|  | 2.22 months: 0.74  [n = 1] |  | (Roderuck et al., 1946) |
|  | 2.25 months: 0.71 ± 0.17 (24 %)  [n = 2] |  | (Cregan et al., 2002) |
|  | 2.27 months: 0.24  [n = 1] |  | (Roderuck et al., 1946) |
|  | 2.30 months: 0.56 ± 0.26 (46 %)  [n = 4] |  | (Roderuck et al., 1946) |
|  | 2.36 months: 0.83  [n = 1] |  | (Roderuck et al., 1946) |
|  | 2.38 months: 0.51 ± 0.20 (38 %)  [n = 2] |  | (Roderuck et al., 1946) |
|  | 2.44 months: 0.36  [n = 1] |  | (Roderuck et al., 1946) |
|  | 2.46 months: 0.63 ± 0.05 (7 %)  [n = 2] |  | (Roderuck et al., 1946) |
|  | 2.49 months: 0.84  [n = 1] |  | (Roderuck et al., 1946) |
|  | 2.50 months: 0.97 ± 0.14 (15 %)  [n = 2] |  | (Cregan et al., 2002) |
|  | 2.55 months: 0.72 ± 0.03 (4 %)  [n = 2] |  | (Roderuck et al., 1946) |
|  | 2.57 months: 0.84 ± 0.06 (7 %)  [n = 3] |  | (Roderuck et al., 1946) |
|  | 2.60 months: 0.75  [n = 1] |  | (Roderuck et al., 1946) |
|  | 2.63 months: 0.88 ± 0.02 (2 %)  [n = 2] |  | (Roderuck et al., 1946) |
|  | 2.68 months: 0.93  [n = 1] |  | (Roderuck et al., 1946) |
|  | 2.71 months: 0.92  [n = 1] |  | (Roderuck et al., 1946) |
|  | 2.75 months: 0.87  [n = 1] |  | (Cregan et al., 2002) |
|  | 2.76 months: 0.94  [n = 1] |  | (Roderuck et al., 1946) |
|  | 2.82 months: 1.04 ± 0.03 (3 %)  [n = 2] |  | (Roderuck et al., 1946) |
|  | 2.90 months: 1.02  [n = 1] |  | (Roderuck et al., 1946) |
|  | 2.96 months: 0.77 ± 0.13 (16 %)  [n = 12] |  | (Neville et al., 1988) |
|  | 3.00 months: 1.17 ± 0.17 (14 %)  [n = 6] |  | (Rattigan et al., 1981; Cox et al., 1996) |
|  | 3.09 months: 0.53  [n = 1] |  | (Roderuck et al., 1946) |
|  | 3.14 months: 0.60  [n = 1] |  | (Roderuck et al., 1946) |
|  | 3.17 months: 0.69  [n = 1] |  | (Roderuck et al., 1946) |
|  | 3.25 months: 0.82 ± 0.14 (17 %)  [n = 2] |  | (Roderuck et al., 1946; Cregan et al., 2002) |
|  | 3.75 months: 0.68 ± 0.14 (20 %)  [n = 2] |  | (Cregan et al., 2002) |
|  | 3.95 months: 0.75 ± 0.10 (14 %)  [n = 13] |  | (Neville et al., 1988) |
|  | 4.00 months: 0.83 ± 0.16 (19 %)  [n = 31] |  | (Cox et al., 1996; Kent et al., 1999; Mitoulas et al., 2002) |
|  | 4.72 months: 0.30 ± 0.03 (8 %)  [n = 3] |  | (Roderuck et al., 1946) |
|  | 4.80 months: 0.32  [n = 1] |  | (Roderuck et al., 1946) |
|  | 4.83 months: 0.37  [n = 1] |  | (Roderuck et al., 1946) |
|  | 4.93 months: 0.85 ± 0.12 (14 %)  [n = 13] |  | (Neville et al., 1988) |
|  | 4.97 months: 0.69  [n = 1] |  | (Roderuck et al., 1946) |
|  | 5.00 months: 0.78  [n = 1] |  | (Cregan et al., 2002) |
|  | 5.02 months: 0.66  [n = 1] |  | (Roderuck et al., 1946) |
|  | 5.05 months: 0.67  [n = 1] |  | (Roderuck et al., 1946) |
|  | 5.08 months: 0.84 ± 0.21 (25 %)  [n = 16] |  | (Kent et al., 2018) |
|  | 5.29 months: 0.79 ± 0.04 (5 %)  [n = 2] |  | (Roderuck et al., 1946) |
|  | 5.32 months: 0.89  [n = 1] |  | (Roderuck et al., 1946) |
|  | 5.35 months: 0.98  [n = 1] |  | (Roderuck et al., 1946) |
|  | 5.37 months: 1.00  [n = 1] |  | (Roderuck et al., 1946) |
|  | 5.50 months: 0.98  [n = 1] |  | (Cregan et al., 2002) |
|  | 5.65 months: 0.20  [n = 1] |  | (Roderuck et al., 1946) |
|  | 5.70 months: 0.26 ± 0.03 (13 %)  [n = 2] |  | (Roderuck et al., 1946) |
|  | 5.78 months: 0.30  [n = 1] |  | (Roderuck et al., 1946) |
|  | 5.92 months: 0.81 ± 0.12 (15 %)  [n = 22] |  | (Neville et al., 1988) |
|  | 6.00 months: 0.87 ± 0.24 (28 %)  [n = 36] |  | (Rattigan et al., 1981; Cox et al., 1996; Kent et al., 1999; Cregan et al., 2002; Mitoulas et al., 2002) |
|  | 6.68 months: 0.88 ± 0.04 (5 %)  [n = 2] |  | (Roderuck et al., 1946) |
|  | 6.71 months: 0.90  [n = 1] |  | (Roderuck et al., 1946) |
|  | 6.76 months: 0.92  [n = 1] |  | (Roderuck et al., 1946) |
|  | 6.79 months: 0.89  [n = 1] |  | (Roderuck et al., 1946) |
|  | 6.90 months: 0.78 ± 0.15 (19 %)  [n = 18] |  | (Neville et al., 1988) |
|  | 7.85 months: 0.60 ± 0.01 (2 %)  [n = 2] |  | (Roderuck et al., 1946) |
|  | 7.88 months: 0.65  [n = 1] |  | (Roderuck et al., 1946) |
|  | 7.89 months: 0.69 ± 0.19 (28 %)  [n = 14] |  | (Neville et al., 1988) |
|  | 7.90 months: 0.72  [n =1] |  | (Roderuck et al., 1946) |
|  | 7.93 months: 0.75  [n = 1] |  | (Roderuck et al., 1946) |
|  | 8.48 months: 0.73  [n = 1] |  | (Roderuck et al., 1946) |
|  | 8.53 months: 0.61 ± 0.22 (3 %)  [n = 2] |  | (Roderuck et al., 1946) |
|  | 8.58 months: 0.71  [n = 1] |  | (Roderuck et al., 1946) |
|  | 8.61 months: 0.67  [n = 1] |  | (Roderuck et al., 1946) |
|  | 8.88 months: 0.70 ± 0.21 (30 %)  [n = 17] |  | (Neville et al., 1988) |
|  | 9.00 months: 0.73 ± 0.25 (34 %)  [n = 15] |  | (Rattigan et al., 1981; Kent et al., 1999; Mitoulas et al., 2002) |
|  | 9.86 months: 0.58 ± 0.24 (42 %)  [n = 13] |  | (Roderuck et al., 1946; Neville et al., 1988) |
|  | 9.94 months: 0.37  [n = 1] |  | (Roderuck et al., 1946) |
|  | 9.97 months: 0.37  [n = 1] |  | (Roderuck et al., 1946) |
|  | 10.85 months: 0.58 ± 0.23 (40 %)  [n = 9] |  | (Neville et al., 1988) |
|  | 11.84 months: 0.43 ± 0.25 (59 %)  [n = 9] |  | (Neville et al., 1988) |
|  | 12.00 months: 0.58 ± 0.27 (47 %)  [n = 14] |  | (Rattigan et al., 1981; Kent et al., 1999; Mitoulas et al., 2002) |
| Milk intake (L/kg/day) | 0.03 months: 0.01 ± 0.01 (95 %)  [n = 62] | If PpT <= 1 then  $Milk intake (L/kg/day) = \frac{\text{0.181 × }\text{PpT}^{\text{2.411}}}{\text{0.114}^{\text{2.411}}\text{+ }\text{PpT}^{\text{2.411}}}$  Else  $\text{Milk intake (L/kg/day) = 0.004 + }\left( \text{0.208 }-\text{0.004} \right) \text{e}^{\text{ (}- \text{0.15 × PpT)}}$ | (Houston et al., 1983; Casey et al., 1986; Dollberg et al., 2001; Evans et al., 2003) |
|  | 0.07 months: 0.02 ± 0.02 (77 %)  [n = 143] |  | (Houston et al., 1983; Casey et al., 1986; Arthur et al., 1989a; Dollberg et al., 2001; Evans et al., 2003) |
|  | 0.09 months: 0.19  [n = 12] |  | (Allen et al., 1991) |
|  | 0.10 months: 0.07 ± 0.03 (47 %)  [n = 128] |  | (Houston et al., 1983; Casey et al., 1986; Arthur et al., 1989a; Evans et al., 2003) |
|  | 0.13 months: 0.11 ± 0.04 (32 %)  [n = 129] |  | (Houston et al., 1983; Casey et al., 1986; Arthur et al., 1989a; Evans et al., 2003) |
|  | 0.16 months: 0.13 ± 0.04 (30 %)  [n = 129] |  | (Houston et al., 1983; Casey et al., 1986; Arthur et al., 1989a; Evans et al., 2003) |
|  | 0.20 months: 0.14 ± 0.04 (26 %)  [n = 100] |  | (Arthur et al., 1989a; Evans et al., 2003) |
|  | 0.23 months: 0.07  [n = 1] |  | (English, 1985) |
|  | 0.26 months: 0.18 ± 0.06 (35 %)  [n = 12] |  | (Allen et al., 1991) |
|  | 0.43 months: 0.20  [n = 12] |  | (Allen et al., 1991) |
|  | 0.46 months: 0.13 ± 0.03 (21 %)  [n = 13] |  | (English, 1985; Bhutta et al., 2000) |
|  | 0.49 months: 0.09 ± 0.02 (18 %)  [n = 59] |  | (Amatayakul et al., 1999) |
|  | 0.65 months: 0.20 ± 0.02 (12 %)  [n = 12] |  | (Allen et al., 1991) |
|  | 0.69 months: 0.20  [n = 1] |  | (English, 1985) |
|  | 0.86 months: 0.21  [n = 12] |  | (Allen et al., 1991) |
|  | 0.92 months: 0.20  [n = 1] |  | (English, 1985) |
|  | 1.00 months: 0.18 ± 0.03 (16 %)  [n = 117] |  | (Butte et al., 1990a; Butte et al., 1990b; Alvear et al., 2000; Bandara et al., 2015) |
|  | 1.04 months: 0.18 ± 0.03 (16 %)  [n = 12] |  | (Allen et al., 1991) |
|  | 1.10 months: 0.17 ± 0.03 (16 %)  [n = 11] |  | (Butte et al., 1983; Kent et al., 2013) |
|  | 1.11 months: 0.20 ± 0.04 (23 %)  [n = 18] |  | (Kent et al., 2013) |
|  | 1.12 months: 0.15 ± 0.03 (20 %)  [n = 59] |  | (de Carvalho et al., 1982) |
|  | 1.14 months: 0.19 ± 0.05 (26 %)  [n = 18] |  | (Kent et al., 2013) |
|  | 1.15 months: 0.16 ± 0.02 (16 %)  [n = 38] |  | (Butte et al., 1984; English, 1985) |
|  | 1.17 months: 0.24 ± 0.06 (26 %)  [n = 6] |  | (Kent et al., 2013) |
|  | 1.20 months: 0.17  [n = 1] |  | (Butte et al., 1983) |
|  | 1.25 months: 0.18  [n = 12] |  | (Allen et al., 1991) |
|  | 1.32 months: 0.16 ± 0.03 (19 %)  [n = 10] |  | (Motil et al., 1997) |
|  | 1.38 months: 0.20 ± 0.02 (9 %)  [n = 2] |  | (English, 1985; Ramsay et al., 2005) |
|  | 1.48 months: 0.08 ± 0.01 (18 %)  [n = 58] |  | (Amatayakul et al., 1999) |
|  | 1.60 months: 0.17 ± 0.02 (14 %)  [n = 12] |  | (Allen et al., 1991) |
|  | 1.62 months: 0.19 ± 0.03 (15 %)  [n = 3] |  | (English, 1985; Ramsay et al., 2005) |
|  | 1.77 months: 0.16 ± 0.02 (14 %)  [n = 12] |  | (Allen et al., 1991) |
|  | 1.85 months: 0.18 ± 0.01 (4 %)  [n = 2] |  | (English, 1985; Aljazaf et al., 2003) |
|  | 1.90 months: 0.13  [n = 1] |  | (Butte et al., 1983) |
|  | 2.00 months: 0.18  [n = 1] |  | (Butte et al., 1983) |
|  | 2.08 months: 0.18  [n = 1] |  | (English, 1985) |
|  | 2.10 months: 0.12 ± 0.02 (15 %)  [n = 42] |  | (Butte et al., 1983) |
|  | 2.31 months: 0.16 ± 0.02 (14 %)  [n = 2] |  | (English, 1985; Aljazaf et al., 2003) |
|  | 2.54 months: 0.17  [n = 1] |  | (English, 1985) |
|  | 2.70 months: 0.18  [n = 1] |  | (Butte et al., 1983) |
|  | 2.72 months: 0.12 ± 0.02 (14 %)  [n = 10] |  | (Motil et al., 1997) |
|  | 2.77 months: 0.14 ± 0.03 (22 %)  [n = 4] |  | (English, 1985; Ramsay et al., 2005) |
|  | 2.80 months: 0.13  [n = 1] |  | (Butte et al., 1983) |
|  | 2.85 months: 0.14 ± 0.02 (16 %)  [n = 12] |  | (Allen et al., 1991) |
|  | 2.90 months: 0.11  [n = 1] |  | (Butte et al., 1983) |
|  | 2.96 months: 0.06 ± 0.01 (20 %)  [n = 57] |  | (Amatayakul et al., 1999) |
|  | 2.99 months: 0.11 ± 0.02 (17 %)  [n = 37] |  | (Butte et al., 1984) |
|  | 3.00 months: 0.14 ± 0.03 (19 %)  [n = 164] |  | (Butte et al., 1983; English, 1985; Nommsen et al., 1991; Alvear et al., 2000; Bhutta et al., 2000; Aljazaf et al., 2003; Ramsay et al., 2005; Bandara et al., 2015) |
|  | 3.10 months: 0.13 ± 0.003 (3 %)  [n = 2] |  | (Butte et al., 1983) |
|  | 3.20 months: 0.10 ± 0.02 (19 %)  [n = 20] |  | (Butte et al., 1983; Aljazaf et al., 2003; Khan et al., 2013a) |
|  | 3.30 months: 0.14  [n = 1] |  | (Butte et al., 1983) |
|  | 3.46 months: 0.18  [n = 1] |  | (Ramsay et al., 2005) |
|  | 3.48 months: 0.14 ± 0.01 (10 %)  [n = 24] |  | (Nielsen et al., 2011) |
|  | 3.50 months: 0.16  [n = 1] |  | (Butte et al., 1983) |
|  | 3.53 months: 0.13 ± 0.03 (21 %)  [n = 71] |  | (Kent et al., 2006) |
|  | 3.60 months: 0.14 ± 0.02 (15 %)  [n = 27] |  | (Butte et al., 1983; Nielsen et al., 2011) |
|  | 3.69 months: 0.13  [n = 1] |  | (Ramsay et al., 2005) |
|  | 3.70 months: 0.09  [n = 1] |  | (Butte et al., 1983) |
|  | 3.80 months: 0.12 ± 0.02 (13 %)  [n = 13] |  | (Butte et al., 1983; Allen et al., 1991) |
|  | 3.90 months: 0.15  [n = 1] |  | (Butte et al., 1983) |
|  | 3.91 months: 0.11 ± 0.02 (16 %)  [n = 41] |  | Butte et al., 1984 |
|  | 3.92 months: 0.16  [n = 1] |  | (Ramsay et al., 2005) |
|  | 4.00 months: 0.13 ± 0.03 (25 %)  [n = 128] |  | (Butte et al., 1983; Salmenperä et al., 1985; Haisma et al., 2003) |
|  | 4.08 months: 0.10 ± 0.03 (28 %)  [n = 10] |  | (Motil et al., 1997) |
|  | 4.15 months: 0.13  [n = 1] |  | (Ramsay et al., 2005) |
|  | 4.38 months: 0.11  [n = 1] |  | (Ramsay et al., 2005) |
|  | 4.62 months: 0.09 ± 0.03 (36 %)  [n = 2] |  | (Ramsay et al., 2005) |
|  | 4.79 months: 0.13 ± 0.02 (12 %)  [n = 12] |  | (Allen et al., 1991) |
|  | 5.00 months: 0.12 ± 0.03 (21 %)  [n = 16] |  | (Bandara et al., 2015) |
|  | 5.08 months: 0.10 ± 0.03 (29 %)  [n = 4] |  | (Ramsay et al., 2005) |
|  | 5.31 months: 0.11 ± 0.03 (23 %)  [n = 3] |  | (Ramsay et al., 2005) |
|  | 5.65 months: 0.11 ± 0.03 (24 %)  [n = 34] |  | (Motil et al., 1997; Nielsen et al., 2011) |
|  | 5.68 months: 0.13 ± 0.01 (11 %)  [n = 23] |  | (Nielsen et al., 2011) |
|  | 5.82 months: 0.11 ± 0.01 (13 %)  [n = 12] |  | (Allen et al., 1991) |
|  | 5.92 months: 0.04 ± 0.01 (27 %)  [n = 56] |  | (Amatayakul et al., 1999) |
|  | 6.00 months: 0.11 ± 0.02 (19 %)  [n = 163] |  | (Salmenperä et al., 1985; Nommsen et al., 1991; Alvear et al., 2000; Bhutta et al., 2000; Ramsay et al., 2005) |
|  | 6.73 months: 0.11 ± 0.01 (7 %)  [n = 10] |  | (Allen et al., 1991) |
|  | 7.38 months: 0.09  [n = 1] |  | (Aljazaf et al., 2003) |
|  | 7.76 months: 0.10 ± 0.01 (15 %)  [n = 10] |  | (Allen et al., 1991) |
|  | 8.71 months: 0.10 ± 0.01 (5 %)  [n = 10] |  | (Allen et al., 1991) |
|  | 8.88 months: 0.03 ± 0.01 (33 %)  [n = 52] |  | (Amatayakul et al., 1999) |
|  | 9.00 months: 0.08 ± 0.03 (33 %)  [n = 44] |  | (Salmenperä et al., 1985; Nommsen et al., 1991) |
|  | 11.00 months: 0.10 ± 0.02 (18 %)  [n = 10] |  | (Salmenperä et al., 1985) |
|  | 11.31 months: 0.08  [n = 1] |  | (Aljazaf et al., 2003) |
|  | 11.84 months: 0.02 ± 0.01 (49 %)  [n = 34] |  | (Amatayakul et al., 1999) |
|  | 12.00 months: 0.05 ± 0.02 (46 %)  [n = 21] |  | (Nommsen et al., 1991) |
| m-GFR (mL/min) | 0.03 months: 151.22 ± 33.89 (22 %)  [n = 34] | $\text{m-}\text{GFR (mL/min) = }\text{151.0285}\text{ }\text{-}\text{ }\text{57.1898 × PpT + 17.18}\text{5}\text{6 × }\text{PpT}^{\text{2}} \text{-}\text{ }\text{1.8479 × }\text{PpT}^{\text{3}}\text{+}\text{ }\text{0.0661 × }\text{PpT}^{\text{4}}\text{ }$ | (Lafayette et al., 1999; Hladunewich et al., 2004) |
|  | 0.46 months: 124.28 ± 33.76 (27 %)  [n = 22] |  | (Hladunewich et al., 2004) |
|  | 1.18 months: 94.50 ± 28.83 (31 %)  [n = 8] |  | (Sims and Krantz, 1958) |
|  | 1.98 months: 91.50 ± 16.20 (18 %)  [n = 8] |  | (Dunlop, 1981) |
|  | 2.30 months: 104.90 ± 15.28 (15 %)  [n = 10] |  | (Davison and Hytten, 1974) |
|  | 2.99 months: 96.72 ± 24.30 (25 %)  [n = 6] |  | (Sims and Krantz, 1958) |
|  | 4.00 months: 101.00 ± 39.60 (39 %)  [n = 11] |  | (Milne et al., 2002) |
|  | 4.61 months: 94.67 ± 19.28 (20 %)  [n = 21] |  | (Sturgiss et al., 1996) |
|  | 5.00 months: 92.00 ± 22.00 (24 %)  [n = 13] |  | (Moran et al., 2003) |
|  | 5.44 months: 103.66 ± 12.00 (12 %)  [n = 6] |  | (Sims and Krantz, 1958) |
|  | 5.99 months: 122.70 ± 18.03 (15 %)  [n = 11] |  | (Saxena et al., 2012) |
|  | 6.68 months: 128.00 ± 21.00 (16 %)  [n = 11] |  | (Ahmed et al., 2009) |
|  | 11.79 months: 92.20 ± 15.92 (17 %)  [n = 4] |  | (Sims and Krantz, 1958) |
| Breast volume (L) | 0.23 months: 1.56 ± 0.16 (10 %)  [n = 109] | $\text{Breast volume (L) =} \text{- 0.024 }\text{× PpT + 1.549}$ | (Hytten, 1954) |
|  | 0.57 months: 1.39 ± 0.05 (3 %)  [n = 12] |  | (Kent et al., 1999) |
|  | 1 month: 1.64 ± 0.36 (22 %)  [n = 7] |  | (Cox et al., 1999) |
|  | 1.60 months: 1.39 ± 0.10 (7 %)  [n = 12] |  | (Kent et al., 1999) |
|  | 2.40 months: 2.30  [n = 1] |  | (Daly et al., 1992) |
|  | 2.80 months: 1.33  [n = 1] |  | (Daly et al., 1992) |
|  | 3.63 months: 1.41 ± 0.10 (7 %)  [n = 10] |  | (Kent et al., 1999) |
|  | 5.83 months: 1.38 ± 0.12 (8 %)  [n = 12] |  | (Kent et al., 1999) |
|  | 6.00 months: 2.74  [n = 1] |  | (Daly et al., 1992) |
|  | 7.17 months: 1.39  [n = 1] |  | (Hale and Hartmann, 2017) |
|  | 7.25 months: 2.11  [n = 1] |  | (Daly et al., 1992) |
|  | 7.87 months: 1.37  [n = 1] |  | (Hale and Hartmann, 2017) |
|  | 8.00 months: 0.85  [n = 1] |  | (Arthur et al., 1989b) |
|  | 8.73 months: 1.30 ± 0.07 (5 %)  [n = 12] |  | (Kent et al., 1999) |
|  | 10.11 months: 1.42  [n = 1] |  | (Hale and Hartmann, 2017) |
|  | 11.00 months: 0.73  [n = 1] |  | (Arthur et al., 1989b) |
|  | 11.51 months: 1.37  [n = 1] |  | (Hale and Hartmann, 2017) |
|  | 11.82 months: 1.28 ± 0.05 (4 %)  [n = 10] |  | (Kent et al., 1999) |
| Maternal plasma volume (L) | 0.0011 months: 2.35 ± 0.31 (13 %)  [n = 18] | $\text{Plasma volume (L)} = \text{2.67}\text{ }\text{+}\text{ }\text{0.106 }\text{× (}\text{0.133}^{\text{PpT}}\text{)}$ | (Dieckmann and Wegner, 1934; Berlin et al., 1953) |
|  | 0.07 months: 2.87 ± 0.70 (24 %)  [n = 18] |  | (Pritchard and Rowland, 1964) |
|  | 0.10 months: 3.15  [n = 6] |  | (Dieckmann and Wegner, 1934) |
|  | 0.16 months: 3.08 ± 0.80 (26 %)  [n = 3] |  | (Dieckmann and Wegner, 1934) |
|  | 0.20 months: 2.88 ± 0.42 (15 %)  [n = 48] |  | (McLennan and Thouin, 1948; Bruinse et al., 1985) |
|  | 0.23 months: 2.53 ± 0.51 (20 %)  [n = 20] |  | (McLennan and Thouin, 1948) |
|  | 0.39 months: 3.13 ± 0.60 (20 %)  [n = 18] |  | (Dieckmann and Wegner, 1934) |
|  | 0.63 months: 3.77 ± 0.44 (20 %)  [n = 64] |  | (Dieckmann and Wegner, 1934) |
|  | 0.82 months: 2.93  [n = 1] |  | (Dieckmann and Wegner, 1934) |
|  | 1.38 months: 2.38 ± 0.44 (19 %)  [n = 64] |  | (Berlin et al., 1953; Bruinse et al., 1985; Rajalakshmi and Raman, 1985) |
|  | 1.64 months: 2.63 ± 0.42 (16 %)  [n = 84] |  | (Paintin, 1962; Hytten and Paintin, 1963; Butler, 1968; Pirani et al., 1973) |
|  | 1.85 months: 2.47 ± 0.62 (25 %)  [n = 29] |  | (Dieckmann and Wegner, 1934; Abudu and Sofola, 1988) |
|  | 2.83 months: 2.48 ± 0.36 (15 %)  [n = 159] |  | (Gibson, 1973; Hunyor et al., 1982; Whittaker and Lind, 1993; Pivarnik et al., 1994; Whittaker et al., 1996) |
|  | 3.23 months: 2.80 ± 0.60 (21 %)  [n = 52] |  | (Hunyor, 1984) |
|  | 3.50 months: 2.25  [n = 26] |  | (Salas et al., 1993) |
|  | 4.00 months: 2.45 ± 0.55 (22 %)  [n = 54] |  | (Vargas et al., 2007) |
|  | 5.00 months: 2.34 ± 0.23 (10 %)  [n = 24] |  | (Taylor and Lind, 1979) |
|  | 6.00 months: 2.52 ± 0.25 (10 %)  [n = 37] |  | (Bruinse et al., 1985) |
| Maternal haematocrit (%) | 0.03 months: 30.78 ± 3.62 (12 %)  [n = 9019] | $\text{Haematocrit (\%) = }\text{31.17 + }\frac{\text{(38.74 - 31.17) × }\text{PpT}^{\text{2.49}}}{\text{0.133}^{\text{2.49}}\text{+ }\text{PpT}^{\text{2.49}}}$ | (Taylor et al., 1981; Ragsdale et al., 2022) |
|  | 0.07 months: 33.41 ± 4.32 (13 %)  [n = 22] |  | (Pritchard and Rowland, 1964; Taylor et al., 1981) |
|  | 0.12 months: 33.88 ± 4.00 (12 %)  [n = 32] |  | (Taylor et al., 1981) |
|  | 0.16 months: 35.04 ± 3.60 (10 %)  [n = 16] |  | (Taylor et al., 1981) |
|  | 0.20 months: 35.82 ± 3.30 (9 %)  [n = 16] |  | (Taylor et al., 1981) |
|  | 0.23 months: 39.62 ± 3.30 (8 %)  [n = 20] |  | (McLennan and Thouin, 1948) |
|  | 0.46 months: 37.60 ± 1.94 (5 %)  [n = 15] |  | (Robson et al., 1987b) |
|  | 0.58 months: 38.00 ± 2.00 (5 %)  [n = 9] |  | (Pond et al., 1985) |
|  | 0.88 months: 38.70 ± 3.61 (9 %)  [n = 9003] |  | (Ragsdale et al., 2022) |
|  | 1.29 months: 39.74± 3.42 (9 %)  [n = 340] |  | (Darby et al., 1953; Taylor et al., 1981; Bruinse et al., 1985; Robson et al., 1987b) |
|  | 1.61 months: 40.30 ± 3.05 (8 %)  [n = 1295] |  | (Darby et al., 1953; Taylor and Lind, 1979; Frederiksen et al., 1986) |
|  | 1.87 months: 39.11 ± 2.22 (6 %)  [n = 238] |  | (Pond et al., 1985; Sala et al., 1995; Milman et al., 2000; Milman et al., 2007) |
|  | 2.77 months: 39.32 ± 1.68 (4 %)  [n = 90] |  | (Robson et al., 1987b; Pivarnik et al., 1994; Whittaker et al., 1996) |
|  | 3.50 months: 36.10 ± 2.55 (7 %)  [n = 26] |  | (Salas et al., 1993) |
|  | 5.00 months: 39.36 ± 2.33 (6 %)  [n = 24] |  | (Taylor and Lind, 1979) |
|  | 6.00 months: 39.34 ± 2.40 (6 %)  [n = 16] |  | (Taylor et al., 1981) |
| m-CO (L/h) | 0.03 months: 360.50 ± 61.02 (17 %)  [n = 141] | $\text{m-CO (L/h) =} \text{98.8 }\text{e}^{\text{(-3.33 × PpT)}}\text{ + 304.4 }\text{e}^{\text{(-0.00096 × PpT)}}$ | (Robson et al., 1989; Hunter and Robson, 1992; Timokhina et al., 2019; Ambrožič et al., 2020) |
|  | 0.04 months: 408.00 ± 96.00 (24 %)  [n = 123] |  | (Ram et al., 2017; Lavie et al., 2018) |
|  | 0.07 months: 378.12 ± 85.05 (22 %)  [n = 80] |  | (Robson et al., 1987a; Robson et al., 1989; Hunter and Robson, 1992; San-Frutos et al., 2005; Burlingame et al., 2013) |
|  | 0.08 months: 408.00 ± 84.00 (21 %)  [n = 120] |  | (Ram et al., 2017; Lavie et al., 2018) |
|  | 0.10 months: 412.00 ± 13.00 (3 %)  [n = 55] |  | (Timokhina et al., 2019) |
|  | 0.13 months: 349.00 ± 56.00 (16 %)  [n = 42] |  | (Milsom et al., 1988; Ambrožič et al., 2020) |
|  | 0.16 months: 365.00 ± 13.00 (4 %)  [n = 55] |  | (Timokhina et al., 2019) |
|  | 0.20 months: 360.00 ± 27.00 (8 %)  [n = 36] |  | (Robson et al., 1989; Hunter and Robson, 1992) |
|  | 0.30 months: 326.00 ± 11.00 (3 %)  [n = 55] |  | (Timokhina et al., 2019) |
|  | 0.33 months: 323.00 ± 26.00 (8 %)  [n = 40] |  | (Robson et al., 1989; Hunter and Robson, 1992) |
|  | 0.46 months: 308.15 ± 20.09 (7 %)  [n = 99] |  | (Robson et al., 1989; Hunter and Robson, 1992; Timokhina et al., 2019) |
|  | 0.47 months: 323.60 ± 36.03 (11 %)  [n = 24] |  | (Robson et al., 1987a; Robson et al., 1987b) |
|  | 0.69 months: 348.00 ± 75.00 (22 %)  [n = 34] |  | (Geva et al., 1997) |
|  | 1.38 months: 302.42 ± 65.80 (22 %)  [n = 464] |  | (Robson et al., 1987b; Easterling et al., 1990; Mabie et al., 1994; Hennessy et al., 1996; Van Oppen et al., 1996; Gilson et al., 1997; Ogueh et al., 2009; Andreas et al., 2016; Masini et al., 2019) |
|  | 1.62 months: 276.00 ± 48.00 (17 %)  [n = 11] |  | (Ueland and Hansen, 1969) |
|  | 1.70 months: 276.00 ± 48.00 (17 %)  [n = 37] |  | (Mesa et al., 1999) |
|  | 1.85 months: 360.00 ± 60.00 (17 %)  [n = 14] |  | (Poppas et al., 1997) |
|  | 1.94 months: 252.00 ± 18.00 (7 %)  [n = 16] |  | (Mashini et al., 1987) |
|  | 2.00 months: 323.24 ± 64.51 (20 %)  [n = 73] |  | (San-Frutos et al., 2005; Timokhina et al., 2019) |
|  | 2.08 months: 301.11 ± 38.79 (13 %)  [n = 46] |  | (Katz et al., 1978; Desai et al., 2004) |
|  | 2.31 months: 297.60 ± 61.80 (21 %)  [n = 63] |  | (Bamfo et al., 2007) |
|  | 2.77 months: 334.93 ± 69.91 (21 %)  [n = 174] |  | (Robson et al., 1987b; Clark et al., 1989; Mabie et al., 1994; Van Oppen et al., 1996; Clapp III and Capeless, 1997; D’Silva et al., 2013; Nelson et al., 2015) |
|  | 3.00 months: 310.95 ± 59.16 (19 %)  [n = 75] |  | (Milsom et al., 1988; Duvekot et al., 1993; Geva et al., 1997; Tyldum et al., 2012) |
|  | 3.09 months: 342.00 ± 66.97 (20 %)  [n = 34] |  | (Carpenter et al., 2015) |
|  | 3.53 months: 225.00 ± 39.23 (17 %)  [n = 68] |  | (Ducas et al., 2014) |
|  | 3.69 months: 300.00 ± 102.00 (34 %)  [n = 11] |  | (Airaksinen et al., 1986) |
|  | 4.00 months: 258.00 ± 24.00 (9 %)  [n = 31] |  | (Savu et al., 2012) |
|  | 5.00 months: 336.00 ± 72.00 (21 %)  [n = 16] |  | (Rang et al., 2007) |
|  | 5.54 months: 309.38 ± 64.79 (21 %)  [n = 44] |  | (Robson et al., 1987b; Clapp III and Capeless, 1997) |
|  | 6.00 months: 303.47 ± 58.78 (19 %)  [n = 478] |  | (Estensen et al., 2013; Andreas et al., 2016; Aguilera et al., 2020) |
|  | 7.38 months: 252.00 ± 30.00 (12 %)  [n = 17] |  | (Di Martino et al., 2023) |
|  | 12.00 months: 311.40 ± 69.01 (22 %)  [n = 30] |  | (Clapp III and Capeless, 1997) |
| m-HSA (g/L) | At delivery: 35.06 ± 2.95 (8 %)  [n = 329] | $\text{m-H}\text{SA (g/L) = }\text{32.7+}\left( \frac{\text{12.15}}{\text{1 + }\text{e}^{\text{(-7.16 }\text{× }\text{(PpT - 0.866))}}} \right)$ | (Herngren et al., 1983; Klajnbard et al., 2010) |
|  | 0.0002 months: 36.00 ± 2.00 (6 %)  [n = 8] |  | (Echizen et al., 1990) |
|  | 0.03 months: 31.85 ± 3.17 (10 %)  [n = 347] |  | (Paaby, 1960; Larijani et al., 1990; Klajnbard et al., 2010) |
|  | 0.05 months: 27.72 ± 1.77 (6 %)  [n = 5] |  | (Bardy et al., 1990) |
|  | 0.06 months: 31.38 ± 4.22 (13 %)  [n = 247] |  | (Larijani et al., 1990; Klajnbard et al., 2010; Lisowska-Myjak et al., 2021) |
|  | 0.10 months: 37.05 ± 1.14 (3 %)  [n = 43] |  | (Paaby, 1960; Dean et al., 1980) |
|  | 0.11 months: 24.74 ± 2.33 (9 %)  [n = 5] |  | (Bardy et al., 1990) |
|  | 0.16 months: 30.60  [n = 2] |  | (Paaby, 1960) |
|  | 0.18 months: 25.49 ± 1.12 (4 %)  [n = 5] |  | (Bardy et al., 1990) |
|  | 0.23 months: 34.99 ± 2.66 (8 %)  [n = 72] |  | (Paaby, 1960; Herngren et al., 1983) |
|  | 0.24 months: 25.86 ± 2.14 (8 %)  [n = 5] |  | (Bardy et al., 1990) |
|  | 0.26 months: 33.00  [n = 7] |  | (Paaby, 1960) |
|  | 0.42 months: 36.50  [n = 5] |  | (Paaby, 1960) |
|  | 0.45 months: 35.35 ± 1.21 (3 %)  [n = 5] |  | (Bardy et al., 1990) |
|  | 0.68 months: 35.00  [n = 17] |  | (Paaby, 1960) |
|  | 0.90 months: 37.60  [n = 3] |  | (Paaby, 1960) |
|  | 0.97 months: 37.00  [n = 13] |  | (Paaby, 1960) |
|  | 0.98 months: 47.00 ± 4.00 (9 %)  [n = 6] |  | (Echizen et al., 1990) |
|  | 1.13 months: 41.10  [n = 16] |  | (Paaby, 1960) |
|  | 1.16 months: 39.80  [n = 8] |  | (Paaby, 1960) |
|  | 1.22 months: 45.47 ± 3.52 (8 %)  [n = 127] |  | (Darby et al., 1953) |
|  | 1.35 months: 48.32 ± 3.76 (8 %)  [n = 34] |  | (Dean et al., 1980; Wright et al., 1987) |
|  | 1.39 months: 38.50 ± 5.00 (13 %)  [n = 19] |  | (Paaby, 1960) |
|  | 1.56 months: 46.00 ± 2.29 (5 %)  [n = 402] |  | (Darby et al., 1953) |
|  | 1.58 months: 45.89 ± 3.05 (7 %)  [n = 138] |  | (Darby et al., 1953; Frederiksen et al., 1986) |
|  | 2.32 months: 33.70 ± 2.50 (7 %)  [n = 51] |  | (Larsson et al., 2008) |
|  | 2.71 months: 45.60 ± 5.43 (12 %)  [n = 12] |  | (Whittaker and Lind, 1993) |
|  | 5.88 months: 42.00 ± 2.00 (5 %)  [n = 5] |  | (Frederiksen et al., 1986) |
| m-AGP (g/L) | 0.0002 months: 0.51 ± 0.11 (23 %)  [n = 18] | If PpT <= 1 then  ${\text{m-AGP (g/L) =}\text{ }\text{e}}^{\text{(-1.277 }\text{× PpT) }}\text{-}\text{ }\text{e}^{\text{(}\text{-}\text{ 6.749 × PpT)}}\text{ + 0.6}$  Else  $\text{m-AGP (g/L) = 0.016 }\text{× PpT + 0.90}$ | (Herngren et al., 1983; Echizen et al., 1990) |
|  | 0.03 months: 0.90 ± 0.18 (20 %)  [n = 22] |  | (Larijani et al., 1990) |
|  | 0.05 months: 0.67 ± 0.22 (33 %)  [n = 33] |  | (Fleishaker et al., 1989; Lisowska-Myjak et al., 2021) |
|  | 0.06 months: 1.00 ± 0.31 (31 %)  [n = 30] |  | (Larijani et al., 1990) |
|  | 0.11 months: 1.10 ± 0.35 (31 %)  [n = 5] |  | (Fleishaker et al., 1989) |
|  | 0.18 months: 1.24 ± 0.31 (25 %)  [n = 5] |  | (Fleishaker et al., 1989) |
|  | 0.23 months: 1.12 ± 0.23 (20 %)  [n = 6] |  | (Herngren et al., 1983) |
|  | 0.24 months: 1.28 ± 0.26 (20 %)  [n = 5] |  | (Fleishaker et al., 1989) |
|  | 0.26 months: 0.87 ± 0.04 (5 %)  [n = 16] |  | (Honda et al., 1990) |
|  | 0.45 months: 1.14 ± 0.35 (31 %)  [n = 5] |  | (Fleishaker et al., 1989) |
|  | 0.98 months: 0.89 ± 0.20 (22 %)  [n = 6] |  | (Echizen et al., 1990) |

^a^ Values are expressed as mean ± SD (coefficient of variation %) [number of individuals]. **m-AGP** = maternal alpha-1-acid glycoprotein, m-**m-HSA** = maternal human serum albumin, **m-GFR** = glomerular filtration rate, **m-CO** = maternal cardiac output, **PpT** = postpartum age in months.

# Supplementary Figures


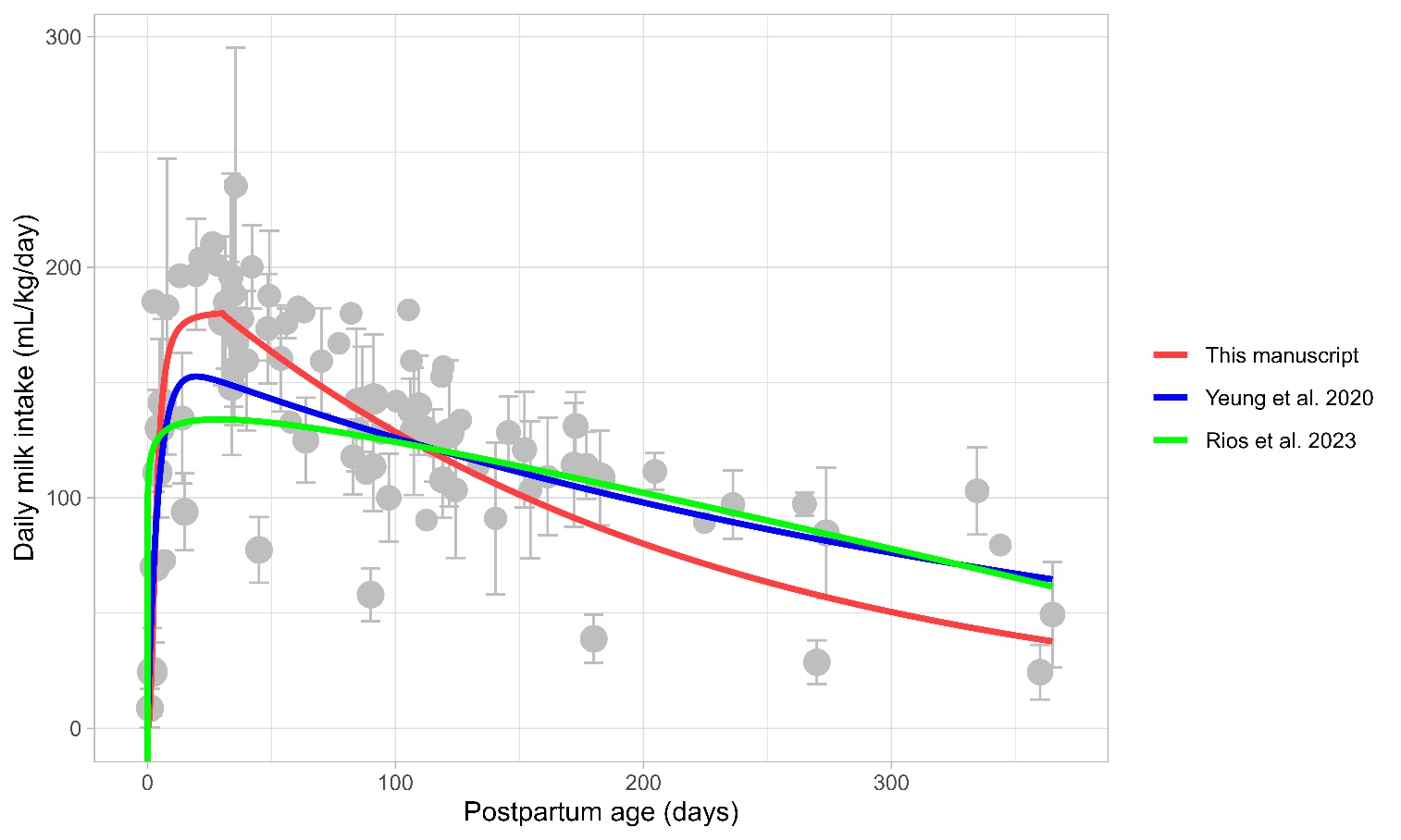


**Supplementary Figure 1.** Dynamic functions describing the change in daily milk intake in function of postpartum age. Grey dots depict the observed data, error bars indicate standard deviations, and the dot sizes correspond to the number of samples. The equations presented are from this manuscript, (Yeung et al., 2020) and (Rios-Leyvraz and Yao, 2023).

# References

Abudu, O.O., and Sofola, O.A. (1988). Intravascular volume expansion and fetal outcome in pregnant Nigerians with hemoglobin SS and SC. *Journal of the National Medical Association* 80(8)**,** 906.

Agne-Djigo, A., Kwadjode, K.M., Idohou-Dossou, N., Diouf, A., Guiro, A.T., and Wade, S. (2013). Energy intake from human milk covers the requirement of 6-month-old Senegalese exclusively breast-fed infants. *British journal of nutrition* 110(10)**,** 1849-1855.

Aguilera, J., Sanchez Sierra, A., Abdel Azim, S., Georgiopoulos, G., Nicolaides, K., and Charakida, M. (2020). Maternal cardiac function in gestational diabetes mellitus at 35–36 weeks' gestation and 6 months postpartum. *Ultrasound in Obstetrics & Gynecology* 56(2)**,** 247-254.

Ahmed, S.B., Bentley-Lewis, R., Hollenberg, N.K., Graves, S.W., and Seely, E.W. (2009). A comparison of prediction equations for estimating glomerular filtration rate in pregnancy. *Hypertension in Pregnancy* 28(3)**,** 243-255.

Airaksinen, K., Ikäheimo, M., Salmela, P., Kirkinen, P., Linnaluoto, M., and Takkunen, J. (1986). Impaired cardiac adjustment to pregnancy in type I diabetes. *Diabetes care* 9(4)**,** 376-383.

Aljazaf, K., Hale, T.W., Ilett, K.F., Hartmann, P.E., Mitoulas, L.R., Kristensen, J.H., et al. (2003). Pseudoephedrine: effects on milk production in women and estimation of infant exposure via breastmilk. *British journal of clinical pharmacology* 56(1)**,** 18-24.

Allen, J.C., Keller, R.P., Archer, P., and Neville, M.C. (1991). Studies in human lactation: milk composition and daily secretion rates of macronutrients in the first year of lactation. *The American journal of clinical nutrition* 54(1)**,** 69-80.

Alvear, J., Salazar, G., Berlanga, R., and de los Santos, M. (2000). Breast feeding and growth in a group of selected 0 to 24 months infants.

Amatayakul, K., Wongsawasdi, L., Mangklabruks, A., Tansuhaj, A., Ruckphaopunt, S., Chiowanich, P., et al. (1999). Effects of parity on breastfeeding: a study in the rural setting in northern Thailand. *Journal of Human Lactation* 15(2)**,** 121-124.

Ambrožič, J., Lučovnik, M., Prokšelj, K., Toplišek, J., and Cvijić, M. (2020). Dynamic changes in cardiac function before and early postdelivery in women with severe preeclampsia. *Journal of hypertension* 38(7)**,** 1367-1374.

Anderson, G.H., Atkinson, S.A., and Bryan, M.H. (1981). Energy and macronutrient content of human milk during early lactation from mothers giving birth prematurely and at term. *The American Journal of Clinical Nutrition* 34(2)**,** 258-265.

Andreas, M., Kuessel, L., Kastl, S.P., Wirth, S., Gruber, K., Rhomberg, F., et al. (2016). Bioimpedance cardiography in pregnancy: A longitudinal cohort study on hemodynamic pattern and outcome. *BMC pregnancy and childbirth* 16**,** 1-9.

Ansell, C., Moore, A., and Barrie, H. (1977). Electrolyte and pH changes in human milk. *Pediatric research* 11(12)**,** 1177-1179.

Arcus-Arth, A., Krowech, G., and Zeise, L. (2005). Breast milk and lipid intake distributions for assessing cumulative exposure and risk. *Journal of Exposure Science & Environmental Epidemiology* 15(4)**,** 357-365.

Arthur, P.G., Smith, M., and Hartmann, P.E. (1989a). Milk lactose, citrate, and glucose as markers of lactogenesis in normal and diabetic women. *Journal of Pediatric Gastroenterology and Nutrition* 9(4)**,** 488-496.

Arthur, P.G., Spruce, J., Hartmann, P.E., and Jones, T.J. (1989b). Measuring short‐term rates of milk synthesis in breast‐feeding mothers. *Quarterly Journal of Experimental Physiology: Translation and Integration* 74(4)**,** 419-428.

Bamfo, J.E., Kametas, N.A., Nicolaides, K.H., and Chambers, J.B. (2007). Maternal left ventricular diastolic and systolic long-axis function during normal pregnancy. *European Journal of Echocardiography* 8(5)**,** 360-368.

Bandara, T., Hettiarachchi, M., Liyanage, C., Amarasena, S., and Wong, W.W. (2015). The deuterium oxide-to-the-mother method documents adequate breast-milk intake among Sri Lankan infants. *The Journal of Nutrition* 145(6)**,** 1325-1329.

Bardy, A., Hiilesmaa, V., Teramo, K., and Neuvonen, P. (1990). Protein binding of antiepileptic drugs during pregnancy, labor, and puerperium. *Therapeutic drug monitoring* 12(1)**,** 40-46.

Berlin, N., Goetsch, C., Hyde, G., and Parsons, R. (1953). The blood volume in pregnancy as determined by P32 labeled red blood cells.

Bhutta, Z.A., Weaver, L., and Preston, T. (2000). Isotopic evaluation of breast milk intake, energy metabolism, growth and body composition of exclusively breast fed infants in Pakistan.

Bruinse, H.W., van den Berg, H., and Haspels, A.A. (1985). Smoking and its effect on maternal plasma volume during and after normal pregnancy. *European Journal of Obstetrics & Gynecology and Reproductive Biology* 20(4)**,** 215-219.

Burlingame, J., Ohana, P., Aaronoff, M., and Seto, T. (2013). Noninvasive cardiac monitoring in pregnancy: impedance cardiography versus echocardiography. *Journal of Perinatology* 33(9)**,** 675-680.

Butler, E.B. (1968). Effect of iron and folic acid on red cell and plasma volume in pregnancy. *J Obstet Gynaecol Br Commonw* 75(5)**,** 497-510. doi: 10.1111/j.1471-0528.1968.tb00153.x.

Butte, N., Smith, E., and Garza, C. (1990a). Energy utilization of breast-fed and formula-fed infants. *The American journal of clinical nutrition* 51(3)**,** 350-358.

Butte, N.F., Garza, C., Smith, E., and Nichols, B.L. (1983). Evaluation of the deuterium dilution technique against the test-weighing procedure for the determination of breast milk intake. *The American Journal of Clinical Nutrition* 37(6)**,** 996-1003.

Butte, N.F., Garza, C., Smith, E.B., and Nichols, B.L. (1984). Human milk intake and growth in exclusively breast-fed infants. *The Journal of pediatrics* 104(2)**,** 187-195.

Butte, N.F., Wong, W.W., Ferlic, L., Smith, E.O.B., Klein, P.D., and Garza, C. (1990b). Energy expenditure and deposition of breast-fed and formula-fed infants during early infancy. *Pediatric research* 28(6)**,** 631-640.

Butts, C.A., Hedderley, D.I., Herath, T.D., Paturi, G., Glyn-Jones, S., Wiens, F., et al. (2018). Human milk composition and dietary intakes of breastfeeding women of different ethnicity from the Manawatu-Wanganui region of New Zealand. *Nutrients* 10(9)**,** 1231.

Bzikowska-Jura, A., Czerwonogrodzka-Senczyna, A., Olędzka, G., Szostak-Węgierek, D., Weker, H., and Wesołowska, A. (2018). Maternal nutrition and body composition during breastfeeding: association with human milk composition. *Nutrients* 10(10)**,** 1379.

Bzikowska-Jura, A., Sobieraj, P., Szostak-Wegierek, D., and Wesolowska, A. (2020). Impact of Infant and Maternal Factors on Energy and Macronutrient Composition of Human Milk. *Nutrients* 12(9). doi: 10.3390/nu12092591.

Caldeo, V., Downey, E., O'Shea, C.A., Affolter, M., Volger, S., Courtet-Compondu, M.C., et al. (2021). Protein levels and protease activity in milk from mothers of pre-term infants: A prospective longitudinal study of human milk macronutrient composition. *Clin Nutr* 40(5)**,** 3567-3577. doi: 10.1016/j.clnu.2020.12.013.

Carey, G.B., Quinn, T.J., and Goodwin, S.E. (1997). Breast milk composition after exercise of different intensities. *Journal of Human Lactation* 13(2)**,** 115-120.

Carpenter, R.E., Emery, S.J., Uzun, O., D’Silva, L.A., and Lewis, M.J. (2015). Influence of antenatal physical exercise on haemodynamics in pregnant women: a flexible randomisation approach. *BMC Pregnancy and Childbirth* 15**,** 1-15.

Casey, C.E., Neifert, M.R., Seacat, J.M., and Neville, M.C. (1986). Nutrient intake by breast-fed infants during the first five days after birth. *American journal of diseases of children* 140(9)**,** 933-936.

Cho, J., Ahn, H.Y., Ahn, S., Lee, M.S., and Hur, M.H. (2012). Effects of Oketani Breast Massage on Breast Pain, the Breast Milk pH of Mothers, and the Sucking Speed of Neonates. *Korean J Women Health Nurs* 18(2)**,** 149-158. doi: 10.4069/kjwhn.2012.18.2.149.

Christie, A., Darke, S., Paul, A., Wharton, B., and Widdowson, E. (1977). "The Composition of Mature Hu man Milk. Department of Health and Social Security, Report on Health and Social Subjects, No. 12". HMSO, London).

Clapp III, J.F., and Capeless, E. (1997). Cardiovascular function before, during, and after the first and subsequent pregnancies. *The American journal of cardiology* 80(11)**,** 1469-1473.

Clark, R.M., Ferris, A.M., Fey, M., Brown, P.B., Hundrieser, K.E., and Jensen, R.G. (1982). Changes in the lipids of human milk from 2 to 16 weeks postpartum. *Journal of Pediatric Gastroenterology and Nutrition* 1(3)**,** 311-316.

Clark, S.L., Cotton, D.B., Lee, W., Bishop, C., Hill, T., Southwick, J., et al. (1989). Central hemodynamic assessment of normal term pregnancy. *American journal of obstetrics and gynecology* 161(6)**,** 1439-1442.

Corvaglia, L., Battistini, B., Paoletti, V., Aceti, A., Capretti, M.G., and Faldella, G. (2008). Near-infrared reflectance analysis to evaluate the nitrogen and fat content of human milk in neonatal intensive care units. *Archives of Disease in Childhood-Fetal and Neonatal Edition* 93(5)**,** F372-F375.

Cox, D.B., Kent, J.C., Casey, T.M., Owens, R.A., and Hartmann, P.E. (1999). Breast growth and the urinary excretion of lactose during human pregnancy and early lactation: endocrine relationships. *Experimental Physiology* 84(2)**,** 421-434.

Cox, D.B., Owens, R.A., and Hartmann, P.E. (1996). Blood and milk prolactin and the rate of milk synthesis in women. *Experimental Physiology: Translation and Integration* 81(6)**,** 1007-1020.

Cregan, M.D., Mitoulas, L.R., and Hartmann, P.E. (2002). Milk prolactin, feed volume and duration between feeds in women breastfeeding their full-term infants over a 24 h period. *Experimental Physiology* 87(2)**,** 207-214.

Czosnykowska-Łukacka, M., Królak-Olejnik, B., and Orczyk-Pawiłowicz, M. (2018). Breast milk macronutrient components in prolonged lactation. *Nutrients* 10(12)**,** 1893.

D’Silva, L.A., Davies, R.E., Emery, S.J., and Lewis, M.J. (2013). Influence of somatic state on cardiovascular measurements in pregnancy. *Physiological Measurement* 35(1)**,** 15.

Daly, S., Di Rosso, A., Owens, R.A., and Hartmann, P.E. (1993). Degree of breast emptying explains changes in the fat content, but not fatty acid composition, of human milk. *Experimental Physiology: Translation and Integration* 78(6)**,** 741-755.

Daly, S., Kent, J., Huynh, D., Owens, R., Alexander, B., Ng, K., et al. (1992). The determination of short‐term breast volume changes and the rate of synthesis of human milk using computerized breast measurement. *Experimental Physiology: Translation and Integration* 77(1)**,** 79-87.

Darby, W.J., McGanity, W.J., Martin, M.P., Bridgforth, E., Densen, P.M., Kaser, M.M., et al. (1953). The Vanderbilt cooperative study of maternal and infant nutrition. 4. Dietary, laboratory and physical findings in 2,129 delivered pregnancies.

Davison, J., and Hytten, F. (1974). Glomerular filtration during and after pregnancy. *BJOG: An International Journal of Obstetrics & Gynaecology* 81(8)**,** 588-595.

de Carvalho, M., Robertson, S., Merkatz, R., and Klaus, M. (1982). Milk intake and frequency of feeding in breast fed infants. *Early human development* 7(2)**,** 155-163.

Dean, M., Stock, B., Patterson, R.J., and Levy, G. (1980). Serum protein binding of drugs during and after pregnancy in humans. *Clinical Pharmacology & Therapeutics* 28(2)**,** 253-261.

Desai, D.K., Moodley, J., and Naidoo, D.P. (2004). Echocardiographic assessment of cardiovascular hemodynamics in normal pregnancy. *Obstetrics & Gynecology* 104(1)**,** 20-29.

Dewey, K.G., Finley, D.A., and Lönnerdal, B. (1984). Breast Milk Volume and Composition During Late Lactation (7–20 Months. *Journal of pediatric gastroenterology and nutrition* 3(5)**,** 713-720.

Dewey, K.G., and Lönnerdal, B. (1983). Milk and nutrient intake of breast-fed infants from 1 to 6 months: relation to growth and fatness. *Journal of Pediatric Gastroenterology and Nutrition* 2(3)**,** 497-506.

Di Martino, D.D., Stampalija, T., Zullino, S., Fusè, F., Garbin, M., Parasiliti, M., et al. (2023). Maternal hemodynamic profile during pregnancy and in the post-partum in hypertensive disorders of pregnancy and fetal growth restriction. *American Journal of Obstetrics & Gynecology MFM* 5(3)**,** 100841.

Dieckmann, W.J., and Wegner, C.R. (1934). The blood in normal pregnancy: I. Blood and plasma volumes. *Archives of Internal Medicine* 53(1)**,** 71-86.

Dollberg, S., Lahav, S., and Mimouni, F.B. (2001). A comparison of intakes of breast-fed and bottle-fed infants during the first two days of life. *Journal of the American College of Nutrition* 20(3)**,** 209-211.

Ducas, R.A., Elliott, J.E., Melnyk, S.F., Premecz, S., Cleverley, K., Wtorek, P., et al. (2014). Cardiovascular magnetic resonance in pregnancy: insights from the cardiac hemodynamic imaging and remodeling in pregnancy (CHIRP) study. *Journal of Cardiovascular Magnetic Resonance* 16(1)**,** 1.

Dunlop, W. (1981). Serial changes in renal haemodynamics during normal human pregnancy. *BJOG: An International Journal of Obstetrics & Gynaecology* 88(1)**,** 1-9.

Duvekot, J.J., Cheriex, E.C., Pieters, F.A., Menheere, P.P., and Peeters, L.L. (1993). Early pregnancy changes in hemodynamics and volume homeostasis are consecutive adjustments triggered by a primary fall in systemic vascular tone. *American journal of obstetrics and gynecology* 169(6)**,** 1382-1392.

Easterling, T.R., Benedetti, T.J., Schmuker, B.C., and Millard, S.P. (1990). Maternal hemodynamics in normal and preeclamptic pregnancies: a longitudinal study. *Obstetrics & Gynecology* 76(6)**,** 1061-1069.

Echizen, H., Nakura, M., Saotome, T., Minoura, S., and Ishizaki, T. (1990). Plasma protein binding of disopyramide in pregnant and postpartum women, and in neonates and their mothers. *British journal of clinical pharmacology* 29(4)**,** 423-430.

Emmett, P.M., and Rogers, I.S. (1997). Properties of human milk and their relationship with maternal nutrition. *Early Hum Dev* 49 Suppl**,** S7-28. doi: 10.1016/s0378-3782(97)00051-0.

English, R. (1985). Breast-milk production and energy exchange in human lactation. *British journal of nutrition* 53(3)**,** 459-466.

Erickson, T., Gill, G., and Chan, G. (2013). The effects of acidification on human milk’s cellular and nutritional content. *Journal of Perinatology* 33(5)**,** 371-373.

Estensen, M., Beitnes, J., Grindheim, G., Aaberge, L., Smiseth, O., Henriksen, T., et al. (2013). Altered maternal left ventricular contractility and function during normal pregnancy. *Ultrasound in Obstetrics & Gynecology* 41(6)**,** 659-666.

Evans, K., Evans, R., Royal, R., Esterman, A., and James, S. (2003). Effect of caesarean section on breast milk transfer to the normal term newborn over the first week of life. *Archives of Disease in Childhood-Fetal and Neonatal Edition* 88(5)**,** F380-F382.

Fleishaker, J.C., Desai, N., and McNamara, P.J. (1989). Possible effect of lactational period on the milk-to-plasma drug concentration ratio in lactating women: results of an in vitro evaluation. *Journal of pharmaceutical sciences* 78(2)**,** 137-141.

Frederiksen, M.C., Ruo, T.I., Chow, M.J., and Atkinson Jr, A.J. (1986). Theophylline pharmacokinetics in pregnancy. *Clinical Pharmacology & Therapeutics* 40(3)**,** 321-328.

Garza, C., Johnson, C.A., Smith, E., and Nichols, B.L. (1983). Changes in the nutrient composition of human milk during gradual weaning. *The American journal of clinical nutrition* 37(1)**,** 61-65.

Geva, T., Mauer, M.B., Strikera, L., Kirshon, B., and Pivarnik, J.M. (1997). Effects of physiologic load of pregnancy on left ventricular contractility and remodeling. *American heart journal* 133(1)**,** 53-59.

Gibson, H.M. (1973). Plasma volume and glomerular filtration rate in pregnancy and their relation to differences in fetal growth. *BJOG: An International Journal of Obstetrics & Gynaecology* 80(12)**,** 1067-1074.

Gilson, G.J., Samaan, S., Crawford, M.H., Quails, C.R., and Curet, L.B. (1997). Changes in hemodynamics, ventricular remodeling, and ventricular contractility during normal pregnancy: a longitudinal study. *Obstetrics & Gynecology* 89(6)**,** 957-962.

Glew, R.H., Wold, R.S., Corl, B., Calvin, C.D., and Vanderjagt, D.J. (2011). Low docosahexaenoic acid in the diet and milk of American Indian women in New Mexico. *Journal of the American Dietetic Association* 111(5)**,** 744-748.

Goldfarb, M.F., and Savadove, M.S. (1991). Creamatocrit and pH measurements of human milk. *J Pediatr Gastroenterol Nutr* 12(1)**,** 142-143.

Gross, S.J., David, R.J., Bauman, L., and Tomarelli, R. (1980). Nutritional composition of milk produced by mothers delivering preterm. *The Journal of pediatrics* 96(4)**,** 641-644.

Grote, V., Verduci, E., Scaglioni, S., Vecchi, F., Contarini, G., Giovannini, M., et al. (2016). Breast milk composition and infant nutrient intakes during the first 12 months of life. *European journal of clinical nutrition* 70(2)**,** 250-256.

Haisma, H., Coward, W., Albernaz, E., Visser, G., Wells, J.C.K., Wright, A., et al. (2003). Breast milk and energy intake in exclusively, predominantly, and partially breast-fed infants. *European journal of clinical nutrition* 57(12)**,** 1633-1642.

Hale, T., and Hartmann, P. (2017). *Textbook of Human Lactation.*

Hamosh, M., Ellis, L.A., Pollock, D.R., Henderson, T.R., and Hamosh, P. (1996). Breastfeeding and the working mother: effect of time and temperature of short-term storage on proteolysis, lipolysis, and bacterial growth in milk. *Pediatrics* 97(4)**,** 492-498.

Handa, D., Ahrabi, A., Codipilly, C., Shah, S., Ruff, S., Potak, D., et al. (2014). Do thawing and warming affect the integrity of human milk? *Journal of Perinatology* 34(11)**,** 863-866.

Harrison, V.C., and Peat, G. (1972). Significance of milk pH in newborn infants. *Br Med J* 4(5839)**,** 515-518. doi: 10.1136/bmj.4.5839.515.

Hennessy, T.G., MacDonald, D., Hennessy, M.S., Maguire, M., Blake, S., McCann, H.A., et al. (1996). Serial changes in cardiac output during normal pregnancy: a Doppler ultrasound study. *European Journal of Obstetrics & Gynecology and Reproductive Biology* 70(2)**,** 117-122.

Herngren, L., Ehrnebo, M., and Boreus, L. (1983). Drug binding to plasma proteins during human pregnancy and in the perinatal period. Studies on cloxacillin and alprenolol. *Developmental Pharmacology and Therapeutics* 6(2)**,** 110-124.

Hladunewich, M., Lafayette, R., Derby, G., Blouch, K., Bialek, J., Druzin, M., et al. (2004). The dynamics of glomerular filtration in the puerperium. *American Journal of Physiology-Renal Physiology* 286(3)**,** F496-F503.

Honda, M., Omori, Y., Minei, S., Oshiyama, T., Shimizu, M., Sanaka, M., et al. (1990). Quantitative analysis of serum α1-acid glycoprotein levels in normal and diabetic pregnancy. *Diabetes research and clinical practice* 10(2)**,** 147-152.

Houston, M., Howie, P., and McNeilly, A. (1983). Factors affecting the duration of breast feeding: 1. Measurement of breast milk intake in the first week of life. *Early Human Development* 8(1)**,** 49-54.

Hsu, Y.C., Chen, C.H., Lin, M.C., Tsai, C.R., Liang, J.T., and Wang, T.M. (2014). Changes in preterm breast milk nutrient content in the first month. *Pediatr Neonatol* 55(6)**,** 449-454. doi: 10.1016/j.pedneo.2014.03.002.

Huang, Z., and Hu, Y.M. (2020). Dietary patterns and their association with breast milk macronutrient composition among lactating women. *Int Breastfeed J* 15(1)**,** 52. doi: 10.1186/s13006-020-00293-w.

Hunter, S., and Robson, S.C. (1992). Adaptation of the maternal heart in pregnancy. *British heart journal* 68(6)**,** 540.

Hunyor, S. (1984). Vascular, volume, and cardiac response to normal and hypertensive pregnancy. *Hypertension* 6(6_pt_2)**,** III129.

Hunyor, S., Saunders, D., Bellamy, G., Roffe, D., Harford, E., and Helfgott, A. (1982). Venous and volume factors in women during and after normotensive pregnancy. *Clinical and Experimental Pharmacology & Physiology* 9(3)**,** 315-320.

Hytten, F. (1954). Clinical and Chemical Studies in Human Lactation.—IX. *British Medical Journal* 2(4902)**,** 1447.

Hytten, F. (1994). *Observations on human lactation.*

Hytten, F., and Paintin, D. (1963). Increase in plasma volume during normal pregnancy.

Ilett, K.F., Kristensen, J.H., Hackett, L.P., Paech, M., Kohan, R., and Rampono, J. (2002). Distribution of venlafaxine and its O-desmethyl metabolite in human milk and their effects in breastfed infants. *Br J Clin Pharmacol* 53(1)**,** 17-22. doi: 10.1046/j.0306-5251.2001.01518.x.

Katz, R., Karliner, J.S., and Resnik, R. (1978). Effects of a natural volume overload state (pregnancy) on left ventricular performance in normal human subjects. *Circulation* 58(3)**,** 434-441.

Kent, J.C., Gardner, H., and Geddes, D.T. (2016). Breastmilk production in the first 4 weeks after birth of term infants. *Nutrients* 8(12)**,** 756.

Kent, J.C., Gardner, H., Lai, C.-T., Hartmann, P.E., Murray, K., Rea, A., et al. (2018). Hourly breast expression to estimate the rate of synthesis of milk and fat. *Nutrients* 10(9)**,** 1144.

Kent, J.C., Hepworth, A.R., Sherriff, J.L., Cox, D.B., Mitoulas, L.R., and Hartmann, P.E. (2013). Longitudinal changes in breastfeeding patterns from 1 to 6 months of lactation. *Breastfeeding Medicine* 8(4)**,** 401-407.

Kent, J.C., Mitoulas, L., Cox, D.B., Owens, R.A., and Hartmann, P.E. (1999). Breast volume and milk production during extended lactation in women. *Experimental physiology* 84(2)**,** 435-447.

Kent, J.C., Mitoulas, L.R., Cregan, M.D., Ramsay, D.T., Doherty, D.A., and Hartmann, P.E. (2006). Volume and frequency of breastfeedings and fat content of breast milk throughout the day. *Pediatrics* 117(3)**,** e387-e395.

Khan, S., Hepworth, A.R., Prime, D.K., Lai, C.T., Trengove, N.J., and Hartmann, P.E. (2013a). Variation in fat, lactose, and protein composition in breast milk over 24 hours: associations with infant feeding patterns. *Journal of Human Lactation* 29(1)**,** 81-89.

Khan, S., Prime, D.K., Hepworth, A.R., Lai, C.T., Trengove, N.J., and Hartmann, P.E. (2013b). Investigation of short-term variations in term breast milk composition during repeated breast expression sessions. *J Hum Lact* 29(2)**,** 196-204. doi: 10.1177/0890334412470213.

Kiboi, W., Kimiywe, J., and Chege, P. (2020). Longitudinal Changes of Human Milk Nutrient Content in the First 6 Months of Lactation.

Kim, M.H., Shim, K.S., Yi, D.Y., Lim, I.S., Chae, S.A., Yun, S.W., et al. (2019). Macronutrient Analysis of Human Milk according to Storage and Processing in Korean Mother. *Pediatr Gastroenterol Hepatol Nutr* 22(3)**,** 262-269. doi: 10.5223/pghn.2019.22.3.262.

Klajnbard, A., Szecsi, P.B., Colov, N.P., Andersen, M.R., Jørgensen, M., Bjørngaard, B., et al. (2010). Laboratory reference intervals during pregnancy, delivery and the early postpartum period. *Clinical chemistry and laboratory medicine* 48(2)**,** 237-248.

Kociszewska-Najman, B., Borek-Dzieciol, B., Szpotanska-Sikorska, M., Wilkos, E., Pietrzak, B., and Wielgos, M. (2012). The creamatocrit, fat and energy concentration in human milk produced by mothers of preterm and term infants. *The Journal of Maternal-Fetal & Neonatal Medicine* 25(9)**,** 1599-1602.

Lafayette, R.A., Malik, T., Druzin, M., Derby, G., and Myers, B.D. (1999). The dynamics of glomerular filtration after Caesarean section. *Journal of the American Society of Nephrology* 10(7)**,** 1561-1565.

Larijani, G.E., Norris, M.C., Ala-Kokko, T.I., Leighton, B.A., and Desimone, C. (1990). Serum Concentration of Alpha,-Acid Glycoprotein and Albumin following Cesarean Section and Vaginal Delivery. *DICP* 24(3)**,** 328-329.

Larsson, A., Palm, M., Hansson, L.O., and Axelsson, O. (2008). Reference values for clinical chemistry tests during normal pregnancy. *BJOG: An International Journal of Obstetrics & Gynaecology* 115(7)**,** 874-881.

Lavie, A., Ram, M., Lev, S., Blecher, Y., Amikam, U., Shulman, Y., et al. (2018). Maternal hemodynamics in late gestation and immediate postpartum in singletons vs. twin pregnancies. *Archives of Gynecology and Obstetrics* 297**,** 353-363.

Lemons, J.A., Moye, L., Hall, D., and Simmons, M. (1982). Differences in the composition of preterm and term human milk during early lactation. *Pediatric Research* 16(2)**,** 113-117.

Lisowska-Myjak, B., Zborowska, H., Jaźwiec, R., Karlińska, M., and Skarżyńska, E. (2021). Serum indoxyl sulphate and its relation to albumin and α1-acid glycoprotein as a potential biomarkers of maternal intestinal metabolism during pregnancy and postpartum. *Plos one* 16(11)**,** e0259501.

Lubetzky, R., Mimouni, F.B., Dollberg, S., Salomon, M., and Mandel, D. (2007). Consistent circadian variations in creamatocrit over the first 7 weeks of lactation: a longitudinal study. *Breastfeeding Medicine* 2(1)**,** 15-18.

Luisa, B.G. (1995). *Handbook of milk composition.* Elsevier.

Maas, Y.G., Gerritsen, J., Hart, A.A., Hadders-Algra, M., Ruijter, J.M., Tamminga, P., et al. (1998). Development of macronutrient composition of very preterm human milk. *British journal of nutrition* 80(1)**,** 35-40.

Mabie, W.C., DiSessa, T.G., Crocker, L.G., Sibai, B.M., and Arheart, K.L. (1994). A longitudinal study of cardiac output in normal human pregnancy. *American journal of obstetrics and gynecology* 170(3)**,** 849-856.

Macy, I.G. (1949). Composition of human colostrum and milk. *Am J Dis Child (1911)* 78(4)**,** 589-603. doi: 10.1001/archpedi.1949.02030050604009.

Malhotra, S.L. (1982). Effect of non-suckling on the pH of breast milk and its possible relationship with breast cancer. *Postgraduate Medical Journal* 58(686)**,** 749-752.

Mandel, D., Lubetzky, R., Dollberg, S., Barak, S., and Mimouni, F.B. (2005). Fat and energy contents of expressed human breast milk in prolonged lactation. *Pediatrics* 116(3)**,** e432-e435.

Mashini, I.S., Albazzaz, S.J., Fadel, H.E., Abdulla, A.M., Hadi, H.A., Harp, R., et al. (1987). Serial noninvasive evaluation of cardiovascular hemodynamics during pregnancy. *American journal of obstetrics and gynecology* 156(5)**,** 1208-1213.

Masini, G., Foo, L.F., Cornette, J., Tay, J., Rizopoulos, D., McEniery, C.M., et al. (2019). Cardiac output changes from prior to pregnancy to post partum using two non-invasive techniques. *Heart* 105(9)**,** 715-720.

McLennan, C.E., and Thouin, L. (1948). Blood volume in pregnancy. A critical review and preliminary report of results with a new technique.

Meier, P.P., Engstrom, J.L., Zuleger, J.L., Motykowski, J.E., Vasan, U., Meier, W.A., et al. (2006). Accuracy of a user-friendly centrifuge for measuring creamatocrits on mothers' milk in the clinical setting. *Breastfeeding Medicine* 1(2)**,** 79-87.

Mesa, A., Jessurun, C., Hernandez, A., Adam, K., Brown, D., Vaughn, W.K., et al. (1999). Left ventricular diastolic function in normal human pregnancy. *Circulation* 99(4)**,** 511-517.

Milman, N., Bergholt, T., Byg, K.E., Eriksen, L., and Hvas, A.M. (2007). Reference intervals for haematological variables during normal pregnancy and postpartum in 434 healthy Danish women. *European journal of haematology* 79(1)**,** 39-46.

Milman, N., BYG, K.E., and Agger, A.O. (2000). Hemoglobin and erythrocyte indices during normal pregnancy and postpartum in 206 women with and without iron supplementation. *Acta Obstetricia et Gynecologica Scandinavica: ORIGINAL ARTICLE* 79(2)**,** 89-98.

Milne, J., Lindheimer, M., and Davison, J. (2002). Glomerular heteroporous membrane modeling in third trimester and postpartum before and during amino acid infusion. *American Journal of Physiology-Renal Physiology* 282(1)**,** F170-F175.

Milsom, I., Hedner, J., and Hedner, T. (1988). Plasma atrial natriuretic peptide (ANP) and maternal hemodynamic changes during normal pregnancy. *Acta obstetricia et gynecologica Scandinavica* 67(8)**,** 717-722.

Mitoulas, L.R., Kent, J.C., Cox, D.B., Owens, R.A., Sherriff, J.L., and Hartmann, P.E. (2002). Variation in fat, lactose and protein in human milk over 24h and throughout the first year of lactation. *British Journal of Nutrition* 88(1)**,** 29-37.

Moran, P., Baylis, P.H., Lindheimer, M.D., and Davison, J.M. (2003). Glomerular ultrafiltration in normal and preeclamptic pregnancy. *Journal of the American Society of Nephrology* 14(3)**,** 648-652.

Morriss Jr, F.H., Brewer, E.D., Spedale, S.B., Riddle, L., Temple, D.M., Caprioli, R.M., et al. (1986). Relationship of human milk pH during course of lactation to concentrations of citrate and fatty acids. *Pediatrics* 78(3)**,** 458-464.

Motil, K.J., Sheng, H.-P., Montandon, C.M., and Wong, W.W. (1997). Human milk protein does not limit growth of breast-fed infants. *Journal of Pediatric Gastroenterology and Nutrition* 24(1)**,** 10-17.

Nagasawa, T., Kiyosawa, I., and Takase, M. (1974). Lactoferrin and serum albumin of human casein in colostrum and milk. *J Dairy Sci* 57(10)**,** 1159-1163. doi: 10.3168/jds.S0022-0302(74)85030-7.

Nelson, D.B., Stewart, R.D., Matulevicius, S.A., Morgan, J.L., McIntire, D.D., Drazner, M., et al. (2015). The effects of maternal position and habitus on maternal cardiovascular parameters as measured by cardiac magnetic resonance. *American Journal of Perinatology* 32(14)**,** 1318-1323.

Neville, M.C., Keller, R., Seacat, J., Lutes, V., Lutes, M., Casey, C., et al. (1988). Studies in human lactation: milk volumes in lactating women during the onset of lactation and full lactation. *The American Journal of Clinical Nutrition* 48(6)**,** 1375-1386.

Nielsen, S.B., Reilly, J.J., Fewtrell, M.S., Eaton, S., Grinham, J., and Wells, J.C. (2011). Adequacy of milk intake during exclusive breastfeeding: a longitudinal study. *Pediatrics* 128(4)**,** e907-e914.

Nommsen, L.A., Lovelady, C.A., Heinig, M.J., Lönnerdal, B., and Dewey, K.G. (1991). Determinants of energy, protein, lipid, and lactose concentrations in human milk during the first 12 mo of lactation: the DARLING Study. *The American journal of clinical nutrition* 53(2)**,** 457-465.

O’Neill, E.F., Radmacher, P.G., Sparks, B., and Adamkin, D.H. (2013). Creamatocrit analysis of human milk overestimates fat and energy content when compared to a human milk analyzer using mid-infrared spectroscopy. *Journal of pediatric gastroenterology and nutrition* 56(5)**,** 569-572.

Ogueh, O., Brookes, C., and Johnson, M.R. (2009). A longitudinal study of the maternal cardiovascular adaptation to spontaneous and assisted conception pregnancies. *Hypertension in Pregnancy* 28(3)**,** 273-289.

Ogundele, M.O. (2002). Effects of storage on the physicochemical and antibacterial properties of human milk. *British journal of biomedical science* 59(4)**,** 205-211.

Oo, C.Y., Kuhn, R.J., Desai, N., Wright, C.E., and McNamara, P.J. (1995). Pharmacokinetics in lactating women: prediction of alprazolam transfer into milk. *Br J Clin Pharmacol* 40(3)**,** 231-236. doi: 10.1111/j.1365-2125.1995.tb05778.x.

Paaby, P. (1960). Changes in serum proteins during pregnancy.

Paduraru, L., Zonda, G.I., Avasiloaiei, A.L., Moscalu, M., Dimitriu, D.C., and Stamatin, M. (2019). Influence of refrigeration or freezing on human milk macronutrients and energy content in early lactation: Results from a tertiary centre survey. *Paediatr Child Health* 24(4)**,** 250-257. doi: 10.1093/pch/pxy164.

Paintin, D. (1962). The size of the total red cell volume in pregnancy. *BJOG: An International Journal of Obstetrics & Gynaecology* 69(5)**,** 719-723.

Pamblanco, M., Ten, A., and Comin, J. (1986). Proteins in preterm and term milk from mothers delivering appropriate or small-for-gestational age infants. *Early Hum Dev* 14(3-4)**,** 267-272. doi: 10.1016/0378-3782(86)90188-x.

Pecka-Kiełb, E., Zachwieja, A., Wojtas, E., and Zawadzki, W. (2018). Influence of nutrition on the quality of colostrum and milk of ruminants. *Mljekarstvo: časopis za unaprjeđenje proizvodnje i prerade mlijeka* 68(3)**,** 169-181.

Perrin, M.T., Fogleman, A.D., Newburg, D.S., and Allen, J.C. (2017). A longitudinal study of human milk composition in the second year postpartum: implications for human milk banking. *Maternal & child nutrition* 13(1)**,** e12239.

Pirani, B.B., Campbell, D.M., and MacGillivray, I. (1973). Plasma volume in normal first pregnancy. *J Obstet Gynaecol Br Commonw* 80(10)**,** 884-887. doi: 10.1111/j.1471-0528.1973.tb02146.x.

Pivarnik, J.M., Mauer, M.B., Ayres, N.A., Kirshon, B., Dildy, G.A., and Cotton, D.B. (1994). Effects of chronic exercise on blood volume expansion and hematologic indices during pregnancy. *Obstetrics & Gynecology* 83(2)**,** 265-269.

Pond, S.M., Kreek, M.J., Tong, T.G., Raghunath, J., and Benowitz, N.L. (1985). Altered methadone pharmacokinetics in methadone-maintained pregnant women. *Journal of Pharmacology and Experimental Therapeutics* 233(1)**,** 1-6.

Poppas, A., Shroff, S.G., Korcarz, C.E., Hibbard, J.U., Berger, D.S., Lindheimer, M.D., et al. (1997). Serial assessment of the cardiovascular system in normal pregnancy: role of arterial compliance and pulsatile arterial load. *Circulation* 95(10)**,** 2407-2415.

Pritchard, J.A., and Rowland, R.C. (1964). Blood volume changes in pregnancy and the puerperium: III. Whole body and large vessel hematocrits in pregnant and nonpregnant women. *American Journal of Obstetrics and Gynecology* 88(3)**,** 391-395.

Ragsdale, A.S., Thiele, L.R., Byrne, J.J., Zofkie, A.C., McIntire, D.D., and Spong, C.Y. (2022). Natural history of postpartum hematocrit recovery in an urban, safety-net population. *American journal of obstetrics & gynecology MFM* 4(2)**,** 100541.

Rajalakshmi, K., and Raman, L. (1985). Plasma volume changes in Indian women with normal pregnancy. *The Indian Journal of Medical Research* 82**,** 521-527.

Ram, M., Lavie, A., Lev, S., Blecher, Y., Amikam, U., Shulman, Y., et al. (2017). Cardiac hemodynamics before, during and after elective cesarean section under spinal anesthesia in low-risk women. *Journal of Perinatology* 37(7)**,** 793-799.

Ramsay, D., Kent, J., Hartmann, R., and Hartmann, P. (2005). Anatomy of the lactating human breast redefined with ultrasound imaging. *Journal of anatomy* 206(6)**,** 525-534.

Rang, S., de Pablo Lapiedra, B., van Montfrans, G.A., Bouma, B.J., Wesseling, K.H., and Wolf, H. (2007). Modelflow: a new method for noninvasive assessment of cardiac output in pregnant women. *American journal of obstetrics and gynecology* 196(3)**,** 235. e231-235. e238.

Rattigan, S., Ghisalberti, A.V., and Hartmann, P. (1981). Breast-milk production in Australian women. *British Journal of Nutrition* 45(2)**,** 243-249.

Rios-Leyvraz, M., and Yao, Q. (2023). The Volume of Breast Milk Intake in Infants and Young Children: A Systematic Review and Meta-Analysis. *Breastfeed Med* 18(3)**,** 188-197. doi: 10.1089/bfm.2022.0281.

Robson, S., Dunlop, W., and Hunter, S. (1987a). Haemodynamic changes during the early puerperium. *British Medical Journal (Clinical research ed.)* 294(6579)**,** 1065.

Robson, S., Dunlop, W., Moore, M., and Hunter, S. (1987b). Haemodynamic changes during the puerperium: a Doppler and M‐mode echocardiographic study. *BJOG: An International Journal of Obstetrics & Gynaecology* 94(11)**,** 1028-1039.

Robson, S.C., Boys, R.J., Hunter, S., and Dunlop, W. (1989). Maternal hemodynamics after normal delivery and delivery complicated by postpartum hemorrhage. *Obstetrics & Gynecology* 74(2)**,** 234-239.

Roderuck, C., Williams, H.H., and Macy, I.G. (1946). Metabolism of Women During the Reproductive Cycle: VIII. The Utilization of Thiamine During Lactation: Eight Figures. *The Journal of Nutrition* 32(3)**,** 249-265.

Saarela, T., Kokkonen, J., and Koivisto, M. (2005). Macronutrient and energy contents of human milk fractions during the first six months of lactation. *Acta paediatrica* 94(9)**,** 1176-1181.

Sahin, S., Ozdemir, T., Katipoglu, N., Akcan, A.B., and Kaynak Turkmen, M. (2020). Comparison of Changes in Breast Milk Macronutrient Content During the First Month in Preterm and Term Infants. *Breastfeed Med* 15(1)**,** 56-62. doi: 10.1089/bfm.2019.0141.

Saint, L., Smith, M., and Hartmann, P. (1984). The yield and nutrient content of colostrum and milk of women from giving birth to 1 month post-partum. *British Journal of Nutrition* 52(1)**,** 87-95.

Sala, C., Campise, M., Ambroso, G., Motta, T., Zanchetti, A., and Morganti, A. (1995). Atrial natriuretic peptide and hemodynamic changes during normal human pregnancy. *Hypertension* 25(4)**,** 631-636.

Salas, S.P., Rosso, P., Espinoza, R., Robert, J.A., VAIDES, G., and DONOSO, E. (1993). Maternal plasma volume expansion and hormonal changes in women with idiopathic fetal growth retardation. *Obstetrics & Gynecology* 81(6)**,** 1029-1033.

Salmenperä, L., Perheentupa, J., and Siimes, M.A. (1985). Exclusively breast-fed healthy infants grow slower than reference infants. *Pediatric Research* 19(3)**,** 307-312.

San-Frutos, L.M., Fernández, R., Almagro, J., Barbancho, C., Salazar, F., Pérez-Medina, T., et al. (2005). Measure of hemodynamic patterns by thoracic electrical bioimpedance in normal pregnancy and in preeclampsia. *European Journal of Obstetrics & Gynecology and Reproductive Biology* 121(2)**,** 149-153.

Savu, O., Jurcuţ, R., Giuşcă, S., van Mieghem, T., Gussi, I., Popescu, B.A., et al. (2012). Morphological and functional adaptation of the maternal heart during pregnancy. *Circulation: Cardiovascular Imaging* 5(3)**,** 289-297.

Saxena, A.R., Karumanchi, S.A., Fan, S.-L., Horowitz, G.L., Hollenberg, N., Graves, S., et al. (2012). Correlation of cystatin-C with glomerular filtration rate by inulin clearance in pregnancy. *Hypertension in pregnancy* 31(1)**,** 22-30.

Schwab, C., Voney, E., Ramirez Garcia, A., Vischer, M., and Lacroix, C. (2019). Characterization of the cultivable microbiota in fresh and stored mature human breast milk. *Frontiers in microbiology* 10**,** 2666.

Sever, O., Mandel, D., Mimouni, F.B., Marom, R., Cohen, S., and Lubetzky, R. (2015). Macronutrients in human milk: Colostrum lactose but not fat or protein predicts mature human milk content. *ICAN: Infant, Child, & Adolescent Nutrition* 7(3)**,** 162-165.

Sims, E.A., and Krantz, K.E. (1958). Serial studies of renal function during pregnancy and the puerperium in normal women. *The Journal of clinical investigation* 37(12)**,** 1764-1774.

Slutzah, M., Codipilly, C.N., Potak, D., Clark, R.M., and Schanler, R.J. (2010). Refrigerator storage of expressed human milk in the neonatal intensive care unit. *The Journal of pediatrics* 156(1)**,** 26-28.

Sturgiss, S., Wilkinson, R., and Davison, J. (1996). Renal reserve during human pregnancy. *American Journal of Physiology-Renal Physiology* 271(1)**,** F16-F20.

Sunarić, S., Jovanović, T., Spasić, A., Denić, M., and Kocić, G. (2016). Comparative analysis of the physicochemical parameters of breast milk, starter infant formulas and commercial cow milks in Serbia. *Acta facultatis medicae Naissensis* 33(2)**,** 101-108.

Taylor, D., and Lind, T. (1979). Red cell mass during and after normal pregnancy. *BJOG: An International Journal of Obstetrics & Gynaecology* 86(5)**,** 364-370.

Taylor, D., Phillips, P., and Lind, T. (1981). Puerperal haematological indices. *BJOG: An International Journal of Obstetrics & Gynaecology* 88(6)**,** 601-606.

Timokhina, E., Kuzmina, T., Strizhakov, A., Pitskhelauri, E., Ignatko, I., and Belousova, V. (2019). Maternal cardiac function after normal delivery, preeclampsia, and eclampsia: a prospective study. *Journal of pregnancy* 2019(1)**,** 9795765.

Tyldum, E.V., Backe, B., Støylen, A., and Slørdahl, S.A. (2012). Maternal left ventricular and endothelial functions in preeclampsia. *Acta obstetricia et gynecologica Scandinavica* 91(5)**,** 566-573.

Ueland, K., and Hansen, J.M. (1969). Maternal cardiovascular dynamics: III. Labor and delivery under local and caudal analgesia. *American Journal of Obstetrics and Gynecology* 103(1)**,** 8-18.

Van Oppen, A.C.C., Van Der Tweel, I., Alsbach, G.J., Heethaar, R.M., and Bruinse, H.W. (1996). A longitudinal study of maternal hemodynamics during normal pregnancy. *Obstetrics & Gynecology* 88(1)**,** 40-46.

Vargas, M., Vargas, E., Julian, C.G., Armaza, J.F., Rodriguez, A., Tellez, W., et al. (2007). Determinants of blood oxygenation during pregnancy in Andean and European residents of high altitude. *American Journal of Physiology-Regulatory, Integrative and Comparative Physiology* 293(3)**,** R1303-R1312.

Vázquez-Román, S., Alonso-Díaz, C., García-Lara, N., Escuder-Vieco, D., and Pallás-Alonso, C. (Year). "Medida por crematocrito del contenido calórico de la leche materna donada congelada", in: *Anales de Pediatría*: Elsevier), 185-188.

Vieira, A.A., Moreira, M.E., Rocha, A.D., Pimenta, H.P., and Lucena, S.L. (2004). Análise do conteúdo energético do leite humano administrado a recém-nascidos de muito baixo peso ao nascimento. *Jornal de Pediatria* 80**,** 490-494.

Wang, C.D., Chu, P.S., Mellen, B.G., and Shenai, J.P. (1999). Creamatocrit and the nutrient composition of human milk. *Journal of Perinatology* 19(5)**,** 343-346.

Whittaker, P.G., and Lind, T. (1993). The intravascular mass of albumin during human pregnancy: a serial study in normal and diabetic women. *BJOG: An International Journal of Obstetrics & Gynaecology* 100(6)**,** 587-592.

Whittaker, P.G., Macphail, S., and Lind, T. (1996). Serial hematologic changes and pregnancy outcome. *Obstetrics & Gynecology* 88(1)**,** 33-39.

Wright, A., Steele, P., Bennett, J., Watts, G., and Polak, A. (1987). The urinary excretion of albumin in normal pregnancy. *BJOG: An International Journal of Obstetrics & Gynaecology* 94(5)**,** 408-412.

Yeung, C.H.T., Fong, S., Malik, P.R.V., and Edginton, A.N. (2020). Quantifying breast milk intake by term and preterm infants for input into paediatric physiologically based pharmacokinetic models. *Matern Child Nutr* 16(2)**,** e12938. doi: 10.1111/mcn.12938.

Żelaźniewicz, A., and Pawłowski, B. (2019). Maternal breast volume in pregnancy and lactation capacity. *American journal of physical anthropology* 168(1)**,** 180-189.
